# The acute transcriptional response of the coral *Acropora millepora* to immune challenge: expression of GiMAP/IAN genes links the innate immune responses of corals with those of mammals and plants

**DOI:** 10.1186/1471-2164-14-400

**Published:** 2013-06-14

**Authors:** Yvonne Weiss, Sylvain Forêt, David C Hayward, Tracy Ainsworth, Rob King, Eldon E Ball, David J Miller

**Affiliations:** 1 ARC Centre of Excellence for Coral Reef Studies, James Cook University, Townsville, QLD 4811, Australia; 2 School of Pharmacy and Molecular Sciences, James Cook University, Townsville, QLD 4811, Australia; 3 Evolution, Ecology and Genetics, Research School of Biology, Australian National University, Bldg. 46, Canberra, ACT 0200, Australia; 4 GeneWorks Pty Ltd, 39 Winwood Street, Thebarton, SA 5031, Australia

**Keywords:** Innate immunity, Evolution, GTPase, Coral disease, Cnidaria, Transcriptomics

## Abstract

**Background:**

As a step towards understanding coral immunity we present the first whole transcriptome analysis of the acute responses of *Acropora millepora* to challenge with the bacterial cell wall derivative MDP and the viral mimic poly I:C, defined immunogens provoking distinct but well characterised responses in higher animals.

**Results:**

These experiments reveal similarities with the responses both of arthropods and mammals, as well as coral-specific effects. The most surprising finding was that MDP specifically induced three members of the GiMAP gene family, which has been implicated in immunity in mammals but is absent from *Drosophila* and *Caenorhabditis*. Like their mammalian homologs, GiMAP genes are arranged in a tandem cluster in the coral genome.

**Conclusions:**

A phylogenomic survey of this gene family implies ancient origins, multiple independent losses and lineage-specific expansions during animal evolution. Whilst functional convergence cannot be ruled out, GiMAP expression in corals may reflect an ancestral role in immunity, perhaps in phagolysosomal processing.

## Background

Understanding immune responses in corals represents a convergence of two very different research agendas. Firstly, as early diverging animals, corals and their relatives provide novel perspectives on the evolution of immune systems. Secondly, on a more practical level, the hope is that understanding immune responses may provide insights into, and ways of managing, the coral diseases that are devastating many reefs.

Coral diseases are contributors to the global decline of reefs, and there is a perception that these often act synergistically with other stressors in bringing about coral mortality (for example, [1]). For some coral diseases, bacteria have been implicated as causative agents [2,3], whilst black band disease is thought to reflect necrosis of the coral tissue underlying a microbial mat [4]. Surprisingly little is known about immune mechanisms in corals or other cnidarians, but it is clear that anthozoans have homologs of much of the innate immune repertoire of mammals, including Toll/TLR and complement pathway components [5-7] and NODs/NLRs [8,9]. One of the most intriguing findings to emerge from comparisons between the coral *Acropora* and the sea anemone *Nematostella*, the two anthozoan cnidarians for which whole genome sequence data are available, is the relative complexity of the predicted immune repertoire of the coral [7]. Many domains associated with immune functions in higher animals (Bilateria) are over-represented in the coral by comparison with the sea anemone. For example, where *Nematostella* has a single canonical Toll-like receptor [5], *Acropora* has at least four [7], and the NACHT domain complexity of *Acropora* is at least an order of magnitude greater than those of *Nematostella* or man [7] and includes novel domain combinations [9].

Although there is a large literature on coral disease, studies to date have been largely descriptive. Symptoms have been described and associated microorganisms sequenced, but in only one case has a bacterium isolated from a coral been unequivocally established as a causative agent by reinfecting a coral and reproducing the symptoms [10]. Progress has been inhibited by the difficulty of culturing many coral-inhabiting bacteria, but the field is now rapidly moving forward (reviewed in [11,12]). Similarly, reports on the innate immune responses of corals to damage or infection have proliferated in recent years (reviewed by Mydlarz et al [13] and Palmer and Traylor-Knowles [14]). As the latter have pointed out, the innate immune response involves three steps: (1) recognition, (2) transmission of this recognition via signaling pathways to effectors, and (3) an effector response. There has been progress in studying each of these steps. Candidate pattern recognition receptors have been identified by homology searching of genome and transcriptome data for Toll-like receptors, integrins and lectins and, in the case of the *A. millepora* lectin gene Millectin, upregulation has been demonstrated in response to immune challenge [15]. The identification of complement C3 homologs in *Acropora millepora* and *Porites lobata*, has been interpreted as “indicative of lectin-mediated cellular immune functions” [14].

Phenoloxidase (PO) activities have been demonstrated in many corals, and roles for these have been proposed in generating bactericidal radicals as well as in melanin synthesis [16]. Melanin resulting from PO-catalysed polymerization of phenolic compounds can encapsulate pathogens and/or wall off damaged cells, and its synthesis in response to injury or infection has been demonstrated in a number of corals (reviewed in [14]. In *Porites cylindrica*, an early response to injury is plug formation by degranulation of melanin-containing epithelial cells, followed by infiltration of the area by migratory amoebocytes that are thought to add collagen to the plug, leading to speculation that corals use immune cells and wound healing processes similar to those of higher animals [17]. Transglutaminase activity, which in higher animals has a coagulation function and thus could contribute to wound sealing, has also been demonstrated in *Porites cylindrica*[16].

Whereas the work discussed above was based largely on candidate genes and pathways, Vidal-Dupiol et al. [18,19] took a different approach, using subtractive hybridization to identify *Pocillopora damicornis* genes regulated in response to infection with *Vibrio coralliiltycus*. This approach resulted in the discovery of Damicornin [19], the first anti-microbial peptide (AMP) to be identified from a coral. Although no other AMPs produced by corals have been identified to date, there is mounting evidence that some of the microbes normally found in the mucus of healthy corals may produce peptides that hold other, harmful, bacteria in check (reviewed in [12]).

For the staghorn coral *Acropora millepora*, a “near complete” transcriptome assembly is available [20], permitting comprehensive and relatively unbiased analyses of coral immune responses. To better understand how corals respond to immune challenge, we exposed single colonies of *A. millepora* to muramyl dipeptide (MDP) and polyinosinic:polycytidylic acid (poly I:C), two defined immunogens provoking distinct but well characterised responses in higher animals, and then determined the acute response at the whole transcriptome level using high throughput sequencing (Illumina RNA-seq). MDP is a minimal common peptidoglycan constituent of both Gram positive and Gram negative bacteria, whereas poly I:C is essentially a viral mimic due to its structural similarity to double stranded RNA. In mammals, the immune responses to these compounds are fairly well understood, providing a reference against which the molecular responses of coral can be compared. In the mouse, MDP-like compounds released as a result of phagolysosomal processing of bacteria are thought to be ligands of the NACHT-domain protein NOD2 [21], activating NF-kb signaling [22] and leading to expression of AMPs and cytokines [23]. Poly I:C is a ligand for the murine TLR3 receptor, activating MAP-kinases and NF-kb by distinct molecular pathways [24]. In *Drosophila*, NF-kb dependent expression of AMPs is triggered by infection by diverse infective agents through the Toll receptor (reviewed in [25]), but NOD-like proteins are not present. *Drosophila* Toll-7 has recently been shown to be a pattern recognition receptor for a viral ligand, inducing antiviral autophagy independently of both NF-kb and JAK-STAT pathways [26].

Comparative analyses of MDP and poly I:C challenged corals reveals similarities with the responses both of arthropods and mammals, as well as coral-specific effects. Three genes belonging to the GiMAP/IAN family, associated with immunity in mammals, were amongst the genes most highly up-regulated upon MDP challenge. This ancient gene family has a patchy distribution across the animal kingdom that is characterised by many independent losses and lineage-specific expansions. Although convergence cannot be ruled out, GiMAP expression during immune challenge in coral may reflect an ancient function, perhaps in phagolysosomal processing.

## Results

### The molecular responses to challenge with MDP or poly I:C are largely discrete

**Figure 1:**
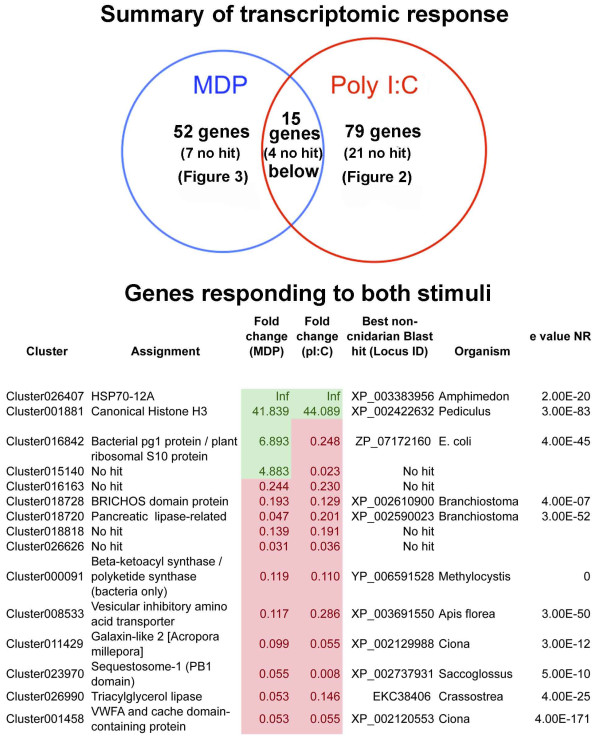
**The transcriptomic responses of coral to immune challenge.** (Upper) The acute transcriptomic responses to MDP and Poly I:C are largely discrete. Relatively little overlap was observed between the responses to the bacterial cell-wall derivative MDP and the dsRNA mimic Poly I:C. “No hit” indicates that no significant matches were detected by BlastX searching of Genbank using E-5 as the significance cutoff. (Lower) The 15 transcriptome clusters differentially regulated under both MDP and Poly I:C challenge; up-regulated clusters are shown in green, down-regulation is indicated in red. BlastX comparisons were carried out against the NR database via NCBI using a cutoff of E-5.

For each treatment, relatively few genes were identified as differentially regulated using an adjusted p-value cut-off of 5% (Figure 1 and Additional file 1). One reason for this is the large variation in responses of individual corals that has previously been observed in *A. millepora*[27,28]. The sets of genes differentially regulated by MDP and poly I:C are largely discrete: only 15 genes were differentially expressed in response to both treatments (Figure 1), with few known genes responding to challenge with MDP and poly I:C in the same way. Amongst these, Cluster001881, which encodes the core histone H3 [29], was strongly up-regulated relative to controls. Cluster026407, which encodes an HSP12/ORP150-related chaperone, was the most strongly up-regulated gene under both treatments. The HSP12/ORP150 proteins are a group of atypical members of the HSP70 family [30] that are widely distributed across the Metazoa (but not present in *Drosophila* or *Caenorhabditis*), and known to play a protective role in vertebrate hypoxia and immune responses [31-33].

A general suppression of calcification under immune challenge is implied by the down-regulation of galaxin-like 2 (Cluster011429) and cluster001458, which encodes a voltage gated calcium channel-like VWA protein. Likewise, the down-regulation of Cluster000091, which encodes a multi-domain fatty acid synthase protein, and lipases (Clusters 026990 and 018720) suggests a general suppression of metabolism under immune challenge, as is also seen under acute acidification [20].

A Gene Ontology enrichment analysis of the response to each immunogen indicates that in both treatments a single category (GO:0070199; establishment of protein localization to chromosome, in the Biological Process ontology, p < 0.05) is significantly overrepresented. This is consistent with the differential expression of histones and zinc finger proteins outlined below.

### Specific responses to challenge with poly I:C

**Figure 2:**
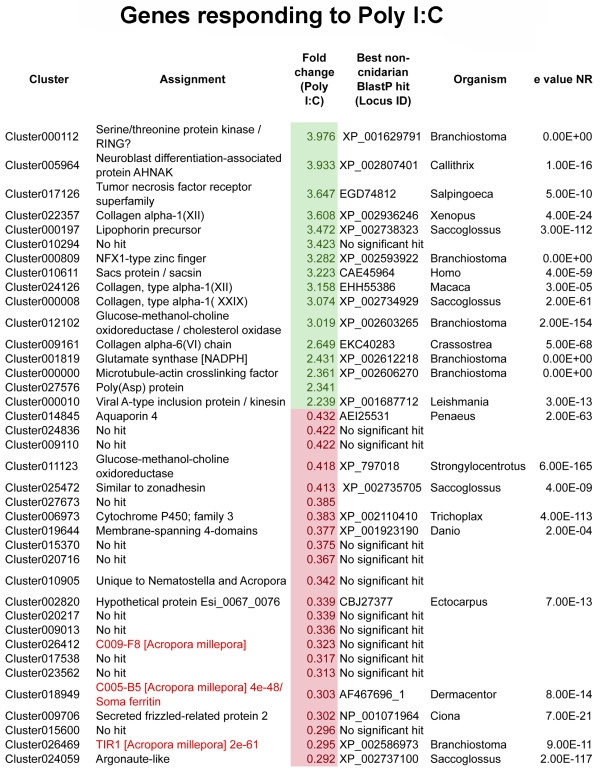
**Transcriptome clusters differentially regulated under Poly I:C challenge; up-regulated clusters are shown in green, down-regulation is indicated in red.** BlastX comparisons were carried out against the NR database via NCBI using a cutoff of E-5. For simplicity, only the 48 genes whose expression changed most are listed; for complete results and comparisons to *A. digitifera* and *Nematostella*, see Additional file 2.

The poly I:C response is predominantly negative, only 16 genes being specifically up-regulated compared to 63 specifically down-regulated (Figure 2 and Additional file 2). The up-regulated genes include a number of collagens, NADP-type glutamate synthase (Cluster001819), a putative TNF-receptor protein (Cluster017126) distinct from that mentioned above (cluster016163), an NFX-type zinc finger protein (Cluster000809), and a lipophorin (Cluster000197). Genes with a wide range of predicted functions are down-regulated, but amongst these are a number encoding RNA-binding or processing activities; for example, an argonaute protein (Cluster024059) and an RNA-helicase (Cluster023450).

### Specific responses to challenge with MDP

**Figure 3:**
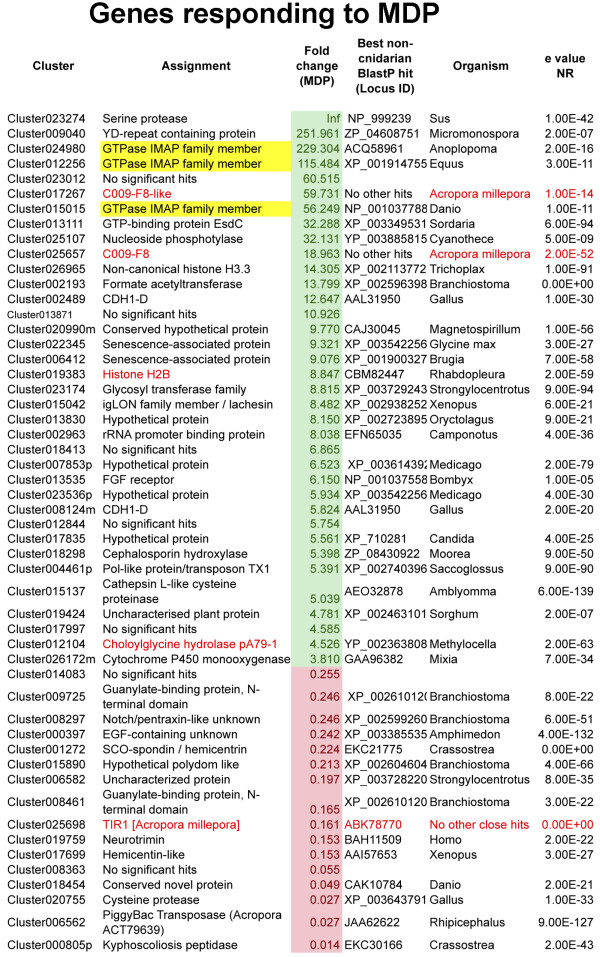
**Transcriptome clusters differentially regulated under MDP challenge; up-regulated clusters are shown in green, down-regulation is indicated in red.** The GiMAP genes are highlighted in yellow. BlastX comparisons were carried out against the NR database via NCBI using a cutoff of E-5. For comparisons to *A. digitifera* and *Nematostella*, see Additional file 3.

By contrast with the effect of poly I:C treatment, the acute transcriptional response to MDP was predominantly positive: 36 genes were specifically up-regulated and 16 specifically down-regulated in this treatment (Figure 3 and Additional file 3). The known *Acropora* genes histone H2B [34] and the pA79-1 choloylglycine hydrolase [35] were up-regulated specifically by MDP, as was an unambiguous ortholog of the histone 3 variant H3.3 (cluster026965). After MDP challenge, the most highly up-regulated gene (Cluster023274) encodes a serine protease similar to kallikrein. A number of proteins containing immune-related domains were amongst those up-regulated; Cluster023174 encodes an O-linked-mannose beta-1,2-N-acetylglucosaminyltransferase, Cluster015042 encodes two IgC2-type domains and Cluster026172m encodes a cytochrome P-450 family member. Decreases in the expression levels of other immune-related proteins (egs. clusters 000397, 008297, 001272, 015890), including the known TIR1 protein (database accession EF090256); [5] were also observed. Note that this TIR protein has a predicted extracellular domain but lacks the LRRs that characterise canonical TLR proteins.

### Coral members of the GiMAP/IAN family are up-regulated by the bacterial PAMP MDP but not by poly I:C

**Figure 4:**
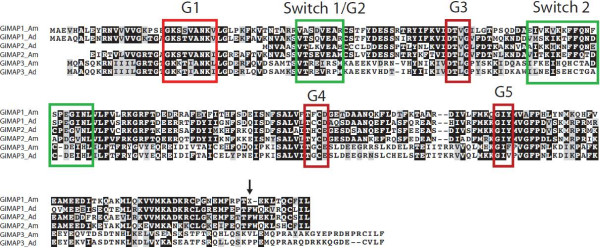
**Predicted amino acid sequences of the *Acropora *GiMAP proteins responding to challenge with the bacterial cell wall derivative MDP.** The figure shows the proteins encoded by Cluster012256 (GiMAP1), Cluster024980 (GiMAP2) and Cluster015015 (GiMAP3) from the *A. millepora* transcriptome assembly, aligned with their putative orthologs from the related species *A. digitifera* (aug_v2a23822t1, aug_v2a23824t1 and aug_v2a23823t1 respectively). Boxed regions correspond to conserved structural features of the AIG domain (cd01852; pfam04548) based on alignment of the coral sequences with the 2XTM_A (GDP-bound human GiMAP2) and 3LXX_A (human GiMAP4) crystal structures using Cn3D v4.1 [40]. Note that in the case of GiMAP1_Am, the protein predicted from transcriptome Cluster012256 terminates at the “X” at position 223 indicated by the arrow, and inspection of individual Illumina reads did not resolve the ambiguity. However, extending the translation resulted in a further 9 AA region matching the *A. digitifera* presumed ortholog and GiMAP2 sequences in both species.

Three of the ten genes that were most highly up-regulated after MDP challenge are distinct homologs of the GTPases of immunity associated proteins (GiMAPs; [36]), which are also known as immune-associated nucleotide-binding proteins (IANs; [37]). Each of these three genes was up-regulated >50-fold by MDP, but transcript levels were not significantly affected following poly I:C treatment. Up-regulation of GiMAP-related proteins in coral after challenge with a bacterial cell wall derivative is intriguing, because these proteins have been implicated in the immune responses to bacteria of both vertebrates (reviewed in [38]) and higher plants (reviewed in [39]). The GiMAP/IANs are a distinct group within the P-loop NTPase superfamily (NCBI PSSM Id 214148), defined by the presence of the AIG1 GTP-binding domain (NCBI CDD cd01852). For each of the three *A. millepora* GiMAP-like predicted proteins, the NTPase domain matches best the AIG1 model pfam04548 (e value < E-12 in each case; Cluster012256 (GiMAP1) = 9.57E-14; Cluster 024980 (GiMAP2) = 1.53E-19; Cluster015015 (GiMAP3) = 5.58E-13), whereas the significance values for matches to other related domains (e.g. Toc-34, Ras, Septin) were much lower (Additional file 4). The coral GiMAPs (Figure 4) are small proteins and appear to lack the C-terminal extensions that are typical of their vertebrate counterparts, and which often contain coiled-coil and/or hydrophobic domains. Although the catalytic residues and other defining features (switch region I, switch region II, G1-G5 boxes) were present in each of the coral GiMAPs, we were unable to identify regions with sequence similarity to the conserved box (located between G3 and G4) defined in mammals.

### Structure and organisation of the coral GiMAP loci

**Figure 5:**
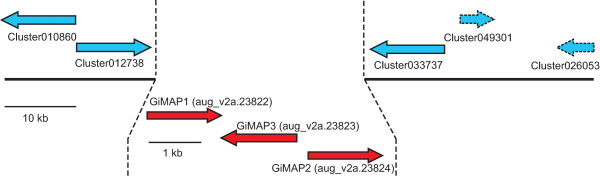
**Organisation of the coral GiMAP loci.** As shown in the upper part of the figure (in blue) the genomic context of the GiMAP loci is the same in both *A. millepora* and *A. digitifera*. Numbers on the arrows refer to the *A. millepora* transcriptome assembly [20]. Cluster010860 encodes a protein phosphatase regulatory subunit, and corresponds to the *A. digitifera* predicted protein aug_v2a.23820.t1. Cluster012738 encodes a lysosomal acid lipase, and corresponds to *A. digitifera* aug_v2a.23821.t1. Cluster033737 corresponds to aug_v2a.23825.t1, Cluster049301 corresponds to the 3′ end of aug_v2a.23826.t1 and Cluster026053 corresponds to aug_v2a.23827.t1; all lack clear orthologs in the database. As EST support is lacking, the orientation of clusters 049301 and 026053 in *A. millepora* could not be confirmed; the orientation is assumed to be as in *A. digitifera*, but is shown in broken outline to reflect the uncertainty. As shown in the lower part of the figure (in red) the *A. digitifera* orthologs of the three *A. millepora* GiMAP genes are organised in a tight cluster. In the case of *A. millepora*, the corresponding transcriptome clusters map to a region of similar size, but the organisation of the loci could not be unequivocally established. For *A. millepora*, Cluster012256 = GiMAP1, Cluster 024980 = GiMAP2 and Cluster015015 = GiMAP3) Note that the GiMAP loci are also tightly linked in mammals and plants, but are the products of independent lineage-specific expansions.

The three *Acropora* GiMAP-like genes map to a single scaffold and are tightly linked in the coral genome; Figure 5 summarises the organisation of the coral GiMAP loci. In *Acropora digitifera*, clear orthologs (see Figure 4) of the three *A. millepora* GiMAP loci constitute a 5 kb cluster; only a few hundred bases separate the coding sequences, each of which is intronless. It is highly likely that the organisation of the GiMAP loci is the same in the case of *Acropora millepora*; all of the *A. millepora* GiMAP transcripts map to a region of similar size to the *A. digitifera* locus, but this region of the genome is not sufficiently well assembled to unequivocally confirm the organisation shown. Clearly orthologous genes flank the GiMAP loci in the two *Acropora* species (Figure 5). The clustered organisation of the GiMAP loci in coral parallels the situation in mammals and plants. The human and mouse genomes each contain eight clustered GiMAP loci whereas *Arabidopsis* has 13 IAN loci. These are organised into nine gene and three gene clusters on chromosome 1 and 4 respectively, and a single gene on chromosome 2 [41]. The clustered organisation of these genes in each organism suggests relatively recent lineage-specific duplications, and phylogenetic analyses are consistent with this hypothesis, sequences clustering primarily with others from the same or closely related species.

In addition to the three genes identified in the immune stimulation experiments, searching the *A. millepora* transcriptome yielded four other sequences encoding proteins containing both an AIG1-like domain and a C-terminal Hint domain (Additional file 4). The pfam01079 Hint domain is based on an alignment of intein domains of Hedgehog proteins, implying that these coral AIG1 proteins undergo protein splicing.

### AIG1 domains are patchily distributed across the eukaryotes

**Figure 6:**
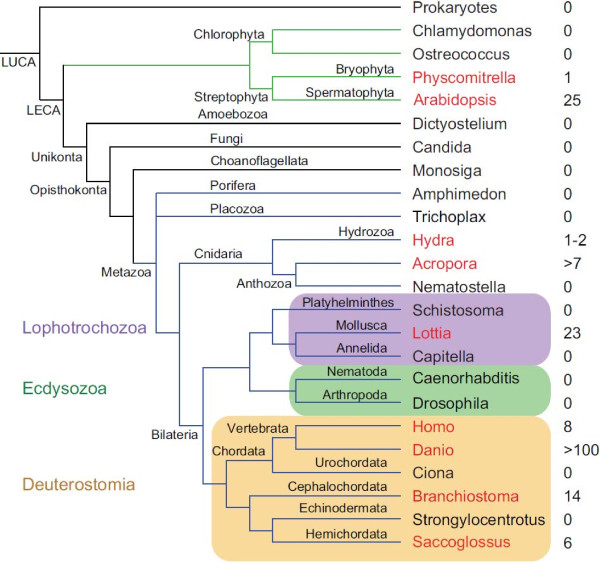
**The complex phylogenetic distribution of GiMAP/IAN-1 loci.** The figure shows results of a phylogenomic survey conducted using a combination of similarity and HMM methods. For each clade, the name of a representative genus is indicated and coloured in red if the genome of the taxon encodes GiMAP/IAN-1 domains and black if not. The numbers of domains detected are indicated to the right of the species name. Note that, in the case of the Lophotrochozoa, GiMAP/IAN-1 domains were detected only in *Lottia* (i.e. not in *Schistosoma* or *Capitella*). Abbreviations: LUCA last universal common ancestor; LECA last eukaryotic common ancestor.

**Figure 7:**
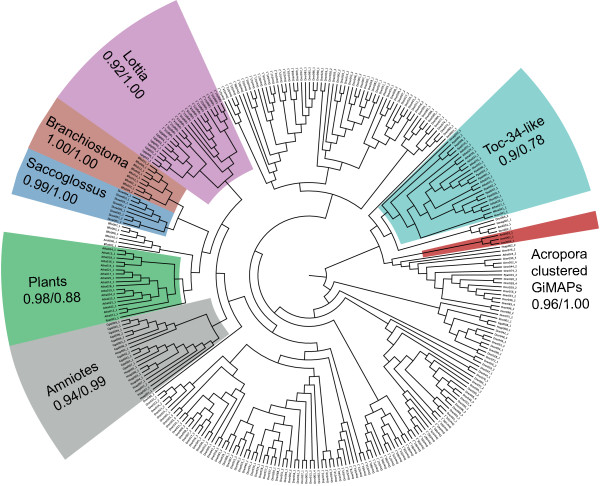
**Phylogenetic analyses imply an ancient origin and multiple independent expansions of GiMAP/IAN-1 loci during evolution.** The tree shown is the result of ML analyses, with the proportions of 100 ML bootstrap replicates and posterior probabilities (BI) shown for some of the major clades. Much of the tree is relatively poorly resolved, but (with the exception of *Danio*) most sequences for each species cluster together with high support, which is consistent with independent lineage-specific expansions. Additional files 5 and 6 respectively show the ML and BI trees with bootstrap/posterior probability support indicated for all nodes. Accession data for sequences used in the phylogenetic analyses and the alignment on which the analyses were based are shown as Additional files 7 and 8, respectively.

As outlined above, to date GiMAP/IAN proteins have been described only in vertebrates and higher plants [37]. On the basis of the identification of clearly related proteins in the coral, a broad phylogenomic survey of the gene family was undertaken, the results of which are summarised as Figures 6 and 7. Surprisingly, orthologs of the clustered *Acropora* GiMAP genes could not be identified in either *Nematostella* or *Hydra*, the two other cnidarians for which whole genome sequences are available. Two weak matches to the IAN-1 domain were, however, identified in *Hydra* (see Additional file 4). The broader distribution of GiMAP/AIG1 loci across the eukaryotes is extremely patchy (Figure 6). The AIG1 domain was not detected in representative fungi, protists or choanoflagellates, or in the early diverging metazoans *Amphimedon* and *Trichoplax*. Not only do *Drosophila* and *Caenorhabditis* lack AIG1 loci, but this holds also for all of the other available ecdysozoan genomes. Amongst lophotrochozoans, AIG1 loci were only detected in *Lottia gigantica*, where 23 paralogs were found. *Ciona* and *Strongylocentrotus* - representatives of the deuterostome lineages Urochordata and Echinodermata, respectively – also lacked AIG1 loci, although all vertebrates and both the cephalochordate *Brachiostoma* and the hemichordate *Saccoglossus* encoded multiple AIG1 domains. The AIG1 complement of *Danio rerio* was particularly complex, the genome containing over 100 loci.

To better understand evolutionary relationships of AIG1 sequences, maximum likelihood (ML) and Bayesian inference (BI) methods of phylogenetic analysis were applied (Figure 7 and Additional files 5 and 6). Representative plant Toc34 sequences were included in these analyses for comparative purposes. Several of the ambiguous AIG1-like sequences from animals clustered with the plant Toc34 sequences in phylogenetic analyses (Figure 7). With the exception of these Toc34-like sequences, the majority of AIG1 sequences grouped primarily by taxonomy – generally by species, although a few individual sequences did not conform to this general trend. The three coral GiMAPs that are tightly linked form a strongly supported cluster in phylogenetic analyses, but the position of this clade in the broader analysis is not clear. Representatives of other types of coral AIG1 domain were phylogenetically distinct from the three linked genes, suggesting deep divergence. The phylogenomic analyses indicate that, as has been observed for several other gene families [42-44], AIG1/GiMAP genes have undergone multiple independent losses during evolution – at least seven independent losses within the animal kingdom alone – and several lineage-specific expansions.

## Discussion

The data presented here suggest that the acute transcriptional response of coral to immune challenge has some elements in common not only with mammals but also, perhaps more unexpectedly, with arthropods, as well as unique components. For example, in arthropods, an early response to challenge is proteolytic activation of pro-polyphenol oxidase (proPO) via a serine proteinase cascade (reviewed in [45]); we hypothesise that the trypsin-like serine protease (Cluster023274) that is highly up-regulated after MDP challenge may play an analogous role in the proPO activation observed upon damage to *A. millepora* and a species of *Porites*[46]. Other coral genes up-regulated following MDP challenge have homologs characterised in the context of vertebrate immunity, the standout example being the three *Acropora* GiMAP/IAN genes. The pattern of up-regulation in response to the defined immunogen MDP suggests roles for the coral GiMAPs in the immune response to bacteria.

It is unclear why the AIG1 domain that characterises GiMAP proteins is present in corals but appears to have been lost in other cnidarians. Several other domains associated with immunity in higher animals are over-represented in coral compared to *Nematostella*; for example, the coral repertoires of both Toll-like receptors and NACHT domain proteins are more extensive and complex than those of the sea anemone [7], possibly reflecting the requirements of the symbiotic lifestyle of coral.

Understanding the roles of GiMAPs in coral immunity is complicated not only by the difficulty of coral as an experimental system but also by the fact that little is known about how the corresponding proteins function in vertebrates. A number of the mammalian GiMAP family proteins function in B- and T-cell development, maturation and selection; several interact with specific Bcl2 family members, participating in the regulation of apoptosis during lymphocyte development [47]. GiMAP4/IAN1 interacts with the pro-apoptotic Bax protein, whilst GiMAP3/IAN4 and GiMAP5/IAN5 interact specifically with the anti-apoptotic proteins Bcl2 and Bcl-xL [47]. It has been suggested that these interactions occur via the extended C-terminal domains of the mammalian GiMAP proteins [47], but this remains to be verified. Plant homologs of the mammalian GiMAPs are known [37], and several of these have been implicated in immunity; in *Arabidopsis*, AIG1 (AtIAN8) is induced by both the plant pathogenic bacterium *Pseudomonas syringae*[48] and the fungal pathogen *Phytophthora infestans*[41], and atIAN3 and atIAN11 are strongly up-regulated on nematode infection [41]. However, *Arabidopsis* IANs are also induced by abiotic stressors such as heat or cold [39,41], suggesting general stress-response roles rather than specific functions in immunity. Moreover, there are no clear precedents for functional conservation between plant and animal immune systems, and convergence has often been mistaken for conservation. For example, the same combinations of domains function in pattern recognition in both plants and animals; the R-proteins implicated in plant immunity share domains in common with both the Toll/TLR and NLR proteins that are the primary pattern recognition molecules in animals. Whereas this was once thought to reflect conservation of function, it is now viewed as convergence [49,50], most likely driven by the limited range of domains that can function in pattern recognition.

Although the known roles of the mammalian GiMAPs - regulation of the survival and proliferation of lymphocyte lineages - undoubtedly reflect vertebrate-specific functions, this does not preclude older and more widely conserved functions for GiMAP proteins. If conserved roles are assumed, one possibility is that GiMAPs function at the level of phagolysosomal processing, which is a universal requirement for animal immune responses. The autophagy pathway has essential roles in immune responses (reviewed in [51]) and in mammals, several other GTPases have critical roles in the induction of autophagy, phagosome maturation and the destruction of pathogens contained in vacuoles [52]. Moreover, the structural properties of the GiMAP proteins are consistent with potential roles in membrane trafficking at phagolysosomal membranes, or perhaps (by analogy with septin “caging”; [53]) in constraining and compartmentalising pathogens within the cell. Consistent with an ancient role of this sort for the GiMAP family, mammalian GiMAP5 has been shown to be associated with lysosomes [54]. Hence, although neither derived functions nor convergence can be ruled out, the up-regulation observed in corals during immune challenge may reflect an ancestral function of this kind for the GiMAP gene family in the animal kingdom.

## Conclusions

During the acute response of the coral *A. millepora* to MDP challenge, three genes encoding P-loop NTPases of the GiMAP/IAN-type were strongly up-regulated, raising the possibility of common roles in coral and mammal (and possibly also plant) immunity. A phylogenomic survey of the GiMAP gene family implies ancient origins, multiple independent losses and lineage-specific expansions during animal evolution. Whilst functional convergence cannot be ruled out, GiMAP expression in corals may reflect an ancestral role in immunity, perhaps in phagolysosomal processing.

## Methods

### Coral manipulation

Colonies of *Acropora millepora* (<40 cm diameter) were collected from the reef flat adjacent to Heron Island on the Southern Great Barrier Reef (23.44°S, 151.91°E), and transported to Heron Island Research Station where they were acclimated in 1000 L raceways under constant flow-through seawater for a period of 5 days prior to immune challenge. Control colonies (injected with buffer only; n = 4) were held in a separate raceway from colonies that were to be injected with defined immunogens (n = 4 per immunogen). Prior to use, immunogens (from InvivoGen, San Diego, CA 92121) MDP (Cat# tlrl-mdp) or poly I:C (Cat# tlrl-pic) were dissolved in sterile 3X phosphate buffered saline (pH7.4) at concentrations of 10 μg/ml. For each colony treated, a single lateral polyp within 1.5 cm of the apical tip was injected with a 200 μl aliquot of immunogen via a 27G needle. One hour after exposure, the 3 cm branch tip including the injection site was broken from the colony and snap frozen in liquid nitrogen prior to storage at -80°C. Coral manipulations were carried out under GBRMPA permit G08/24594.1.

### RNA extraction and high throughput sequencing

mRNA was isolated as previously described [20]. RNA-seq libraries were prepared and sequenced by GeneWorks Pty (Australia) on an Illumina Genome Analyzer I. For each condition (control, MDP and pIC), four libraries of single end 35 bp sequences were obtained, yielding an average of 2.8 million reads per library. These samples are biological replicates, coming from different coral colonies. The sequencing data have been deposited in the GEO database with accession ID GSE46389. The reads were mapped onto the latest *A. millepora* transcriptome assembly [20] using the Bowtie mapping software v0.12.7 [55]. Differential gene expression was inferred based on these counts using the edgeR package with common dispersion estimates [56], comparing each treatment (MDP and pIC) to the controls. P-values for differential gene expression were corrected for multiple testing using the Benjamini and Hochberg method [57], and an adjusted p-value threshold of 0.05 was used.

GO annotations were as previously used [20], and GO-enrichment analyses were carried out with the Goseq package [58].

### Phylogenomics

GiMAPs were identified with BlastP and HMMER (hmmer.org) searches, using the AIG1 domain (pfam04548), focusing on species for which sequenced well- annotated whole genome data are available. In equivocal cases, assignments were made on the basis of a BLAST e-value difference of at least 1E-4 between similarity to the AIG1 domain and alternatives. Sequences were aligned with MAFFT 6.717b [59] using the accurate L-INS-I method. Positions containing over 95% gaps were removed from the alignment. The dataset and the alignment used for phylogenetic analyses are provided as Additional files 7 and 8. Maximum likelihood trees were inferred with PhyML 3.0 [60] using the LG amino acid substitution model [61], with four substitution rate categories approximating a gamma distribution whose rate was estimated, and an invariant category. The starting trees were computed using BioNJ and the topologies were optimised by nearest neighbour interchange and sub-tree pruning and regrafting. The branch support was estimated using approximate likelihood tests [62] and with the bootstrap procedure, using 100 replicates. For Bayesian Inference, Mr Bayes 3.2-cvs was used as described in Forêt et al. [43].

## Abbreviations

AMP: Anti-microbial peptide; GiMAP: GTPases of immunity associated proteins; IAN: Immune-associated nucleotide-binding proteins; MDP: Muramyl dipeptide-a bacterial cell wall derivative; NACHT: One of three characteristic domains of NLRs; NLR: NOD-like receptor; NOD: Nucleotide Oligomerisation Domain receptors-cytoplasmic proteins that regulate inflammatory and apoptotic responses; PAMP: Pathogen-associated molecular pattern; poly I:C: Polyinosinic:polycytidylic acid-a viral mimic; TLR: Toll-like receptor- the main extracellular pattern receptors of animals, hence proteins that play a key role in the innate immune response.

## Competing interests

The authors declare that they have no competing interests.

## Authors’ contributions

DM, SF and YW conceived and designed the experiments, YW and TA carried out the experiments, SF, YW, DH, RK and DM analysed the data, and DM, EB and SF wrote the paper. All authors approved the final version of the manuscript.

## Supplementary Material

Additional file 1 **Transcriptome clusters differentially regulated under both MDP and Poly I:C challenge; up-regulated clusters are shown in green, down-regulation is indicated in red.** BlastX comparisons were carried out against the adi_aug101220 *Acropora digitifera* predicted protein set, or v1.0 of the *Nematostella vectensis* protein predictions via the OIST and JGI genome browsers respectively, or against the NR database via NCBI using a cutoff of E-5. Click here for file

Additional file 2 **Transcriptome clusters differentially regulated under Poly I:C challenge; up-regulated clusters are shown in green, down-regulation is indicated in red.** BlastX comparisons were carried out against the adi_aug101220 *Acropora digitifera* predicted protein set, or v1.0 of the *Nematostella vectensis* protein predictions via the OIST and JGI genome browsers respectively, or against the NR database via NCBI using a cutoff of E-5. Click here for file

Additional file 3 **Transcriptome clusters differentially regulated under MDP challenge; up-regulated clusters are shown in green, down-regulation is indicated in red.** BlastX comparisons were carried out against the adi_aug101220 *Acropora digitifera* predicted protein set, or v1.0 of the *Nematostella vectensis* protein predictions via the OIST and JGI genome browsers respectively, or against the NR database via NCBI using a cutoff of E-5. Click here for file

Additional file 4 **Domain matches for *A. millepora *and *H. magnipapillata.*** Click here for file

Additional file 5 **Maximum likelihood phylogenetic analysis of AIG1 domains.** Support values are indicated for all nodes. Click here for file

Additional file 6 **Bayesian inference phylogenetic analysis of AIG1 domains.** Posterior probability values indicated for all nodes. Click here for file

Additional file 7 Database accession information for all sequences used in the phylogenetic analyses. Click here for file

Additional file 8 Sequence alignments used in the phylogenetic analyses. Click here for file
