# Supplementary figures and images for: The acute transcriptional response of the coral Acropora millepora to immune challenge: expression of GiMAP/IAN genes links the innate immune responses of corals with those of mammals and plants
Source: BMC Genomics. 2013 Jun 14;14:400. doi: 10.1186/1471-2164-14-400 (PMC3723955; doi:10.1186/1471-2164-14-400)

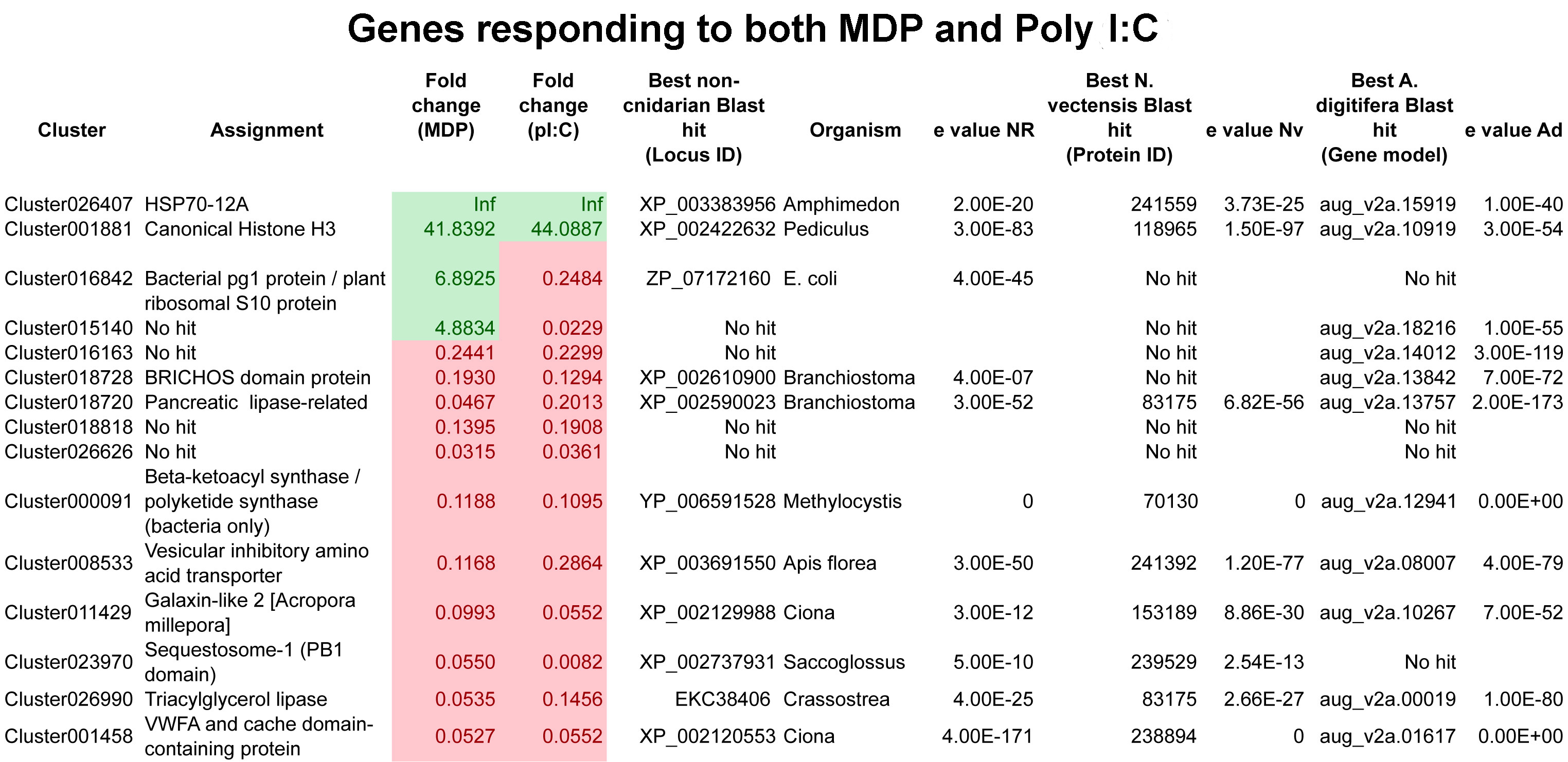

Supplement: Additional file 1 — Transcriptome clusters differentially regulated under both MDP and Poly I:C challenge; up-regulated clusters are shown in green, down-regulation is indicated in red. BlastX comparisons were carried out against the adi_aug101220 Acropora digitifera predicted protein set, or v1.0 of the Nematostella vectensis protein predictions via the OIST and JGI genome browsers respectively, or against the NR database via NCBI using a cutoff of E-5. [file 1471-2164-14-400-S1.tif]

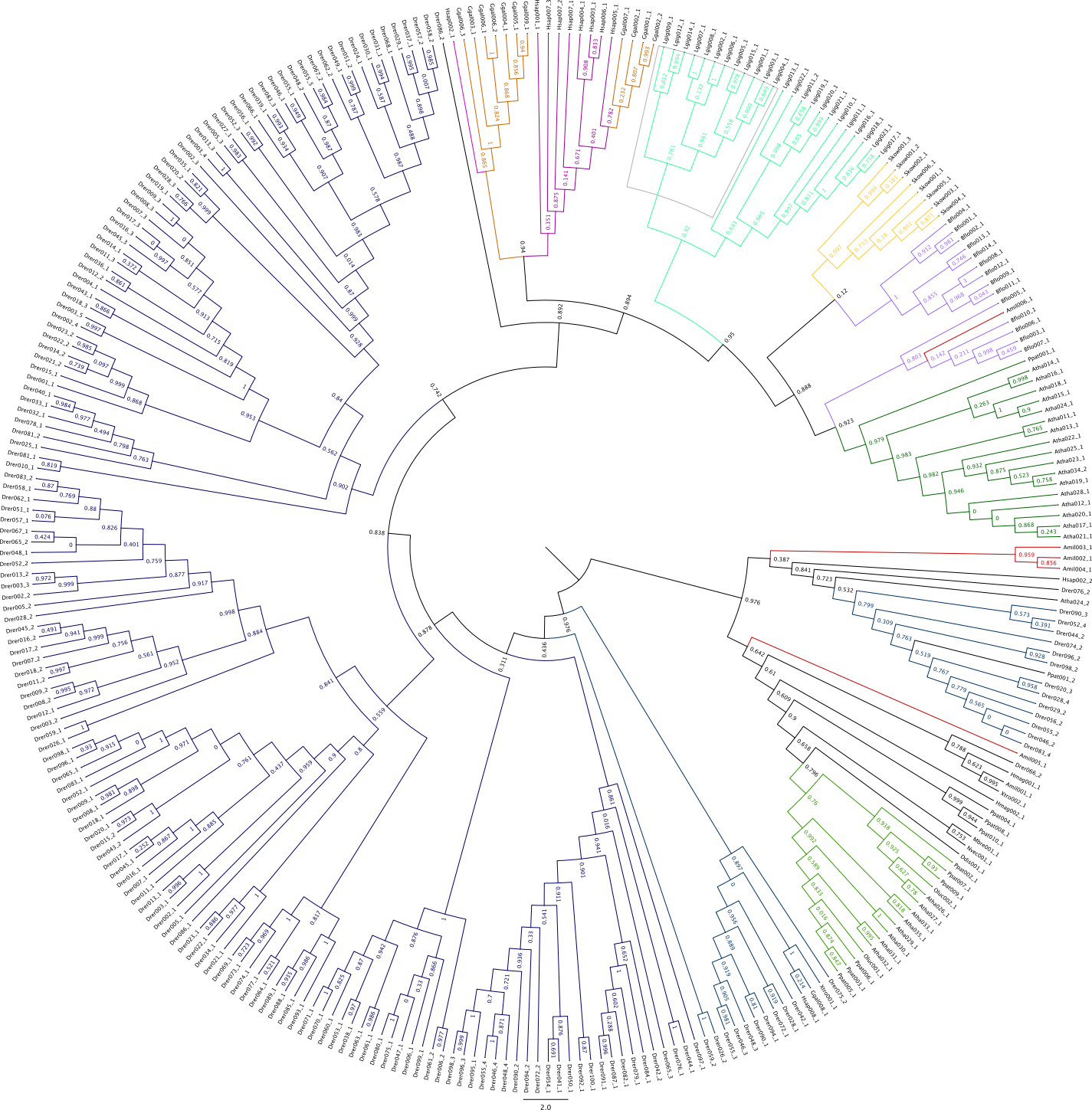

Supplement: Additional file 5 — Maximum likelihood phylogenetic analysis of AIG1 domains. Support values are indicated for all nodes. [file 1471-2164-14-400-S5.pdf]

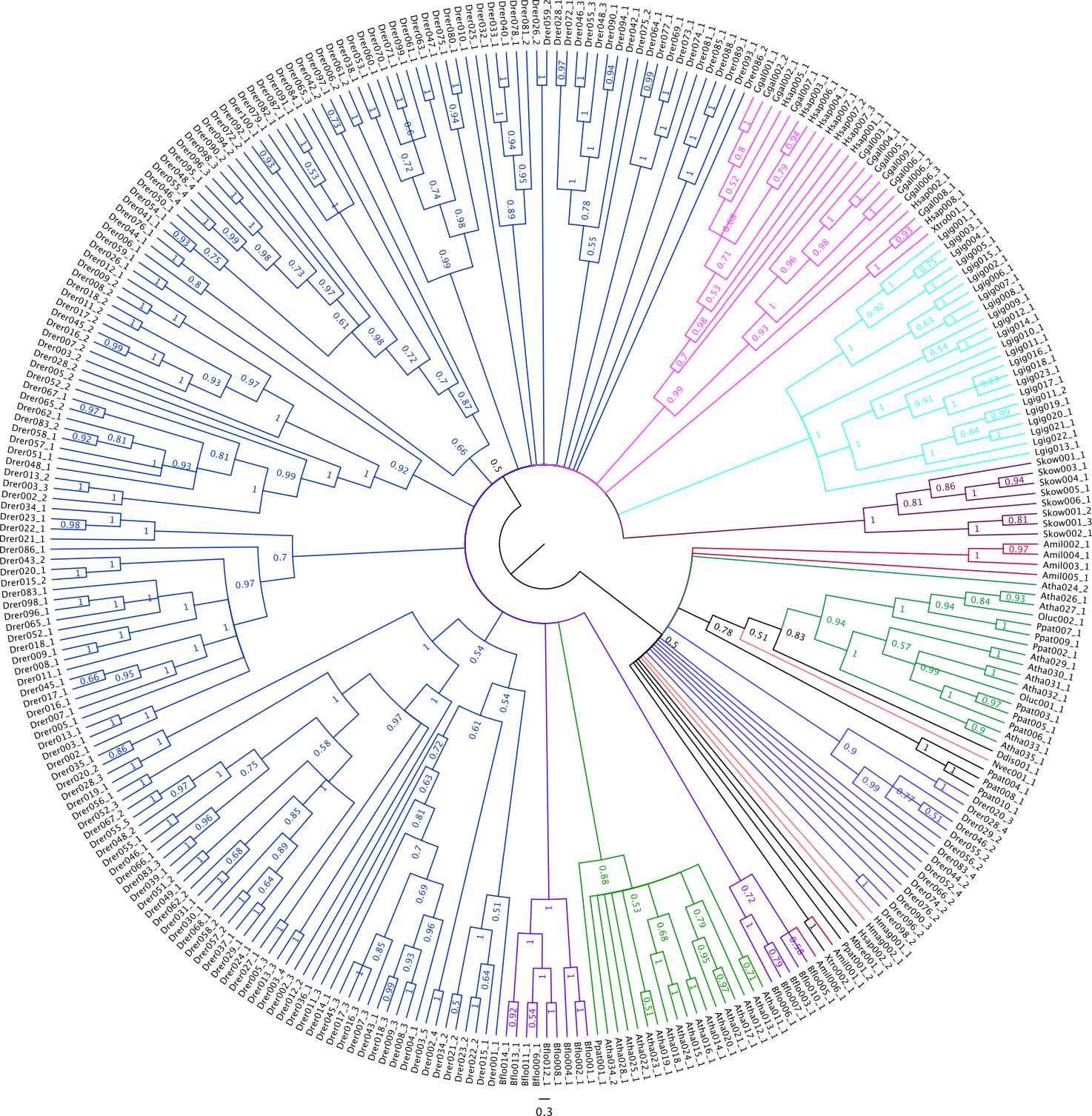

Supplement: Additional file 6 — Bayesian inference phylogenetic analysis of AIG1 domains. Posterior probability values indicated for all nodes. [file 1471-2164-14-400-S6.pdf]
