# Supplementary material for: The acute transcriptional response of the coral Acropora millepora to immune challenge: expression of GiMAP/IAN genes links the innate immune responses of corals with those of mammals and plants
Source: BMC Genomics. 2013 Jun 14;14:400. doi: 10.1186/1471-2164-14-400 (PMC3723955; doi:10.1186/1471-2164-14-400)
Supplement: Additional file 2 — Transcriptome clusters differentially regulated under Poly I:C challenge; up-regulated clusters are shown in green, down-regulation is indicated in red. BlastX comparisons were carried out against the adi_aug101220 Acropora digitifera predicted protein set, or v1.0 of the Nematostella vectensis protein predictions via the OIST and JGI genome browsers respectively, or against the NR database via NCBI using a cutoff of E-5. [file 1471-2164-14-400-S2.pdf]

Genes responding to Poly I:C

Cluster	Assignment	Fold change (Poly I:C)	Best non-cnidarian BlastP hit (Locus ID)	Organism	e value NR	Best N. vectensis BlastP hit (Locus ID)	e value Nv	Best A. digitifera BlastP hit (Gene model)	e value Ad
Cluster000112	Serine/threonine protein kinase / RING?	3.976	XP_001629791	Branchiostoma	0.00E+00	XP_001629791	0.00E+00	aug_v2a.07074	0.00E+00
Cluster005964	Neuroblast differentiation-associated protein AHNAK	3.933	XP_002807401	Callithrix	1.00E-16	XP_001625669	3.00E-13	aug_v2a.05975	0.00E+00
Cluster017126	Tumor necrosis factor receptor superfamily	3.647	EGD74812	Salpingoeca	5.00E-10	XP_001629793	1.00E-07	aug_v2a.14012	3.00E-143
Cluster022357	Collagen alpha-1(XII)	3.608	XP_002936246	Xenopus	4.00E-24	XP_001640936	2.00E-42	aug_v2a.05363	5.00E-123
Cluster000197	Lipophorin precursor	3.472	XP_002738323	Saccoglossus	3.00E-112	XP_001640190	1.00E-29	aug_v2a.01780	0.00E+00
Cluster010294	No hit	3.423	No significant hit			No hit		No hit	
Cluster000809	NFX1-type zinc finger	3.282	XP_002593922	Branchiostoma	0.00E+00	XP_001622790	0.00E+00	aug_v2a.21009	0.00E+00
Cluster010611	Sacs protein / sacsin	3.223	CAE45964	Homo	4.00E-59	XP_001636175	5.00E-67	aug_v2a.10290	9.00E-126
Cluster024126	Collagen, type alpha-1(XII)	3.158	EHH55386	Macaca	3.00E-05	XP_001640936	1.00E-13	aug_v2a.05363	6.00E-62
Cluster000008	Collagen, type alpha-1(XXIX)	3.074	XP_002734929	Saccoglossus	2.00E-61	XP_001632436	2.00E-58	aug_v2a.05363	0.00E+00
Cluster012102	Glucose-methanol-choline oxidoreductase / cholesterol oxidase	3.019	XP_002603265	Branchiostoma	2.00E-154	No hit		aug_v2a.18867	0.00E+00
Cluster009161	Collagen alpha-6(VI) chain	2.649	EKC40283	Crassostrea	5.00E-68	XP_001632943	1.00E-57	aug_v2a.05363	8.00E-178
Cluster001819	Glutamate synthase [NADPH]	2.431	XP_002612218	Branchiostoma	0.00E+00	XP_001630774	0.00E+00	aug_v2a.08445	0.00E+00
Cluster000000	Microtubule-actin crosslinking factor	2.361	XP_002606270	Branchiostoma	0.00E+00	XP_001629769	0.00E+00	aug_v2a.03009	0.00E+00
Cluster027576	Poly(Asp) protein	2.341				No hit		No hit	
Cluster000010	Viral A-type inclusion protein / kinesin	2.239	XP_001687712	Leishmania	3.00E-13	No hit		aug_v2a.18644	0.00E+00
Cluster014845	Aquaporin 4	0.432	AEI25531	Penaeus	2.00E-63	XP_001633187	5.00E-104	aug_v2a.21442	2.00E-51
Cluster024836	No hit	0.422	No significant hit			No hit		aug_v2a.18833	4.00E-49
Cluster009110	No hit	0.422	No significant hit			No hit		No hit	
Cluster011123	Glucose-methanol-choline oxidoreductase	0.418	XP_797018	Strongylocentrotus	6.00E-165	No hit		aug_v2a.13041	0.00E+00
Cluster025472	Similar to zonadhesin	0.413	XP_002735705	Saccoglossus	4.00E-09	XP_001623715	4.00E-39	aug_v2a.07532	7.00E-108
Cluster027673	No hit	0.385				No hit		No hit	
Cluster006973	Cytochrome P450; family 3	0.383	XP_002110410	Trichoplax	4.00E-113	XP_001625659	5.00E-131	aug_v2a.16872	0.00E+00
Cluster019644	Membrane-spanning 4-domains	0.377	XP_001923190	Danio	2.00E-04	No hit		aug_v2a.00537	5.00E-128
Cluster015370	No hit	0.375	No significant hit			No hit		No hit	

Cluster020716	No hit	0.367	No significant hit			No hit		aug_v2a.03590	2.00E-34
Cluster010905	Unique to Nematostella and Acropora	0.342	No significant hit			XP_001626516	2.00E-57	aug_v2a.06335	5.00E-150
Cluster002820	Hypothetical protein Esi_0067_0076	0.339	CBJ27377	Ectocarpus	7.00E-13	No hit		aug_v2a.10728	0.00E+00
Cluster020217	No hit	0.339	No significant hit			No hit		aug_v2a.17443	3.00E-30
Cluster009013	No hit	0.336	No significant hit			No hit		No hit	
Cluster026412	C009-F8 [Acropora millepora]	0.323	No significant hit			No hit		aug_v2a.21737	7.00E-121
Cluster017538	No hit	0.317	No significant hit			No hit		aug_v2a.00046	4.00E-42
Cluster023562	No hit	0.313	No significant hit			No hit		No hit	
Cluster018949	C005-B5 [Acropora millepora] 4e-48/ Soma ferritin	0.303	AF467696_1	Dermacentor	8.00E-14	XP_001627335	8.00E-17	aug_v2a.18796	4.00E-42
Cluster009706	Secreted frizzled-related protein 2	0.302	NP_001071964	Ciona	7.00E-21	XP_001638620	1.00E-22	aug_v2a.11469	0.00E+00
Cluster015600	No hit	0.296	No significant hit			No hit		No hit	
Cluster026469	TIR1 [Acropora millepora] 2e-61	0.295	XP_002586973	Branchiostoma	9.00E-11	XP_001641546	9.00E-13	aug_v2a.02686	2.00E-51
Cluster024059	Argonaute-like	0.292	XP_002737100	Saccoglossus	2.00E-117	XP_001630646	2.00E-127	aug_v2a.00902	0.00E+00
Cluster001145	Patched domain-containing protein 3	0.291	XP_002598387	Branchiostoma	2.00E-98	XP_001629035	0.00E+00	aug_v2a.12942	0.00E+00
Cluster015736	Hypothetical protein	0.288	XP_002740514	Saccoglossus	5.00E-18	XP_001627459	2.00E-48	aug_v2a.00569	1.00E-72
Cluster017128	Alpha crystallin hsps23 protein	0.282	XP_003113991	Caenorhabditis	6.00E-15	XP_001625684	7.00E-16	aug_v2a.15424	4.00E-69
Cluster011031	Fibrillar collagen, hypothetical	0.282	EKX49450	Guillardia	1.00E-18	XP_001639246	4.00E-52	aug_v2a.11457	5.00E-152
Cluster013013	No hit	0.277	No significant hit			No hit		aug_v2a.08576	1.00E-42
Cluster008529	Neuronal acetylcholine receptor subunit beta-3	0.275	XP_002819095	Pongo	1.00E-70	XP_001628236	7.00E-86	aug_v2a.11311	2.00E-69
Cluster015761	WD-40 repeat / TPR_2 / Fis family transcriptional regulator	0.271	YP_007109256	Geitlerinema	2.00E-25	XP_001634111	2.00E-35	aug_v2a.16679	2.00E-128
Cluster016915	Hemicentin / peroxidasin	0.269	XP_002405927	Ixodes	2.00E-21	XP_001624042	3.00E-19	aug_v2a.22439	5.00E-166
Cluster014162	No hit	0.263	No significant hit			No hit		aug_v2a.04638	8.00E-15
Cluster027019	Hypothetical protein	0.260	XP_002602912	Branchiostoma	3.00E-13	XP_001641728	5.00E-06	aug_v2a.02060	3.00E-77
Cluster021224	Hypothetical protein	0.259	EJY70955	Oxytricha	1.00E-05	No hit		aug_v2a.12582	8.00E-163
Cluster022120	No hit	0.257	No significant hit			No hit		No hit	
Cluster005198	No hit	0.252	No significant hit			No hit		aug_v2a.02663	0.00E+00
Cluster014577	Endonuclease domain-containing	0.239	XP_001345641	Danio	4.00E-11	No hit		aug_v2a.08450	0.00E+00
Cluster025912	Integrase / recombinase	0.233	XP_002736419	Saccoglossus	5.00E-20	XP_001639092	2.00E-18	aug_v2a.02878	7.00E-36
Cluster014878	Olfactomedin-like protein 2B	0.231	CAG01427	Tetraodon	2.00E-45	No hit		aug_v2a.22273	0.00E+00
Cluster004646	Matrix metalloproteinase	0.212	XP_002738586	Saccoglossus	9.00E-114	XP_001640669	8.00E-168	aug_v2a.02174	0.00E+00
Cluster011324	Olfactomedin-like protein	0.206	CAG01427	Tetraodon	4.00E-46	No hit		aug_v2a.22273	2.00E-146
Cluster010496	Conserved Hypothetical protein [Hydra + fungi]	0.194	EKC19238	Crassostrea	1.00E-25	No hit		aug_v2a.00773	3.00E-104

Cluster012254	2'-5'-oligoadenylate synthase 1G	0.173	XP_002704723	Bos	5.00E-16	XP_001635415	2.00E-20	aug_v2a.01178	1.00E-55
Cluster018924	Nucleotide-binding oligomerization domain containing	0.167	XP_002732748	Saccoglossus	1.00E-18	XP_001622189	4.00E-28	aug_v2a.11758	4.00E-120
Cluster011563	Senescence-associated protein [Mytilus]	0.157	AEM36070	Mytilus	3.00E-40	XP_001618678	8.00E-41	No hit	
Cluster013676	No hit	0.134	No significant hit			No hit		No hit	
Cluster018287	No hit	0.130	No significant hit			No hit		aug_v2a.02395	2.00E-145
Cluster015535	Hypothetical protein cnidarians and insects	0.129	XP_003396199	Bombus	3.00E-10	XP_001628368	3.00E-04	aug_v2a.24381	1.00E-12
Cluster020105	No hit	0.127	No significant hit			No hit		aug_v2a.00315	3.00E-58
Cluster005951	Peroxidase	0.113	XP_623940	Apis	4.00E-106	XP_001622220	3.00E-109	aug_v2a.24630	0.00E+00
Cluster021271	Cys and Gly-rich protein 1	0.112	XP_003893354	Papio	1.00E-20	XP_001629731	5.00E-16	aug_v2a.12783	5.00E-25
Cluster004281	No hit	0.078	No significant hit			No hit		aug_v2a.02060	4.00E-26
Cluster014665	Actin, cytoskeletal 2B	0.076	XP_001176242	Strongylocentrotus	0.00E+00	XP_001630583	0.00E+00	aug_v2a.05332	0.00E+00
Cluster018020	Sacsin / DnaJ-like protein subfamily C	0.075	XP_417138	Gallus	3.00E-36	XP_001624963	2.00E-42	aug_v2a.03342	3.00E-158
Cluster023450	Spliceosome RNA helicase	0.069	CAM25877	Homo	5.00E-173	XP_001635457	3.00E-175	aug_v2a.09757	1.00E-161
Cluster025642	No hit	0.066	No significant hit			No hit		aug_v2a.02568	2.00E-53
Cluster010227	Exonuclease 3-5 domain-like	0.053	XP_002597496	Branchiostoma	8.00E-105	XP_001639016	3.00E-143	aug_v2a.03677	0.00E+00
Cluster027039	No hit	0.053	No significant hit			No hit		aug_v2a.00732	2.00E-155
Cluster019507	Hypothetical protein	0.032	XP_003731402	Strongylocentrotus	3.00E-10	XP_001625301	1.00E-23	aug_v2a.16067	1.00E-41
Cluster026715	Hypothetical protein	0.032	EGD80721	Salpingoeca	7.00E-11	No hit		aug_v2a.18130	1.00E-18
Cluster006275	Hypothetical protein	0.000	XP_002937628	Xenopus	4.00E-18	No hit		aug_v2a.03714	3.00E-16
Cluster016383	Hypothetical protein	0.000	XP_002118488	Trichoplax	3.00E-70	XP_001629388	1.00E-46	aug_v2a.17310	2.00E-92
Cluster023841	Carbonic anhydrase	0.000	EKC20938	Crassostrea	9.00E-22	XP_001627923	3.00E-21	aug_v2a.19067	3.00E-51
Cluster004138	Pre-mRNA processing factor 6	0.000	JAA59769	Rhipicephalus	0.00E+00	XP_001625285	0.00E+00	aug_v2a.00906	4.00E-131
