# Supplementary material for: The acute transcriptional response of the coral Acropora millepora to immune challenge: expression of GiMAP/IAN genes links the innate immune responses of corals with those of mammals and plants
Source: BMC Genomics. 2013 Jun 14;14:400. doi: 10.1186/1471-2164-14-400 (PMC3723955; doi:10.1186/1471-2164-14-400)
Supplement: Additional file 3 — Transcriptome clusters differentially regulated under MDP challenge; up-regulated clusters are shown in green, down-regulation is indicated in red. BlastX comparisons were carried out against the adi_aug101220 Acropora digitifera predicted protein set, or v1.0 of the Nematostella vectensis protein predictions via the OIST and JGI genome browsers respectively, or against the NR database via NCBI using a cutoff of E-5. [file 1471-2164-14-400-S3.pdf]

Genes responding to MDP

Cluster	Assignment	Fold change (MDP)	Best non-cnidarian BlastP hit (Locus ID)	Organism	e value NR	Best N. vectensis BlastP hit (Locus ID)	e value Nv	Best A. digitifera BlastP hit (Gene model)	e value Ad
Cluster023274	Serine protease	Inf	NP_999239	Sus	1.00E-42	XP_001633098	7.00E-48	aug_v2a.11369	1.00E-43
Cluster009040	YD-repeat containing protein	251.961	ZP_04608751	Micromonospora	2.00E-07	No hit		aug_v2a.16950	2.00E-133
Cluster024980	GTPase IMAP family member	229.304	ACQ58961	Anoplopoma	2.00E-16	No hit		aug_v2a.23824	1.00E-83
Cluster012256	GTPase IMAP family member	115.484	XP_001914755	Equus	3.00E-11	No hit		aug_v2a.23822	4.00E-84
Cluster023012	No significant hits	60.515				No hit		No hit	
Cluster017267	C009-F8-like	59.731	No other hits	Acropora millepora	1.00E-14	No hit		aug_v2a.10491	4.00E-121
Cluster015015	GTPase IMAP family member	56.249	NP_001037788	Danio	1.00E-11	No hit		aug_v2a.23823	2.00E-101
Cluster013111	GTP-binding protein EsdC	32.288	XP_003349531	Sordaria	6.00E-94	No hit		No hit	
Cluster025107	Nucleoside phosphotylase	32.131	YP_003885815	Cyanothece	5.00E-09	No hit		aug_v2a.01084	2.00E-36
Cluster025657	C009-F8	18.963	No other hits	Acropora millepora	2.00E-52	No hit		aug_v2a.14070	3.00E-76
Cluster026965	Non-canonical histone H3.3	14.305	XP_002113772	Trichoplax	1.00E-91	XP_001623766	1.00E-91	aug_v2a.13799	1.00E-19
Cluster002193	Formate acetyltransferase	13.799	XP_002596398	Branchiostoma	0.00E+00	No hit		aug_v2a.18719	0.00E+00
Cluster002489	CDH1-D	12.647	AAL31950	Gallus	1.00E-30	No hit		No hit	
Cluster013871	No significant hits	10.926				No hit		No hit	
Cluster020990m	Conserved hypothetical protein	9.770	CAJ30045	Magnetospirillum	1.00E-56	No hit		No hit	
Cluster022345	Senescence-associated protein	9.321	XP_003542256	Glycine max	3.00E-27	XP_001618890	2.00E-17	No hit	
Cluster006412	Senescence-associated protein	9.076	XP_001900327	Brugia	7.00E-58	XP_001618678	5.00E-33	No hit	
Cluster019383	Histone H2B	8.847	CBM82447	Rhabdopleura	2.00E-59	XP_001617661	2.00E-54	aug_v2a.03254	9.00E-53
Cluster023174	Glycosyl transferase family	8.815	XP_003729243	Strongylocentrotus	9.00E-94	XP_001637936	1.00E-110	aug_v2a.03764	7.00E-99
Cluster015042	igLON family member / lachesin	8.482	XP_002938252	Xenopus	6.00E-21	XP_001625597	2.00E-17	aug_v2a.16744	7.00E-98
Cluster013830	Hypothetical protein	8.150	XP_002723895	Oryctolagus	9.00E-21	No hit		No hit	
Cluster002963	rRNA promoter binding protein	8.038	EFN65035	Camponotus	4.00E-36	XP_001624693	1.00E-10	No hit	
Cluster018413	No significant hits	6.865				No hit		aug_v2a.06165	3.00E-45
Cluster007853p	Hypothetical protein	6.523	XP_003614392	Medicago	2.00E-79	XP_001618678	2.00E-30	No hit	
Cluster013535	FGF receptor	6.150	NP_001037558	Bombyx	1.00E-05	XP_001637292	4.00E-06	aug_v2a.17718	7.00E-131
Cluster023536p	Hypothetical protein	5.934	XP_003542256	Medicago	4.00E-30	XP_001618890	2.00E-18	No hit	
Cluster008124m	CDH1-D	5.824	AAL31950	Gallus	2.00E-20	No hit		No hit	
Cluster012844	No significant hits	5.754				No hit		aug_v2a.03567	1.00E-71
Cluster017835	Hypothetical protein	5.561	XP_710281	Candida	4.00E-25	No hit		No hit	
Cluster018298	Cephalosporin hydroxylase	5.398	ZP_08430922	Moorea	9.00E-50	XP_001635762	2.00E-58	aug_v2a.16414	2.00E-154
Cluster004461p	Pol-like protein/transposon TX1	5.391	XP_002740396	Saccoglossus	9.00E-90	XP_001631247	6.00E-07	aug_v2a.22112	0.00E+00

Cluster015137	Cathepsin L-like cysteine proteinase	5.039	AEO32878	Amblyomma	6.00E-139	XP_001628790	1.00E-167	aug_v2a.13010	3.00E-140
Cluster019424	Uncharacterised plant protein	4.781	XP_002463101	Sorghum	2.00E-07	No hit		aug_v2a.12463	4.00E-27
Cluster017997	No significant hits	4.585				No hit		No hit	
Cluster012104	Choloylglycine hydrolase pA79-1	4.526	YP_002363808	Methylocella	2.00E-63	No hit		aug_v2a.03137	0.00E+00
Cluster026172m	Cytochrome P450 monooxygenas	3.810	GAA96382	Mixia	7.00E-34	No hit		No hit	
Cluster014083	No significant hits	0.255				No hit		No hit	
Cluster009725	Guanylate-binding protein, N-terminal domain	0.246	XP_002610120	Branchiostoma	8.00E-22	XP_001638595	2.00E-88	aug_v2a.12397	8.00E-115
Cluster008297	Notch/pentraxin-like unknown	0.246	XP_002599260	Branchiostoma	6.00E-51	XP_001631432	6.00E-16	aug_v2a.08407	0.00E+00
Cluster000397	EGF-containing unknown	0.242	XP_003385535	Amphimedon	4.00E-132	XP_001639249	4.00E-56	aug_v2a.21085	0.00E+00
Cluster001272	SCO-spondin / hemicentrin	0.224	EKC21775	Crassostrea	0.00E+00	XP_001639686	5.00E-96	aug_v2a.12523	0.00E+00
Cluster015890	Hypothetical polydom like	0.213	XP_002604604	Branchiostoma	4.00E-66	XP_001621820	2.00E-56	aug_v2a.08407	3.00E-116
Cluster006582	Uncharacterized protein	0.197	XP_003728220	Strongylocentrotus	8.00E-35	XP_001624782	3.00E-08	aug_v2a.20821	0.00E+00
Cluster008461	Guanylate-binding protein, N-terminal domain	0.165	XP_002610120	Branchiostoma	3.00E-22	XP_001638595	3.00E-100	aug_v2a.02745	2.00E-153
Cluster025698	TIR1 [Acropora millepora]	0.161	ABK78770	No other close hits	0.00E+00	No hit		aug_v2a.12782	3.00E-60
Cluster019759	Neurotrimin	0.153	BAH11509	Homo	2.00E-22	XP_001624042	7.00E-22	aug_v2a.22439	4.00E-152
Cluster017699	Hemicentin-like	0.153	AAI57653	Xenopus	3.00E-27	XP_001622494	1.00E-46	aug_v2a.17555	7.00E-163
Cluster008363	No significant hits	0.055				No hit		aug_v2a.03587	6.00E-75
Cluster018454	Conserved novel protein	0.049	CAK10784	Danio	2.00E-21	XP_001621890	2.00E-35	aug_v2a.02341	1.00E-74
Cluster020755	Cysteine protease	0.027	XP_003643791	Gallus	1.00E-33	XP_001636228	5.00E-56	aug_v2a.15295	3.00E-57
Cluster006562	PiggyBac Transposase (Acropora ACT79639)	0.027	JAA62622	Rhipicephalus	9.00E-127	XP_001630863	1.00E-08	aug_v2a.15307	0.00E+00
Cluster000805p	Kyphoscoliosis peptidase	0.014	EKC30166	Crassostrea	2.00E-43	XP_001632100	2.00E-158	aug_v2a.09652	0.00E+00
