# Supplementary material for: The acute transcriptional response of the coral Acropora millepora to immune challenge: expression of GiMAP/IAN genes links the innate immune responses of corals with those of mammals and plants
Source: BMC Genomics. 2013 Jun 14;14:400. doi: 10.1186/1471-2164-14-400 (PMC3723955; doi:10.1186/1471-2164-14-400)
Supplement: Additional file 4 — Domain matches for A. millepora and H. magnipapillata. [file 1471-2164-14-400-S4.pdf]

Domain search data (e values)

	Assignment	AIG1 cl09099	AIG1 pfam04548	Toc34_like cd01853	Era_like cd00880	MMR_HSR1 pfam01926	Ras-like cd00882	HintN smart00306	Hint cd00081	Hint cl15780	Hint pfam01079	RING cd00162
**Acropora**												
Cluster012256	**GiMAP1**	4.40E-14	9.57E-14	5.85E-05	6.70E-05	7.43E-05	Not signif					
Cluster024980	**GiMAP2**	4.53E-21	1.53E-19	1.98E-10	0.0001423	0.00131687	4.19E-07					
Cluster015015	**GiMAP3**	1.13E-13	5.58E-13	0.00011174	1.44E-06	0.00308809	7.68E-06					
Cluster005148		5.29E-39	1.79E-35	7.31E-07	1.21E-06	2.05E-08	5.34E-06	1.24E-13	3.34E-13	3.34E-13	1.10E-42	
Cluster013591		8.17E-10	2.90E-06	Not signif	Not signif	Not signif	Not signif	2.26E-13	1.49E-12	1.49E-12	6.27E-42	
Cluster005603		5.15E-33	1.53E-30	1.09E-06	0.0002797	2.09E-06	3.50E-05	1.31E-13	6.79E-13	6.79E-13	1.04E-41	
Cluster008706		1.60E-35	8.64E-33	9.77E-08	1.27E-06	1.10E-07	2.53E-06	1.05E-12	2.71E-12	2.71E-12	6.96E-39	
Cluster019598		2.17E-35	2.82E-32	3.42E-07	1.32E-06	3.01E-09	3.40E-06					
**Hydra**												
XP_002159305		1.02E-06	Not signif	Not signif	Not signif	Not signif	Not signif					1.03E-08
XP_002168601		7.52E-07	2.91E-11	0.00058904	7.73E-05	1.01E-08	Not signif					

nb Cluster08706 may be an allele or splice variant of Cluster005148
Cut off: 0.01
CD search against CDD v3.08
