# Supplementary material for: The acute transcriptional response of the coral Acropora millepora to immune challenge: expression of GiMAP/IAN genes links the innate immune responses of corals with those of mammals and plants
Source: BMC Genomics. 2013 Jun 14;14:400. doi: 10.1186/1471-2164-14-400 (PMC3723955; doi:10.1186/1471-2164-14-400)
Supplement: Additional file 8 — Sequence alignments used in the phylogenetic analyses. [file 1471-2164-14-400-S8.docx]

CLUSTAL 2.1 multiple sequence alignment

Amil001_1 KVMMVVGSTGSGKTTTVNAMINHKDFRSNQAAETIGN--QAHSQTMEGFKLTIVDTPGFG

Amil002_1 RTVLVVGRTGAGKSTVANKILGRB---BBNVASSVTSEVEAMCSLSNRYKFTVIDTLGVF

Amil003_1 RNIIILGRTGTGKKTIANKLLGDERFQVDSAMTSVTREIR---SMRNHYNIKIVDTLGPY

Amil004_1 RNVVVVGKPSEGKSSVANKVLGLPKFKVTNTARRVASDVEARCSTSRRYIFKVIDTVGIL

Amil005_1 LNVALMGITGSGKSALIKTIFECLGFNSSAVTQDTAKEGTRKLDSLPGSQVTFYDTAGFF

Amil006_1 LTVVLIGKTGNGKSSAGNQILGQKLFAVSDSATSETMFCTKQTR-TDEREITVIDTPGVL

Atha011_1 ENIVLVGRTGNGKSATGNSLIGKKVFASKAHASGVTMKCQTHGVVKDGHKINVIDTPGLF

Atha012_1 KNVVLVGRTGNGKSATGNSIIGRKVFESKYQAVGVTTRCKTFRAVPDGPIINVIDTPGLF

Atha013_1 ENIVLVGRTGNGKSATGNSIVRSKVFKSKTKSSGVTMECHAVKAVPEGPILNVIDTPGLF

Atha014_1 RTLVLVGRTGNGKSATGNSILGRKAFRSRARTVGVTSTCESQRVVEDGDIINVVDTPGLF

Atha015_1 RTLLLVGRSGNGKSATGNSILGRKAFKSKGRASGVTTACELQSSTPNGQIINVIDTPGLF

Atha016_1 RTLVLVGRTGNGKSATGNSILGRKAFRSRARTVGVTSTCESQRVVEDGDIINVVDTPGLF

Atha017_1 KNIVLVGRTGNGKSATGNSLIGKQVFRSETRATGVTMKCETCVAVPCGTGINVIDTPGLF

Atha018_1 RTLVLLGRTGNGKSATGNSILGKTMFQSKARGKFITKECKLHKSKPNGLTINVIDTPGLF

Atha019_1 KNIVLVGRTGNGKSSTGNTLLGTKQFKSKNQAKGVTMICEMYRAAQDGPIINVIDTPGLC

Atha020_1 KNIVLVGRTGNGKSATGNSLIGKDVFVSEAKATGVTKTCQTYKAVPGGSRINVIDTPGLF

Atha021_1 RNIVLVGPTGNGKSSTGNSLIGKEVFILE------TVECKTCKAKLDGQIINVIDTPGLF

Atha022_1 KNIVLVGRSVNGICTTGNNILGQNKFGS----EGAFMHCQMYSTTPDGQMINVIKTPGMF

Atha023_1 TNLLLLGRSENGKSSTGNTIIGEKYFEVNLFGRDMDQRCKMFRALEDGPIINVIDTPGLL

Atha024_1 RTLVLIGCSGNGKSATGNSILRSEAFKSKGQAAAVTKECELKSTKPNGQIINVIDTPGLF

Atha024_2 KIRAMKSNTKQEMSNW-----------KEEEVSPLAKKV-----------EKVIETTSLL

Atha025_1 ------------------------MFTSELQAGGVTMECVMYRTAKDGPIINVIDTPGLF

Atha026_1 LTVLVMGKGGVGKSSTVNSVIGEKAAAVST-FQSEGLRPTLVSRTRSGFTLNIIDTPGLI

Atha027_1 MTVLVLGKGGVGKSSTVNSLIGEQVVRVSP-FQAEGLRPVMVSRTMGGFTINIIDTPGLV

Atha028_1 QTIDLVGTTGSGETATANNIQGKKVVQSGTHATVVTMECQTYKVFTPCPINNMIDTPGLL

Atha029_1 LRILVLGKTGVGKSATINSIFGQPKSETDA-FRPGTDRIEEVMGTVSGVKVTFIDTPGFH

Atha030_1 LRILVLGKTGVGKSATINSIFGQPKSETDA-FRPGTDRIEEVMGTVSGVKVTFIDTPGFH

Atha031_1 CTIMVLGKSGVGKSATINSIFDELKISTDA-FQVGTKKVQDIEGFVQGIKVRVIDTPGLL

Atha032_1 CTIMVLGKSGVGKSATINSIFDEVKFCTDA-FQMGTKRVQDVEGLVQGIKVRVIDTPGLL

Atha033_1 INVLVIGKTGVGKSATVNSIFGETKSAVGA-FGVTTNSANYVVGNVGGIQISILDTPGLL

Atha034_2 TSIVLVGRNGNGKSFTGNTLLGEKLFISKADAGGVTMEEKEKERE--------IESKSLA

Atha035_1 LNILVLGKAGVGKSATINSILGNQIASIDA-FGLSTTSVREISGTVNGVKITFIDTPGLK

Bflo001_1 LRIALIGMTGAGKSSTANTIVGEQKFEAACTASSETGRCSYGKREKDDREVSVVDTPGVW

Bflo002_1 LRIALVGVTGAGKSSTANTIVGEKKFKASSGASSKTKGCSYEKRKKGDREIAVVDTPGVW

Bflo003_1 YNIVLLGRTGSGKSATGNSIVGDRVFEESDMGGSQTKNCDNAKACINGYILNVIDTPGFA

Bflo004_1 LRMAVIGKTGVGKSSTANTIVGSKEFRVTCSASSETTKSAYTRRQKTDRKIAVVDTPGIC

Bflo005_1 LTVVMVGKTGHGKSCLGNSILGRKAFTDSPMGSSTTKTSMKESATINGIRFHVVDTPGVM

Bflo006_1 --IVLLGRTGNGKSATGNSIVGNNVFNVSKRWGSETTTCDNAKACIDGYILNVIDTPGFA

Bflo007_1 --IVLLGKTGSGKSATGNSIVGDRVFEESDMGGSQTKTCDNAKACIDKYILNVIDTPGFA

Bflo008_1 LGIVLVGKTGVGKSHTGNNITGTKKFRVSDKAKSETRVCKQHIRQKD-RQITVLDTPGVF

Bflo009_1 LHMVLIGKTGVGKSSTGNSIIGEDVFKVATVAATVTTKCNFHIRTKDGSKLAILDTPGLF

Bflo010_1 --LLLFGKTGSGRSATGNSILGSKAFAASPML-HATTTCDIKTCERDGRILRVVDTPDIT

Bflo011_1 LNITIIGKTGVGKSHTGNTIIGKNTFKVGDIASSITTVCASGDREKEDREIEVLDTPGVF

Bflo012_1 LGIVLVGKTGVGKSHTGNNITGK-EYKVSDKA-----RCEQHIRQKD-RQITVLDTPGVF

Bflo013_1 LRIALIGKTGAGKSSTANSILGYAASAVSCGLSSETKHCLFFTRDKGDRKISVVDTPGIL

Bflo014_1 LRLALMGKTGVGKSSTGNSIIGCEKFTFSCSAASETPDCPYHRREQP-RKVAVLDSPGVM

Ddis001_1 CNVLLLGRTGVGKSSTLNTVFGIDIPVHSS--ESCTQDPFTYSRVVNGFKLNIIDTPGFL

Drer001_1 IRIVLVGKTGVGKSATGNTILGEKAFNSEARATSITKECSRESRMIDRKQVSIVDTPGLY

Drer002_1 LRIVLIGKNASENSRVENFI-GPAVFD-----SGASFYVKQTRFSGQERNIRVFHFPHLL

Drer002_2 LNLVVCGSNRRLKSSMSELFLR-----ESRRGSVVGSEFTRTDVDLHGRLISLMEFPTLI

Drer002_3 VRIVLLGKTGVGKSATGNTILGRKAFTSDISQSSVTKECQKVTVQVNSQNITVIDTPGLF

Drer002_4 LRILLFGKTGNGKSATGNTILRKNYFHAETSSSLVTRVCQKEVVKVDGKTVSIIDTPGLF

Drer003_1 LRIVLIGKNGSENSRVENVI-GAAVYD-----SGASSHVRQTGINGQERNIRVFNMPNLL

Drer003_2 LNVVLCGNNTTLKSSVSKVFRGTKKIKGHILQKDRSNECVKKGGKFDGREISVVKLPAL-

Drer003_3 LNLVLCGSNRKLKSSLSELFLS-----ESTRGSVVSSEFTKRDLDLHGRLINVMEFPALI

Drer003_4 LRIVLLGKTGVGKSATGNTILGRKEFKSDISQSSVTNVCQKQTAEINGRHITVIDTPGLF

Drer003_5 LRIVLFGKKGTGKSATGNTILGNEEFSTAAGSQLMTKNCQKGVGEAEGKRVSIVDTPGLL

Drer004_1 LRIVLIGRTGNGKSATGNTILGRNEFLSQLSMDSVTTVCEKGVGEVDGRSVAVVDTPGLF

Drer005_1 LRIVLLGKNTTENSSVGNFILGRSAFESEAPSADVELHIEREKGKLQDREVTVVNDSQLL

Drer005_2 LNVVLCGRDRGLKSSISRLMLD-----QRDKESELSSECVKLDGEVDGRLITLVELPAL-

Drer005_3 LRIVLLGKTGVGKSATGNTILGREAFKEDVSQESVTKECQRQTTDVDGRSITVIDTPGLF

Drer006_1 VRIVLLGVCGAGKSSMGNAILGEDVFK-----EGRTRESEIQRGRVEGRNISIIDTPGFF

Drer006_2 LRIVLVGKTGVGKSATGNTILGEDMFKEELSAESVSEKCLKQEKNIDGRTISVIDTPGLC

Drer007_1 IRIVLLGKSASENSRVRNLILGTDPCENEDPAACLRHNVTQIAGTVKDRHVTVINTLHLL

Drer007_2 LNLVLCGSNGSLKVSVSKLLRGK-KFKSTS--RQASSEICVRKEKIHGRQVSLLELPAL-

Drer007_3 LRIVVIGRTGSGKSATGNTILGREEFCSQLRADSVTNVCEKGVGEVDGQSVAVIDTPGLF

Drer008_1 LRILLLGKSVSENSRVGNLILGRSAFDSEAPPDVV----ERVGGRLIDRHVTLINSPQLL

Drer008_2 --------------SVSKLL-------SHSS-QTDSSGVCVKKQKIHDRQINIVNLPAL-

Drer008_3 LRIVLIGRTGSGKSATGNTILGRKEFVSKARSDSVTTVCEKGVCEVDGRSVAVVDTPGLF

Drer009_1 LRILLLGKSVSENSRVGNLILGRSAFDSEAPPDVV----ERVGGRLIDRHVTLINSPQLL

Drer009_2 --------------SVSKLL-------SHSS-QTDSSGVCVKKQKIHDRQINIVNLPAL-

Drer009_3 LRIVLIGRTGSGKSATGNTILGRKEFVSKARSDSVTTVCEKGVCEVDGRSVAVVDTPGLF

Drer010_1 LRIVMIGKTGVGKSAVGNTILNREVFESKPSANSITQSC-RKASVYDTREIYVIDTPGIL

Drer011_1 M--------------------------------------------VNGIHVTVITTLYLL

Drer011_2 LNLVLCGGNGLLKVSVSKLLRGK-PISTSHQ-RSSSEDFYKKEEKIDGRQVSLLELPAL-

Drer011_3 LRIVLIGRTGSGKSATGNTILGRNEFHSQTSADSVTTVCKKGVGEVDGRSVAVVDTPGLF

Drer012_1 QILVLCGSDAKLKNSISNLIRG-KSLTHSQPRNQNEEESLRTDVDLYGRQISLVNLPAL-

Drer012_2 LRIVLIGRTGSGKSATGNTILGREEFYSRMSTNSVTTVCKKGVGEVDGRSVAVVDTPGLF

Drer013_1 LRIVLIGKNGSENSRVENVI-GAAVYD-----SGASSHVRQTGINGQERNIRVFNMPNLL

Drer013_2 LNLVLCGSNRKLKSSLSELFLS-----ESTRGSVVSSEFTKRDLDLHGRLINVMEFPALI

Drer013_3 LRIVLLGKTGVGKSATGNTILGRKEFKSDISQSSVTNVCQKQTAEINGRHITVIDTPGLF

Drer014_1 LRIVLIGRTGNGKSATGNTILGKEEFCSQSNTDSVTTVCEKRVGEVDGRSVAVVDTPGLF

Drer015_1 VRIVLIGKTGVGKSATGNTILGHSAFESRARMTSTTKVCQRESGIACGRAVTVVDTPGLF

Drer015_2 LKIFLLGTSMSENNRVGNLILGKNVFVKKTPSSD-----EKHSGRVEDRNITVIIS-HLL

Drer016_1 ----------------------------EDLAALTRHNVTQIAGTVEDRHVTIINTLHLL

Drer016_2 LNVVLCGSNGSLKVSVSKLLRGK-KFKSTS--RQASSEIRVKKEKIHGRQVSLLELPAL-

Drer016_3 LRIVLIGRTGSGKSATGNTILGREEFCSQLRPDSVTNVCEKGVGEVDGRSVAVVDTPGLF

Drer017_1 ----------------------------EDLAALTRHNVTQIAGTVEDRHVMVINTLHLL

Drer017_2 LNLVLCGSNGSLKVSVSKLLRGK-KFKSTS--RQASSEIRVKKEKIHGRQVSLLELPAL-

Drer017_3 LRIVLIGRTGSGKSATGNTILGREEFCSQLRPDSVTNVCEKGVGEVDGRSVAVVDTPGLF

Drer018_1 LRILLLGKSVSENSRVGNLILGRSAFDSEAPPDVV----ERVGGSLKDRHVTLINSPQLL

Drer018_2 LNLVLCGSDGSLKVSASKLFRGK-PIATSHQ-RSSSEDFFKKEEKIHGHQVSLLELPAL-

Drer018_3 LRIVLIGRTGNGKSATGNTILGREEFLSQASMDSVTTVCEKEVCEVDGRSVAVVDTPGLF

Drer019_1 LRIVLLGKTGVGKSASANTILRRKSFQSVLTSQSVTKECQKETAEFSREHISVIDTPGLF

Drer020_1 LRIALLGTSMSENSRVGNLILGKGVFGKKTPSTD-----EEHSGRVGDRNITVISTD-LL

Drer020_2 LRIVVMGKTGVGKSASANTILRREAFKSVLNSQSVTKECQKETAEFSRRCITVIDTPGLF

Drer020_3 ------------------------------------VEKNIREEQKR-----------IL

Drer021_1 LRIVLLGSCRDVKASCGNTILGLQKFSESQST----IDFEWHAGLVLEQHLMIINTPGLL

Drer021_2 VRIVLIGKTGVGKSATGNTILGRNIFQSRPSMTCITKVCQRESGIACGRAVTVVDTPGLF

Drer022_1 LRIVLLGSDAAVKASCGNTIFGRQVFSESPPS---PHLFERHDGMVLKRRLVIINTPDLF

Drer022_2 VRIVLVGKTGVGKSATGNTILGRSAFESRARMTSVTKMCQRESGIACGRPVTVVDTPGLF

Drer023_1 LRIVLLGSEAAVKASCGNTIFGRQVFSESQPS---PHLFERHDGMVLKRRLVIINTPDLF

Drer023_2 VRIVLVGKTGVGKSATGNTILGRSAFESRARMTSITKMCQRESGIACGRPVTVVDTPGLF

Drer024_1 LRIVLQGKTGVGKSSTGNTILGRDAFPADLYEESVTKESQRKSSEINGRRITVIDTPGLF

Drer025_1 LRIVLIGKTGVGKSATGNTILGQEVFESAFLASSVTRKCEKKFGVVNGRRISIINTPGVF

Drer026_1 MRILLVGRKGSGKSSSGNTILGNKKFKVYKQNKKHESEVCKSDTKIRGMQVDVLDCPDLL

Drer026_2 VRLVLLGKTGVGKSATANTIIGKNRFKSTSSSRSQTKLCQTETRLRSSKQISVIDTPGLY

Drer027_1 LRIVLLGKTRVGKSATGNTILGREAFISDVSQESITKECQRETAQVNGRSITVIDTPGLF

Drer028_1 ----------------------------------------MGSGTVDGFSVTVYDTPGFF

Drer028_2 LH---------------------------------QKECVSKAVDLHGRLINVLELPAL-

Drer028_3 LRIVLLGKTGVGKSASANIILRKTAFKSALASKSVTRECQKDRAEFSRGRITVIDTPGLF

Drer028_4 -------------------------------------EKNIREEQKR-----------IL

Drer029_1 LRIVLLGKTGVGKSSTGNTILGRDAFKAGASTESVTEKSQRETSEINGRRITVIDTPGLF

Drer029_2 --FREMER-------------------------------EKQQQQMK-----------IL

Drer030_1 LRIVLLGKTGVGKSSTGNTVLEKEAFKAGISEESVTKETQRESCEINGRRITVIDTPGLF

Drer031_1 LRIVLLGKTGVGKSSTGNTILEKEAFKAGISEESVTKETQRESCEVNGRHITVIDTPGLF

Drer032_1 LRIILVGVTGAGRSASGNTILGKKVFQSEISSSSVTKRCETSNAIVHGRNISVVDTPGLI

Drer033_1 LRILLVGKTGVGKSATGNTIIGQDVFKSEISSSSVTGHCEKFHTVINGRKVSVIDSPGLF

Drer034_1 LRIVLLGSEAAVKASCGNTIFGRQVFSESPPS---PHLFERHDGMVLNRRLVIINTPDLF

Drer034_2 VRIVLIGKTGVGKSATGNTILGCRSFESRASMTCITKVCQRESGIACGRAVTVVDTPGLF

Drer035_1 RRIVLLGKTGVGKSASANTILRRKSFKSALTSQSVTKECQKDTTEFNTRRITVIDTPGLF

Drer036_1 LRIVLIGRTGSGKSTTGNTILGREEFPSQLNTDSVMNVCKKGVGEVDGQSVAVVDTPGLF

Drer037_1 LRIVLLGKTGVGKSSTGNTILGREAFKAGASIESVTEKSQRETSKIKGRRITVIDTPGLF

Drer038_1 LSIVLLGKTGSGKSSAGNTILGQKKFKSKASVVSVTKTCERGEAEINGKKISVIDTPGLL

Drer039_1 LRIVLLGKTGVGKSSTGNTILGRKAFKAEDYFESVTKQSQRETREVNGRRITVIDTPGLF

Drer040_1 LRILLVGKTGVGKSATGNTIIGQDVFKSEISSSSVTGHCEKFHTVINGRKVSVIDSPGLF

Drer041_1 MRIVLVGKTGVGKSAAGNTILGREQFKSVMKMNTITTKSLKTDATVSGRSVSVVDTPGLF

Drer042_1 --------------------------------------------------------PE--

Drer042_2 RRLILLGKTGVGKSATGNTILGINAFKSEQNFNSVTKQSEKLSSVVAGRDVSVIDTPGFF

Drer043_1 LRIVLIGRTGNGKSATGNTILGREEFLSQASMDSVTTVCEKEVCEVDGRSVAVVDTPGLF

Drer043_2 LRIVLLGKSIAENRRVVNLILNKEAFERKASSSG-----VEFSERVEGRNITVISTSQLL

Drer044_1 LRLVLLGKTGAGKSATGNTILGEKRFNDDLSMSSVTKECQRENTSTEGRNLLLVDTPGFF

Drer044_2 IEERVQKMEEVRRMADNRWA----AFT-----ASLNKERQKTRRRRK-----------CI

Drer045_1 ----------------------------EDLAALTRHNVTQIAGTVEDRHVMVINTLHLL

Drer045_2 LNVVLCGSNGSLKVSVSKLLRGK-KFKSTS--RQASSEIRVKKEKIHGRQVSLLELPAL-

Drer045_3 LRIVLIGRTGSGKSATGNTILGREEFCSQLRPDSVTNVCEKGVGEVDGRSVAVVDTPGLF

Drer046_1 VRIVLLGKTGVGKSATGNTIIGRAKFTAETSHQSVTKESQRETCEINGRQVTVIDTPGVF

Drer046_2 KMFREMER-------------------------------EKQEQQMK-----------IL

Drer046_3 LNVVLLGKRGAGKSASGNTILGRQVFISKKSARPVTRDVNVESGSFCELPVTVYDTPGLF

Drer046_4 RRIVLVGKSGVGKSAAGNTILGQKEFRSVRRMSSVTCKSSAAQTTVSGRSVSVVDTPPLF

Drer047_1 LRIVLLGKTGSGKSATGNTILDRKAFEVGEFIKSKSKQCEKKEGEFGGRTITIIDTPGLF

Drer048_1 --------DGSLKSSISELILQ-----HTHR----RSESVRTDV------IHVLELPALF

Drer048_2 VRIVLLGKTGVGKSTTGNTILGRKAFTAETSHLSVTKESQRETSEINGRQVTVVDTPGVF

Drer048_3 LNVVLLGKRGAGKSASGNTILGRQAFISKKSVRPVTQDVTVESGSFCELPVTVYDTPGLF

Drer048_4 RRIVLVGISGVGKSAAGNTILGQKEFTSVMSTNSVTRKCSAAQATVSGRSVSVVDTPGLF

Drer049_1 LRIVLLGKTGVGKSSTGNTILGRDAFAADISQESVTVTSQKESSEINGRLITVIDTPGLF

Drer050_1 LRIVLLGKTGVGKSAVGNTILGQEEFRSVSRMSSVTSECSAAQATVSGRSVSVVDTPGLF

Drer051_1 -NMVVCGSNSSLKSSISELILQ-----HTHR----RSESVRTDVDLHGRLINVLELPALF

Drer051_2 LRIVLLGKTGVGKSSTGNTILGRDAFAADISQESVTVTSQKESSEINGRLITVIDTPGLF

Drer052_1 LRIVLLGKSVSENSEVGNFILGRSAFDSEAPPGVV----ERVGGRLRDRHVTLINSPQLL

Drer052_2 LNVVVCGSNGSLKSSISELIPQ-----HTIR----RSGSVSTDVDLYGHQINVLELPALF

Drer052_3 LRIVLLGKSGVGKSATGNTILERYVFKAETSQESVTQESQSETREINGRHITVIDTPGLF

Drer052_4 ---------------------------------------KKEKRE-------------LW

Drer053_1 LRIVLLGKTGSGKSSAGNTILGQQLFTNDASLESVTNTCERGEAMIDGKKISVIDTPGRF

Drer054_1 LRIVLVGKTGVGKSAVGNTILGQKEFRSEMSSHSVTSKCSTAQATVSGRSVSVVDTPGFF

Drer055_1 VRIVLLGKTGVGKSATGNTIIGRAKFTAETSHQSVTKESQRETCEINGRQVTVIDTPGVF

Drer055_2 ----EMER-------------------------------EKQEQQMK-----------IL

Drer055_3 LNVVLLGKRGAGKSASGNTILGRQVFISKKSARPVTRDVNVESGSFCELPVTVYDTPGLF

Drer055_4 RRIVLVGKSGVGKSAAGNTILGQKEFRSVRRMSSVTCKSSAAQTTVSGRSVSVVDTPPLF

Drer055_5 LRIVLLGKTGVGKSTTGNTIIGRKAFTAETSHQPVTKESQRESCEINGRQVTVVDTPGVF

Drer056_1 LRIVLLGKTGVGKSATGNTILGRILFKASLSQESVTKESQSETREINGRHITVIDTPGLF

Drer056_2 -MFREMER-------------------------------KKQEQQMK-----------IL

Drer057_1 LNVLVCGSDGSLKSSISELILQ-----HTHR----RSESVRTDVDLHGRLINVLELPALF

Drer057_2 LRIILLGKTGVGKSSTGNTILGREAFKAGASQESVTETSQRESSEIKGRRITVIDTPGLF

Drer058_1 LNVLVCGSDGSLKSSISELILQ-----HTHR----RSESVRTDVDLHGRLINVLELPALF

Drer058_2 LRIILLGKTGVGKSSTGNTILGRNAFKAGASQESVTETSQRESSEINGRRITVIDTPGLF

Drer059_1 MRILLVGRKGSGKSSSGNTILRKKKFKVYKQNKKHESKLCNAVTEIRGTQVDVLDCPDLL

Drer059_2 VRLVLLGKTGVGKSATANTIIGRNRFNSSSSSRSQTKQCQSETRLRSSKQISVIDTPGLY

Drer060_1 LRIVLLGKTGAGKSSAANTILGRQNFEADDSADSVTKTCERGQVEIDGKKVSVIDTPGLF

Drer061_1 --MVLLGKTGSGKSASGNTILGKFQFKSEASPKSITRSCERHVAEVEGKLISIIDTPGIC

Drer061_2 LRIVMVGKTGAGKSATGNTILRQKVFKEELSAKSVTKKCQKHQREVSGRIISIIDTPGLC

Drer062_1 LNVLVCGSDGSLKSSISELILQ-----HTHR----RSESMRTDVDLHGRLINVLELPALF

Drer062_2 LRIVLLGKTGVGKSSTGNTILGRDVFAAGTSQESVTEESQRETSKINGRRITVIDTPGLF

Drer063_1 --MVLVGKTGSGKSASGNTLLGDFQFKTKMSSKSITRSSERYLAEVEGKIISVIDTPGIC

Drer064_1 RRMVLVGKTGAGKSSSGNTILGRKAFRAVSSGSTVTKDCWKETAEVAGRGITLVDTPGLF

Drer065_1 LRILLLGKSVSENSRVGNLILGRSAFDSEAPPDVV----ERVEGRLKHRHVTLINSPQLL

Drer065_2 --------DGSLKSSISELILQ-----HTHR----RSESVRTDV------INVLELPALF

Drer065_3 VRIVLLGKTGVGKSTTGNTILGRKAFTAETSHQPVTKESQRETCEINGRQITVVDTPGVF

Drer066_1 MRIVLLGKTGIGKSATGNTILGRTAFKSEASFESVTKESQRETSEINGRSITVIDTPGLF

Drer066_2 V-LSVLGIQSSGKSTMLNAMFGL-QFPCTRGAMQLVKVSDEMKTQMNVDYILVVDTEGLC

Drer067_1 --------DGSLKSSISELILQ-----HTHR----RSESVRTDV------INVLELPALF

Drer067_2 VRIVLLGKTGVGKSTTGNTILGRKAFTAETSHQPVTKESQRETSEINGRQVTVVDTPGVF

Drer068_1 LRIVILGKTGVGKSSTGNTILGRDVFKAGESQESVTEESQRESSEINGRRITVIDTPGLF

Drer069_1 RRMVLVGRTGAGKSSSGNTILGRKAFRAAKSASSVTKECWKETGEVDKHQLVLVDCPGIF

Drer070_1 LRIILLGKTGSGKSSTGNTILDNKYFKADFSAVSVTKTCESGKLKIGERIISVVDTPGLF

Drer071_1 LRIILLGKTGSGKSSTGNTILDNKYFKADFSAVSVTKTCESGKLKIGERIISVVDTPGLF

Drer072_1 LSLVLLGKTGVGKSATGNTILGRQAFKSEKSGSSVTKDVLEESGIVCGFPVTVYDTPGLY

Drer072_2 RRIVLVGKTSVGKSASGNTILGQREFRSRRSMSSVTHESTEAQATVSGRSVSVVDTPGLF

Drer073_1 RRMVLVGRTGAGKSSSGNTILGRKAFRAAKSASSVTKECWKETGEVDKHQLVLVDCPGIF

Drer074_1 LRMVVVGRTGAGKSSSGNTILDRKVFRAAKSSSSVTKECWKETGEVAGREVTVVDTPGLF

Drer074_2 LKL---------KSKEDELR------------REYEQKLQDKERK-------------LE

Drer075_1 LRIVLLGKTGAGKSATGNTILGRNAFKVARFCKSTTQHCEKHEGLVEGRSITVIDTPGVF

Drer075_2 KNIMLLGVTGAGKSASGNTILGENKFTVKQSFSSVTKNCQLET----GQSITVIDTVGLS

Drer076_1 LRLVLLGKTGAGKSATGNTILGEKRFNDDLSMSSVTKECQRENTSTEGRNLLLVDTPDFT

Drer076_2 IMQR----EERGDAEMVKQVL--------KKEKKLNEKCQETEREMR-----------RF

Drer077_1 RRMVLVGMTGAGKSSSGNTILGRNCFIAAKSPSSVTRECGKESGEVAGREIHLVDTPGMF

Drer078_1 IRIVLVGKTGVGKSAAANTILGENAFRSDVSSSSVTTDCDKVRKNVNGQKVAIIDTPGLF

Drer079_1 INIVLLGKTGVGKSSSGNTILGENRFRSGRSLSAVTDTSSIEKSVTNGRSVSVIDTPGFF

Drer080_1 LRIVLLGKTGAGKSATGNTILGRKVFKVGDYSESTTQHCEKHEVLVEGRNISVIDTPGVF

Drer081_1 LRLVLLGLQGVGKSAVGNTILNKEEFHSDISAASLTLTSEQKDAVVFGRRVTVVDTPGIL

Drer081_2 LRIVLLGKTGVGKSAAGNTILGAEYFKEDFSSLSMTKVCWKATKNINSTKVAVIDTPGLF

Drer082_1 INIVLLGKTGVGKSSSGNTILGENRFRSGRSLSAVTDTSSIEKSVINGRSVSVIDTPAFF

Drer083_1 MRILLLGKSVSENSRVGNLILGRSAFDSEAPADVV----ERVGGRLKDRHVTLINSPQLL

Drer083_2 -----------LKSSISELILQ-----HTHR----KLESVRTDVDLHGRLINVLELPALF

Drer083_3 LRIVLLGKTGVGKSSTGNTILGREAFKAEDYFESVTKQSQRETSEINGRRITVIDTPGLF

Drer083_4 ---REMER-------------------------------EKQEQQMK-----------IL

Drer084_1 INIVLLGKTGVGKSSSGNTILGENRFRCGRRLSAVTDTSSIEKSVTNGRSVSVIDTPGFF

Drer085_1 KRIVLLGKTGDGKSSAGNTILKQEVFKSKASPESVTVECVSGDRKIDGKKITVIDTPGLF

Drer086_1 TTVVLLGNDRKKKDHIGNIILDKPHFQTKDTC-------EKILHTIDGQKVCIINTPDLF

Drer086_2 LTIVLLGQTGSGKSASGNTILKKQAFKSHASSVPVTTECQMEKGVVFEKNITVIDTPDFF

Drer087_1 INIVLLGKTGVGESSSGNTILGENRFACKKSLSAVTNTSSIEKSVTNGRSVSVIDTPGFF

Drer088_1 KRIVLLGKTGDGKSSAGNTILKQEVFKSKASPESVTVECVSGDRKVYGKKITVIDTPGLF

Drer089_1 KRIVLLGKTGDGKSSAGNTILKQEVFKSKASPESVTVECVSGDRKVYGKKITVIDTPGLF

Drer090_1 LNVVLLGKTGAGKSSSGNTILGRQAFITQKS---VAQDVTVESGSFGELPVSVYDTPGLS

Drer090_2 RRIVLLGKSGVGKSAVGNTILGQKEFTSVMSTNSVTRVCSAAQSTVSGRSVSVVDTPGFF

Drer090_3 RREEEEREQQVDKKIQEEIEIKEEMVA-----KNLKAEKQSEEEQRR-----------QE

Drer091_1 INIVLLGKTGVGKSSSGNTILGENRFTCKKSLSPVTNESRIEKSDTNGRSVSVIDTPGFF

Drer092_1 LRIVLLGNPGAGKSSSGNTILGQKAFLSQICTSSVTRGCSEAQATVSGRSVSVVDTPAIC

Drer093_1 RKIVLLGKTGDGKSSSGNTILGKQTFTTESSPQSITSESTKGVAQVDGRTVTVIDTPGIF

Drer094_1 INVVLLGKTGSGKSSSGNTILGRQAFISKRRSVSVTRDVAVESGSFCELPVTVYDTPGLL

Drer094_2 RRIVLLGKSGVGKSAAGNTILGQREFVSVMRMNSVTRICSAAQATVSGRSVSVVDTPGLF

Drer095_1 RRIVLLGKSGVGKSTVGNTILGQKKFSCQIRSHSVTRVCSAAQATVSGRSVSVVDTPGFF

Drer096_1 LRILLLGKSVSENSRVGNLILGRSAFDSEAPPDVV----ERVGGRLKHRHVTLINSPQLL

Drer096_2 CK------HEEEQNKMKIQI------------EELNREREELIKK-------------LV

Drer096_3 IRIVLLGKTGVGKSAVGNTILGQKEFSCQISSHSVTLVCSEAQAKVSGRSVSVVDTPGFF

Drer097_1 LRIMLLGARGSGKSSTGNTILAYNAFKSDMQLSRVTQFCDKASGNIGGRPVAIIDTPGLN

Drer098_1 LRILLLGKSVSENSRVGNLILGRSAFDSEAPPDVV----ERVGGRLKHRHVTLINSPQLL

Drer098_2 CK------HEEEQNKMKIQI------------EELNREREELIKK-------------LV

Drer098_3 IRIVLLGKTGVGKSAVGNTILGQKEFSCQISSHSVTLVCSEAQAKVSGRSVSVVDTPGFF

Drer099_1 LRIVLLGKTGSGKSSTGNTILGRDAFR--VSFLSSTQTCERRNAVISGRNISVIDTPGLL

Drer100_1 IRIVLLGDRSIGKSASGNTILGQKVFRSERSSFAVTRECSIAEATVSGRSVSVVDTPGFF

Hmag001_1 KVVVVVGSTGTGKSTIINMLYNDSVLKIGATSNSVTKKM------FNARDLTIADTVGLS

Hmag002_1 FNILLLGPTGAGKSHLINVFFNKPVCKSDTSFKSVTREIEKKSNSYVNKEIVVTDTVGLC

Hsap001_1 LRIVLVGKTGAGKSATGNSILGRKVFHSGTAAKSITKKCEKRSSSWKETELVVVDTPGIF

Hsap002_1 LRIVLVGKTGSGKSATANTILGEEIFDSRIAAQAVTKNCQKASREWQGRDLLVVDTPGLF

Hsap002_2 ------------------------------------RLKQREEVLRK-----------IY

Hsap003_1 LRIILVGKTGCGKSATGNSILGQPVFESKLRAQSVTRTCQVKTGTWNGRKVLVVDTPSIF

Hsap004_1 RRLILVGRTGAGKSATGNSILGQRRFFSRLGATSVTRACTTGSRRWDKCHVEVVDTPDIF

Hsap005_1 LRLILMGKTGSGKSATGNSILGRDVFESKLSTRPVTKTSQRRSREWAGKELEVIDTPNIL

Hsap006_1 LRIILVGKTGTGKSAAGNSILRKQAFESKLGSQTLTKTCSKSQGSWGNREIVIIDTPDMF

Hsap007_1 LRLLLLGKCRSGKSATGNAILGKHVFKSKFSDQTVIKMCQRESWVLRERKVVVIDTPDLF

Hsap007_2 LTVLLVGKRGAGKSAAGNSILGRQAFQTGFSEQSVTQSFLSESRSWRKKKVSIIDAPDIS

Hsap007_3 LNIVLVGRSGTGKSATGNSILGSLVFTSRLRAQPVTKTSQSGRRTWDGQEVVVVDTPSML

Hsap008_1 INLALFGMTQSGKSSAGNILLGSTDFHSSFAPCSVTTCCSLGRSCLEALQVQVLDTPGYP

Lgig001_1 YRVVLVGKTGVGKSASGNSILNERAFTSQNSAMPVTQRCQRKQRYNHEALLDIIDTPGLF

Lgig002_1 MRAAILGKTGVGKSSLGNSLLGKKIFKSTRSASSVSQQCEFGEAQTSGK-LLVVDTPGLF

Lgig003_1 YRVVLVGKTGVGKSASGNSILNERAFSSQNSAMPVTQRCQRKQRYNHEALLNVIDTPGLF

Lgig004_1 YRVVLVGKTGVGKSASGNSILNERAFTSQNSAMPVTQRCQRKQRYNHEALLNVIDTPGLF

Lgig005_1 -SLVLVGKTGMGKSSLGNSLLGEKPFKSKKSAGSITKQCERKRATLGGTLLTIVDTPGLF

Lgig006_1 YRVVLFGKTGIGKSSVGNSLLGKTIFQFTSYASSATEWCKMGEAQTSGK-LLVVDTPGLF

Lgig007_1 YRIVIIGKTGTGKSSLGNSILENRPFPSQTSANSVTKTCQRGFVFHKKTSLVVIDTPGLF

Lgig008_1 YRIVIIGKTGTGKSSLGNSILENRPFPSQTSANSVTKTCQRGFVSHKKTTLIVIDTPGLF

Lgig009_1 IRVILIGKTGAGKSYLGNALMGRELFKSSVTAESVTTFCASGKRKIKSRSLKVIDTPGLK

Lgig010_1 IRILLMGKCGSGKSSVGNLLLGQKVFQVSKTCITKTKQSQLASRTLEGRVLVIVDTPGYC

Lgig011_1 IRILLMGKSGSGKSSVGNLLLGQKVFQVSKTCITKTKQSQLASRTLEGRVLVIVDTPGYC

Lgig011_2 RHIILLGKSGIGKSSLGNYLLGAKKFKTSIMEERRTKECHIEL----TDHMVVMDTPGFF

Lgig012_1 IRVVMLGKTGVGKSLLGNNILGKDIFSSKVGAQSCTKELTRAERMLDYYKLVLVDTPGLF

Lgig013_1 IRMVLVGETGTGKSSTGNSILAQNVFPVSTKGTPTTRTCAAGETTRFGTKFVIVDTPSYF

Lgig014_1 IRVVMFGKTGVGKSLLGNNILDHAKFPNLGCTQSVTIDNNRGERVMSYRKLVLVDTPGLH

Lgig015_1 --LVLVGRTGSGKSSLGNSLLSEKPFKSEKSANSITTQCQLARAILKGTLLTIVDTPGLF

Lgig016_1 --ILLLGKTGSGKSSVGNQLIGSKVFTVSRND--GTQQSQLARRTLEGRELVIVDTPGYC

Lgig017_1 IRVLLVGKCGSGKSSVGNQLIGSKVFTVSNTD--GTQQSQLARRTLEGRELVIVDTPGYY

Lgig018_1 --VLLLGKTGSGKSSVGNQLIGSKVFKVSRNY--GTQQSQLESRTLEGGELVIVDTPGYC

Lgig019_1 RHIILLGKSGIGKSSLGNYLLGAKKFKTNIMEERRTKECHIEL----TDHMVVMDTPGFF

Lgig020_1 --IILLGKSGIGKSSFGNYLLKAKKFPTRELEDGETGECQIER----TDSMVVVDTPGFF

Lgig021_1 --IILLGKSGIGKSSFGNYLLRAQKFRTRELEDGETEECQIER----TDSMVVVDTPGFF

Lgig022_1 KTVLLLGKSGIGKSSVGNVLLGEKKFAASMMEERGTKTCQIEE----SDQLLVVDTPGFF

Lgig023_1 --VLLLGKTGSGKSSVGIQLIGSKVFTVSRKD--GTQQSQLARRTLEGRELVIVDTPGYC

Mbre001_1 PRILLMGQKRSGKSSIQQVVFHKMSPNETLFLETTNHVVKDEISNSSFVQFQIWDFPGFF

Nvec001_1 FKVIVVGRTGVGKSHLVNTLMGEYVVEEGQDLDPCTSTVSKHEKRIGRTRVTVWDSPGLQ

Oluc001_1 CTILLLGKSGVGKSAVINSLLGEGSAPSGTDDEDATKKVQLIEKKIHGMTLRLIDTPGLQ

Oluc002_1 MTVIFIGKQGVGKSSTVNTLLNERVAPSSP-FQPENVRPLLAGRVAAGFTLNVLDTPGLL

Ppat001_1 TTLVLVGRTGNGKSATGNSLLGSTVFRSRASSAAVTSTCEVQETAPDGRRLRVIDTPGLF

Ppat001_2 L--------------------------------------QDIKRD-------------LE

Ppat002_1 ITIVLLGKGGVGKSSIVNSLFSERVAAVSA-FRSETLRPRQYSRSKDGFKLTVIDTPGFV

Ppat003_1 CTILVLGKTGVGKSATINSIFDDRKSVTSA-FKPSTNKVQEIVGTVHGIKVRVIDTPGLL

Ppat004_1 ERVILFGRTGSGKSTVAHMLTKGEEFEHGSDAKGVTRKVKRGQ----GRGWLVTDTPGFG

Ppat005_1 CTILVLGKTGVGKSSTINSIFDERKSVTSA-FKPSTNKVQEVIGTVHGIKVRVIDTPGLL

Ppat006_1 CTILVLGKTGVGKSATINSIFDECKTVTSA-YYPSTTKVHEVSGTVLGVKVRFIDTPGLL

Ppat007_1 LTVLVVGKGGVGKSSTVNSIIGERVTVVSA-FQSETLRPLQCARTRAGFTLNVIDTPGLI

Ppat008_1 GKVILFGKTGSGKSTIANALVTGQVFGVSAGFRGCTSSLQTAS----GRGWEVVDTIGLG

Ppat009_1 LTVLVVGKGGVGKSSTVNSIVGERVTIVSA-FQSETLRPLQCARSRAGFTLNIIDTPGLV

Ppat010_1 KKVILFGKTGSGKSTVSNALVTNVVFQSSAGFSGCTWAIQTGA----GKGWEVIDTVGLG

Skow001_1 LILVLVGRTGAGKSATGNTILGRQQFRSSRSTVSKTRLNAWAKC-TQDRSIVVIDTPGSF

Skow001_2 LVFVLIGRTGCGKSATGNSIVGGKTFDAERRLVSKTKTTRYGKRTFDGKDLVVIDTPGVF

Skow001_3 LVFVLIGRTGCGKSATGNSIVGEKAFHSERCLVSTTKTTRYGKRTFDGKDLVVIDTPGVF

Skow002_1 LVFVLIGRTGCGKSATGNSIIGGNTFDAERRLVSTTKTTRYGKRTFDGKDLVVIDTPGVF

Skow003_1 LTLVLVGRTGSGKSATGNTILGKPHFMSVRSMSSKTRSIAWGR--EQGRKLVVIDTPGFF

Skow004_1 LTLVLVGRTGSGKSATGNTILGKPHFMSVRSMSSKTRNIAWAR--EQKRKLVVIDTPGFF

Skow005_1 LTIVLVGRTGSGKSATGNTILGKPHFMSVRSMSSKTRNIAWAR--EQGRQLVVIDTPGIY

Skow006_1 LCLVLVGMNGSGKSATGNTLLKRDSFTSRRSLIPTTQKTAWGKTI--GRDILVIDTPPMI

Amil001_1 DTR-GDKVITEQIRKFFNTQGIHVDAICFIAQAGRLTPTQQYVFDRILAMFGKDIKKNIL

Amil002_1 DTNLKNDAVITKIKEFFQTDAPGVNLVLFVFRKGRFTQEERQTFDYIIKNFKKEISNYSA

Amil003_1 SKKIDQASIFKEIHQHCTADC-EIHLILFTFRYGVYEQREIDIVTAICEHFQDQIPKISA

Amil004_1 GTPSQDDAEIVKVKKFFQNFSPGINLVLFVLRKGRFTDEDRRAFEYFITHFSDEISNFSA

Amil005_1 R----LEDLTRDTTSAWKAMKGGIHAVIWVMSAVRFQGNYRENLAFVKHILNRESI-TII

Amil006_1 DTKIVQETTLKELARMVLYAPRGFNAILLLAKFGRFTAEDDEALQLLLKFFSAEAQKYMI

Atha011_1 DLSVSAEYISKEIVRCLTLAEGGIHAVLLVLSARRITQEEENTLRTLQALFGSQILDYVV

Atha012_1 DLAVSAEFISKEIVNCLILAREGLHAVVLVLSLSRISQEEENALCTLQMLFGGKIVDYLI

Atha013_1 DLSVSAEFIGKEIVKCLTLADGGLHAVLLVLSVRRISQEEEMVLSTLQVLFGSKIVDYLI

Atha014_1 DLSTAADFIGKEIVRCISLAEDGIHAILLVFSVRRLAEEEQTVLSFLQALFGSKIADYMI

Atha015_1 SLSPSTEFTCREILRCFSLTKEGIDAVLLVFSLKRLTEEEKSALFALKILFGSKIVDYMI

Atha016_1 DLSTAADFIGKEIVRCISLAEDGIHAILLVFSVRRLAEEEQTVLSFLQALFGSKIADYMI

Atha017_1 DLSVSAEYLSQEIINCLVLAEDGLHAVVLVLSVRRISQEEEATLNTLQVIFGSQIIDYLV

Atha018_1 SASSTTDFTIREIVRCLLLAKGGIDAVLLVFSLRRLTEEEQSTLRTLKILFGSQIVDYII

Atha019_1 DSFVPGDDISNEIINCLTMAEEGIHAVLLVLSARRISKEEESTVNTLQCIFGSQILDYCI

Atha020_1 DLSVSAEFISKEIINCLRLAEGGLHVVVLVLSVRRITQEEENTLSTLQVLFGNEILDYLI

Atha021_1 DLSVSTDYMNKEIINCLTLTDGGLHAVVLVLSVGDILKEEEAALNKLQLLFGSKIVDYLV

Atha022_1 DLSVSEDYISKEIINCLTLAEEGVHAVLFVLSMKRITQEEEYALNTLQRIFGSKILEYLI

Atha023_1 ESSVSGDYLSKEIMNCLTMAEEGIHAVLFVLSITRISQREEFTFNTLQQIFDDKILDYFI

Atha024_1 SLFPSNESTIREILKCSHLAKEGIDAVLMVFSLRRLTEEEKSVPFVLKTLFGDSIFDYLI

Atha024_2 E------QKLNQEQARLEAENK--------------LHEESSEEKILKEKLERE------

Atha025_1 DSSVSANYITTEILKCLTMAEGGIHAFMFVLSAGRITQEEESTLDTLQLIFDSKILDYFI

Atha026_1 EGGY-NDQAINIIKRFLLNM--TIDVLLYVDRLDRVDDLDRQVVGAITDAFGKEIWKKSA

Atha027_1 EAGY-NHQALELIKGFLVNR--TIDVLLYVDRLDRVDELDKQVVIAITQTFGKEIWCKTL

Atha028_1 ------------------------------------------------------------

Atha029_1 PSSSSNRKILLSIKRYVKKR--PPDVVLYLDRLDDMRYSDFSLLQLITEIFGAAIWLNTI

Atha030_1 PSSSSNRKILLSIKRYVKKR--PPDVVLYLDRLDDMRYSDFSLLQLITEIFGAAIWLNTI

Atha031_1 PSWSDNEKILKSVRAFIKKS--PPDIVLYLDRLDSRDSGDMPLLRTITDVFGPSIWFNAI

Atha032_1 PSWSDNEKILNSVKAFIKKN--PPDIVLYLDRLDSRDSGDMPLLRTISDVFGPSIWFNAI

Atha033_1 SSATENQEVL------------------------------------IARCLGIVLAENNM

Atha034_2 E----------------------AEVIAM-------KERSRKEHDHTMNMAHEHAL----

Atha035_1 SAAMDNAKMLSSVKKVMKKC--PPDIVLYVDRLDTRDLNNLPLLRTITASLGTSIWKNAI

Bflo001_1 DTQASMGEVSEEIARITTIFSAGLHALLLVIKAGRFTEQDVKVVQILKEIFGDNFMKYVV

Bflo002_1 DTHDSMGDICEEISRITTIFSAGLHALLLVVSVGRFTEQDVKVVEILKEIFGEAFMKYVV

Bflo003_1 DTDVPHETVIQEISRVHLLAHSGIHAIILVFRFPRFTDEEKRAYDSLLQMFRQDILKHVI

Bflo004_1 DTSADPEVVGEEIARMATILSEGLHALLLVVRLSRFTQEEIDAIAMLKELFGKNFMQYVV

Bflo005_1 DTDAKGTKILAEVSKCRQECPNGVTAVLLVIPFGKFTKEEENSIGDLKRLFGEKLFKYGI

Bflo006_1 DTSMPYETIVEEISKVHVLAHGGIHAVILVFRPDRLTEEEKMAYNSLIQKFQTDILKHVI

Bflo007_1 DTDVPHETVVKEISRVHFLAYSGIHAIILVFKFQRLTDEEKRAYDSLIEMFRKDILKHVI

Bflo008_1 DTGN-VEDICKELCRIVTFFPNGLHAVILVLRRGRFTWEEAETIKLYELMFGERLLKHSL

Bflo009_1 ATVNEIQKISEELCKIPTVFHDGIHALILVISGMRFTEEDDNALKNIQRVFGEGFLDHTV

Bflo010_1 ESL--ENDAAREVARCLVETRDGIDALLLIHKFGRFTDQQKTLLAALEKYFGKEIYKYII

Bflo011_1 STDD-LKEIAQQLCRIVTRFGDGLHALVVVISSRRFTESETKAINIFQHLFGNRFVDYAI

Bflo012_1 DTGN-VTDICKELCRIVTFFPEGLHTVILVLRRGKFTWEEAETLRIFELMFGERFLKHSL

Bflo013_1 DTGNNDEHTATILTQVATMFPNGLHALLFVVNHTRFTKEDALAVDLLRHVFGERFLQCSV

Bflo014_1 HTDTDKDRLVDQLSRIAATYHIGLHSMLLVISGRRFTQEDKDAVQCLRAVFGDRLLEYTI

Ddis001_1 DSQGEDSNNMIKIQRYLSGK--TIHCVLFVEKFTRFDGAHQLVINQFTEKLGPQLWRNAA

Drer001_1 DTHLSNEQVITEVVNCIRLATPGPHVFLLIIAIGRFTKEEKKTVELIQKVFGQQVHRHMM

Drer002_1 QTHLNQLQVIQAVRECLSQCAPGPHVIILVLQYNDFTELDRDRVKYILSLFSQKAIKHTI

Drer002_2 N--LSEEEVMRQTLRCVSLCHPGVHLFILIIP-DPLNNEDRAEVEKMQ-IFSFKINKHMM

Drer002_3 DTQLSNEEIKREISNCISMILPGPHVFLLVISLGRFTQEEQESVKIIQEIFGENSLKYTI

Drer002_4 DLTLSKEQVQEQIMKCVHQSAPGPHVFVIVVSLGKISQEKGEILDMITMMFGPEAAKFSV

Drer003_1 QVDPPQQQFTNRVSIYMEQFAPGPHVFILVLQYKDFTKQDKHRVENVLNLFSQKAIKHTI

Drer003_2 -NQLSEEEMTQETLNCLYLCDPGVDLFILVTP-VPLTKEDRAEMEKIHRTFYSK--EHFV

Drer003_3 N--LSEEEVMRQTLRCVSLCQPGVHLFILIIPEEPLNNEDRAEMEKMQKIFSSRLNKHMM

Drer003_4 DTKLSNEEIKREISNCISMILPGPHVFLLLISLGRFTQEEEKSVKLIQETFGENSLIFTI

Drer003_5 DTTLSTDEVVEGIMESVSLSAPGPHVFIIVLSLEKITQEEKDLLDLITKMFGPEAAKFSI

Drer004_1 DTTLTNDQVVEEIVKCVSLSAPGPHVFIIVVSLGRITKEEADTIDLIKKIFGPKSAQFSI

Drer005_1 IPDLFSSQITQTVKEIVNLSAPGPHVIILILQQNHFTEEDRRRVKYVLNEFSDEAIKHTI

Drer005_2 -TLLSQKEEMRQSLRCVSLCDPGVHVFLFVIP-DPLTDEDKTETEKFQKIFSSEIKNHIM

Drer005_3 DTKLSQEEIQREITECISLILPGPHVFLLLIPVGRFTQEEENAVKKIQQTFGKNSLKYTI

Drer006_1 NTHLTDEELQNEMMESLHLCYPGPHVFLLIINLEDFTNDQKNVVQKVLKSFGSHVLKFTM

Drer006_2 DTSISQEELKKELVKCVELSVPGPHAFLLVLRVDRFTDEEKKTVKLIQENFGEKATRYTI

Drer007_1 NPDTTDHQITQTVKECVEMSDPGPHAFILVLQYKDFTEDDMRRVKYVLNTFSEDALKHTI

Drer007_2 -SRLSEDEVMRQTLHCVSLCDSEVHAFLLIIP-APLTDEEKAEIETIRRTFDSC--EQFI

Drer007_3 DTTLTKKQVVEEIVKCVSLSAPGPHVFVIVVSLGRFTKEEADTIDLIKKIFGQKAAQFSM

Drer008_1 HTNISDDQITQTVRECVSLSDPGPHVFMIVLQYKDFTDEDRYKVRSVLKEFSEDAIKHTI

Drer008_2 -TRLSEEEVMNQTLRCVSLSDPGVHAFLIIIP-VPLTVEDKAEIDKVQKIFDSR--DHFI

Drer008_3 DTALTNDQVVEEIVKCVSLSAPGPHVFVIVVSVGRITKEETETIDLIKKIFGLKSAQFSI

Drer009_1 HTNISDDQITQTVRECVSLSDPGPHVFMIVLQYKDFTDEDRYKVRSVLKEFSEDAIKHTI

Drer009_2 -TRLSEEEVMHQTLRCVSLSDPGVHAFLIIIP-VPLTVEDKAEIDKVQKIFESR--DHFI

Drer009_3 DTALTNDQVVEEIVKCVSLSAPGPHVFVIVVSVGRITKEETETIDLIKKIFGLKSAQFSI

Drer010_1 DTSKEKDIIKREIVKCIKVSAPGPHAFLLVIQIGRFTAEEQRAVQALQELFGEDASNYMI

Drer011_1 DPDTSDDQITKTVRECAEISDPGPHVFILALQYKDFTEDDVIRVKHVLSKFSEEAINHTI

Drer011_2 -SRLSEDEVSSQTLHCVSLCHPGVHAFLIIVP-VLLTDGDKLEVEKILNIFNTK--QHII

Drer011_3 DTTLPNDQVVEEIVKCVSLSAPGPHVFVIVLTLLRFTKEETDTVDLIKKIFGTKSAQFSI

Drer012_1 -TRLSEEEVMHQTLRCVSLCDPGVHAFLIIIP-VPLTVEDKAEIEKILKIFDSR--DHFI

Drer012_2 DTTLTNDQEVEEIMKCVSLSAPGPHVFVIVLSLGRFTKEETETIDLIKKIFGPQAAQFSI

Drer013_1 QVDPPQQQFTNRVSIYMEQFAPGPHVFILVTPVTPLTKEDRAEMEKIHRTFYSK--EHFV

Drer013_2 N--LSEEEVMRQTLRCVSLCQPGVHLFILIIPEEPLNNEDRAEMEKMQKIFSSRLNKHMM

Drer013_3 DTKLSNEEIKREISNCISMILPGPHVFLLLISLGRFTQEEEKSVKLIQETFGENSLIFTI

Drer014_1 DTTLKNEVVVEEIVKCVSLSAPGPHVFVIVLSLGRLTKEETDTIDLIKKIFGTKAAQFSI

Drer015_1 DTSLSNEVIQQEIMRCIELSAPGPHVFLLLISIGPFTREERETLELIKMTFGQNAQSYTM

Drer015_2 KLKPTQEAVKQKAS---ELSRFNPHVIILVLQLSGFSEKQKD------------------

Drer016_1 NPDTTDHQITQTVRECVEMSDPGPHAFILVLQYKDFTEDDMRKVKYVLNTFSKDALKHTI

Drer016_2 -SRLSEDEVMRQTLHCVSLCDSEVHAFLLIIP-APLTDEEKAEIETIRRTFDSC--EQFI

Drer016_3 DTTLTNDQVVEEIVKCVSLSAPGPHVFVIVLSLGRFTKEETDTIDLIKKIFGTKSAQFSI

Drer017_1 NPDTTDHQITQTVKECVDMSDPGPHAFILVLQYKDFTEDDMTRVKYVLNTFSEDALKHTI

Drer017_2 -SRLSEDEVMHQTLHCVSLCDSEVHAFLLIIP-APLTDEEKAEIETIKRTFDSC--EQFI

Drer017_3 DTTLTNDQVVEEIVKCVSLSAPGPHVFVIVLSLGRFTKEETDTIDLIKKIFGTKSAQFSI

Drer018_1 HTHISDDQITQTVRECVSLSDPGPHVFLIVLQYEDFTDEDRCRVRSVLKEFSEEAIKHTI

Drer018_2 -SCLSEDEVMRQTLHCVSLCDPGVHAFLLIIP-VHLTDGVKLEVDKVLKIFDTK--QHFI

Drer018_3 DTALTNEQVVEEIAKCVSLSAPGPHVFIIVLTLGRFTKEETETIDLIKKIFGTKSAQFSI

Drer019_1 DTGIDNAQIMKEIVKCVSMAAPGPHVFLLVIPLVRFTDEEKDAVKMTQEMFGDKSRMYTM

Drer020_1 KTKLKLEAATQKAF---ELSRFNPDVIILVLQHNEFRQFHKDRLPSVLIYFGEQAKNRTM

Drer020_2 DTGVDNHETMKEVVKCVSMAAPGPHVFLLVISLGRFTKEEKDAVKIIQERFGDQSSMYTM

Drer020_3 M------ENEDEIN----------------------RKKEDIRDKYEAEKEQ--------

Drer021_1 EIP----SEDQDLRSLIDLAFPGPHALLLVLKSGTLTDVENGALKLINVIFGVGASDYVI

Drer021_2 DTSLSNEVIQQEIMRCIELSAPGPHVFLLLISIGPFTREERETLELIKMTFGQNAKSYTM

Drer022_1 SPPVS--PEEQDLKKFFHLSCPEPHALLLVLKPGTITKQDRDTLQLITTVFGTGAFEYVI

Drer022_2 DTSLSNEVIQQEIMRCIELSAPGPHVFLLLISIGPFTQEERETLELIKMTFGQNAKSYAM

Drer023_1 SPPVS--PEEQDLKNFFHLSCPEPHALLLVLKSGTITKQDRDTLQLITTIFGTGAFEYVI

Drer023_2 DTSLSNEVIQQEIMRCIELSAPGPHVFLLLISIGPFTQEERETLELIKMTFGQNAKSYAM

Drer024_1 DTELSNEEIQREISNCISMILPGPHVFIIVLSLGRFTKEEETSVKFIKETFGEHSLMFTI

Drer025_1 DTSVSKEDTEREIKYCMSYSAPGPHAFLVVLKLERFTEENAKALEYIERLFGKEAINYTM

Drer026_1 DPDVDKDKLQKLEEQLLSACSAGLSSVLLTVPLEEPLQNEEEMLDYIKRLFDPEVQKYIM

Drer026_2 DTELSEKEIITEIAKCITYASPGPHAFIIVIKVGRFTEEEKNTIQQLKEVFGEQMEKYSM

Drer027_1 DKS-SQKGIQSDITECISMTLPGPHVFLLLISVGQFTVEEENTVKKIMETFGENSLMYTM

Drer028_1 DPKLSEHEIQQKIGKVLQKSEVGEWAFLIVIKADSFTEEERITVKKIEKLLGERRFQKTW

Drer028_2 -SRLSEEEVMHQSHQCVSLGDPGVHAFLFFIS-DPLTEEDKAEMEEIQKIFSSKINKHMI

Drer028_3 DTGIDNAQIMKEIVKCVSMAAPGPHVFLLVISLVRFTDEEKDAVKMIQERFGDQSSMYTM

Drer028_4 M------EKVEEIK----------------------RKEKELRDKYEAEMEQ--------

Drer029_1 DTELSNEEIQREIRHCISMILPGPHVFIIVLSIGRFTEESETSVKIIQKMFGQNSLMFII

Drer029_2 M------DRVREIEEIKK------------------LEEEKDRMKMMMEEEKQ-------

Drer030_1 DTELSNKEIQREISNCISMILPGPHVFIIVLNLGRFTKEEEKSVKFIQKTFGEKSLMFTV

Drer031_1 DTELSNKEIQREISNCISMILPGPHVFIIVLNLGRFTKEEEKSVKFIQKTFGEKSLMFTV

Drer032_1 DSSLTRDELMDRIKQCLPLSAPGPHVFLVVIQLGRFTDEEAEAVKTIQNIFGEESSTYTM

Drer033_1 DTSLPVDEVVNRIKLCIPLSAPGPHVFLVVIQLGRFTDEEEEAVKIIQAAFGEESSIYTM

Drer034_1 SPAVS--PEEHDLRRFFHLSCPEPHALLLVLKSGTVTQQDRAALQVITTVFGTGAFDYVI

Drer034_2 DTSLSNEVIQQEIMRCIELSAPGPHVFLLLISIGPFTREERETLELIKITFGQNAQSYTM

Drer035_1 DTGVDNVETMKAIVKCVSMAAPGPHVFLLVIQLGRFTKEEKDAVKIIQERFGDQSSMYTM

Drer036_1 DTTLTNDQVVEEIMKCVSLSAPGPHVFVIVLTLGKFTKEETETIDLIKKIFGPKAAQFSI

Drer037_1 DTELNNEEIQREIRRCISMILPGPHVFIIVLTIGRFTEESETSVKIIQKMFGQNSLMFII

Drer038_1 DSTLTEPEMKEEITKCVEMSAPGPHVFLLVIRLDKFTEEEKNTVKWIQENFGEEAARYTV

Drer039_1 DTELSNEEIQREIRHCISMILPGPHVFLLLIPLGRFTKEEETSVKIIQETFGEHSLMFTM

Drer040_1 DTSLPVHEVVNRIKLCIPLSAPGPHVFLVVIQLGRFTDEEEEAVKIIQAAFGEESSIYTM

Drer041_1 DTKMNPEELMTEIARSVYISSPGPHAFLIVLRIDRFTEHEQQIPKTIEWLFGEGVLKYSI

Drer042_1 -------EFLRQFIGLPEFNTQDPLVYLLVIKSDRFTAEEKNTVESIEEFLPDFLKKNTW

Drer042_2 DLNVKPGIISKEIGRSIHLCSPGPHAFLYVISLSRFTKADESVVVNIEKLFGKGMLKYTI

Drer043_1 DTALTNDQVVEEIAKCVSLSAPGPHVFIIVVSVGRITKEETDTIDLIKKIFGTKAAQFSI

Drer043_2 NPELKLQAITQKVS---ALSSPEPHVIILVLQHRDFSEKQRDRLPSVLNCFGEQAMKHTM

Drer044_1 DTDLTEEQVQHEVISCLSLSSPGPHAFLLVIPIDRYTEEQQRTVQKILEMFNEDISRYTI

Drer044_2 Q------DKIKQIKD---------------------IKKEEQKIQLRVNLWNEMRG----

Drer045_1 NPDTTDHQITQTVRECVEMSDPGPHVFILVLQYKDFTEDDMRRVKYVLNTFSEDALKHTI

Drer045_2 -SRLSEDEVMRQTLHCVSLCDSEVHAFLLIIP-APLTDEEKAEIERIRRTFDSC--EQFI

Drer045_3 DTTLTNDQVVEEIVKCVSLSAPGPHVFVIVLSLGRFTKEETDTIDLIKKIFGTKSAQFSI

Drer046_1 DTELTEEEIQREIRHCISMILPGPHVFIIVLSLGRFTKEEETSVKIIQETFGENSLMFTM

Drer046_2 M------DRVRETEEMKK------------------LEEEKDRMKMMMEEERQ-------

Drer046_3 DTKLRDEEIQQMISKVLQKCSSGLCVFLLVIRADRFTEEERKTVEKIEKILGEKHQKNTW

Drer046_4 DTQMNPEELMMEIARSVYISSPGPHAFLIVFPVNRFTERELQILQKIELMFGEEVLKYSI

Drer047_1 NTDVPKQQLKAELQKCVHLCAPGPHVFLLVLKLGRFTQEERETVKWIQENFGEQALCRMI

Drer048_1 NTGLSEEEVMRQTLRCVSLCHPGVHAFLLIIP-DPLNNEDRAEMEEIQKIFSSRINKHIM

Drer048_2 DTELTEEEIQREIRHCISMILPGPHVFIIVLSLGRFTKEEETSVKIIQETFGENSLMFTM

Drer048_3 DTKISDEEIQQMINKVLQKCSSGLCVFLLVIRADRFTEDDRKTVEKIEKMLGEKHQNNIW

Drer048_4 DTQMKPEELMMEIARSVYISSPGPHAFLIVFPVNRFTKQEQQILQKIELMFGEEVLKYSI

Drer049_1 DTELSNEEIKREISNCISMILPGPHVFIIVLNLGRFTKEEETSVEFIHEMFGKKSLMFTM

Drer050_1 DTKMKQEDLAKEIARSVWLSSPGPHAFLIVFPVIRFTEQEEQIPQMIEKIFGEEVLKYSI

Drer051_1 NTGLSEEEVMRQTLRCVSRCHPGVHAFLLIIP-DPLNNEDRAEMEEIQKIFSSRINKHIM

Drer051_2 DTELSNEEIKREISNCISMILPGPHVFIIVLNLGRFTKEEETSVEFIHEMFGKKSLMFTM

Drer052_1 HTNISDDQITQTVRECVSLSDPGPHVVVLLLQHQQCSAEDQERVEKLQDSFSERLFQHTM

Drer052_2 KTELSEEEVMRQTLDCVSLCQPGVHAFLFIIS-DPLTDEDKAEMEVIQRVFSSRINKHLI

Drer052_3 DTELTNEEIQKEISNCISMILPGPHVFIIVLNLGRFTQEEAKSVEIIQETFGENSLMYTM

Drer052_4 TND---EETDSQLDRREKVR----------------KKKDEECHEKIEEELDE-------

Drer053_1 DTRLTDKEMKKEILKCVEMSVPGPHVFLLVIRLDKFTDEEKNAVKWIQEDFGEEAARYTV

Drer054_1 DTKMKQEDLATEMASSVWLSSPGPHAFLIVFRIDRFTELEEKIPLIIKKIFREEVLKYSI

Drer055_1 DTELTEEEIQREIRHCISMILPGPHVFIIVLSLGRFTKEEETSVKIIQETFGENSLMFTM

Drer055_2 M------DRVRETEEMKK------------------LEEEKDRMKMMMEEERQ-------

Drer055_3 DTKLRDEEIQQMISKVLQKCSSGLCVFLLVIRADRFTEEERKTVEKIEKILGEKHQKNTW

Drer055_4 DTQMNPEELMMEIARSVYISSPGPHAFLIVFPVNRFTERELQILQKIELMFGEEVLKYSI

Drer055_5 DTELTEEEIQREIRHCISMILPGPHVFLLLVPLGRFTKEEETSVKIIQETFGENSLMFTM

Drer056_1 DTELTNEEIQKEISNCISMILPGPHVFIIVLNLGRFTQEEAKSVQIIQETFGENSLMYTM

Drer056_2 M------DKVRETEEMKK------------------LEDEKDRIKMMMKEEQQ-------

Drer057_1 NTGLSEEEVMRQTLRCVSRCHPGVHAFLLIFP-DPLNNEDRAEMEEIQKIFSSRINKHIM

Drer057_2 DTELTNEEIQREIRHCISMILPGPHVFIIVLSIGRFTEEEAKSVNFIKETFGQNSLMFTM

Drer058_1 NTGLSEEEVMRQTLCCVSRCHPGVHAFLLIIP-DPLNNEDRAEMEEIQKIFSSRINKHIM

Drer058_2 DTELNNEEIQREIRRCVSMILPGPHVFIILLSIGRFTEEEAKSVEFIKETFGQNSLMFTM

Drer059_1 DPDVNEEKLQKLEEQLLSACSAS-------------------------------------

Drer059_2 DTELGEKEIITEIAKCITYASPGPHAFIIVIKVGRFTEEEKNTVQQLKEVFGEQMEKYSM

Drer060_1 DTRLTEQEMKPEIEKCVYKSVPGPHVFLLVIRLGRFTEEEKNTVKWIQENFGEEAPSYTI

Drer061_1 DTSMS-EELKKEIVRCVYMSVPGPHVFLLVIRIGRFTEEEKNTVKWIQENFGDEAVRYTI

Drer061_2 DTSIGEEDLKKEIEKCVYMSAPGPHVFLLVLRLDRLTNEEKNTVKWIQENFGEEANRYTI

Drer062_1 NTGLSEEEVMRQTLRCVSRCHPGVHAFLLIIP-DPLNNEDRAEMEEIQKIFSSRINKHIM

Drer062_2 DTELSKEEIKREISNCISMILPGPHVFIIVLSLGRFTKEEAKSVKFIQETFGQNSLMFTV

Drer063_1 DTSMSEEELKKEMERCVYMSVPGPHVFLLVIRLGRLTAEEKHAVKWIQENFGQEAARYTI

Drer064_1 DWNVSEEYLKLEISKCINMTAPGPHAIILVIQLGPFTEEEKLSVEKIRAVFGEGADKHTI

Drer065_1 HTHISDDQITQTVRECVSLSDPGPHVVLLLLQHQQCSAEDQERVEKLQDSFSERLLQHTL

Drer065_2 NTGLSEEEVMRQTLRCVSLCHPGVHAFLLIIP-DPLNNEDRAEMEEIQKIFSSRINKHIM

Drer065_3 DTELTEEEIQREIRHCISMILPGPHVFLLLVPLGRFTKEEETSIQMIELMFGEEVLKYSI

Drer066_1 DTELTNEEIQREIRHCISMILPGPHVFLLLIPLGRFTKEEETSVKIIQETFGENSLMFTM

Drer066_2 ALELS-RHHDNELA--------------------TF------------------------

Drer067_1 NTGLSEEEVMRQTLRCVSLCHPGVHAFLLIIP-DPLNNEDRAEMEEIQKIFSSRINKHIM

Drer067_2 DTELTEEEIQREIRHCISMILPGPHVFLLLVPLGRFTKEEETSVKIIQETFGENSLMFTM

Drer068_1 DTELSNKEIQREIRRCISMILPGPHVFIIVLSIGRFTKEEAKSVKFIQETFGEHSLMFTM

Drer069_1 DTTVSEAETIREMSKCINMTAPGPHAIILVIKLGPFTEEEKLSVEKIRAVFGEAADKHTI

Drer070_1 DTTMSKQKMKDEIVKCVYKCLPGPHVFLLVARLGRFTDEEKSAVKWIQENFGEKAPRHTI

Drer071_1 DTTMSKQKMKDEIVKCVYKCLPGPHVFLLVARLGRFTDEEKSAVKWIQENFGEKAPRHTI

Drer072_1 DTELEEQEIQQKCQSVFQKCDSELCAFCLVIKVDRFTAEERRTVEKIEKMLGQTRLEKTW

Drer072_2 DTQMKQEELMKEISRSVYISSPGPHAFLIVFPVNRFTEYEQQIPQMTELLFGEEVLKYSI

Drer073_1 DTTVSEAETIREMSKCINMGARGPHAFILVINLGPFTDEEKLSVEKIRAVFGEAADKHTI

Drer074_1 DTKASELNLQQEISKCINMTAPGPHAFILVINLGPFTDEEKLSVEKIRAVFGEAADKHTI

Drer074_2 A-----RTEEKRILEKIQAA----------------SEQEKEKMKDLQRLSQRELKYRRF

Drer075_1 HMFISERQVKAEIEKSLEMSAPGPHVFLLVIRLGRFTEEEKNAVIWIQKTLGEEAKRFTI

Drer075_2 DTDVKIADAQTEIKKMLKHT--NIDVFLLVIRLDQFTNEKMQAEQQQEEQQEEQ------

Drer076_1 ETD----KTIEKIQQCLSLSSPGPHAFLLVIPIERYTDEQERIAEMILEMFNEDISRYTI

Drer076_2 K-----RVHQAEMK----------------------EQENSANIELIK------------

Drer077_1 DTDSREELLKQEISKCINMTAPGPHAIILVIKLDTFTEEEKLSVEKIRAVFGEAADKHTI

Drer078_1 DTKEKCTVIEEKIKLCISLSAPGPHVFLIVLQLGRFTEEEKKTMEQIQNIFGERASKYTM

Drer079_1 STNLPKEQLAKELARSVYLSASGVHAFLFVVPYGRFTKQEEDILKRVRKVFGKDVLKHVI

Drer080_1 HMFMSERQVKAEIEKSLEMSAPGPHVFLLIIRLGRFTEEEKNAVIWIQKTLGEEAKRFTI

Drer081_1 NCDEPNAHVKQEVLRALNLCDPGPHAILLVIQLGRFTEQERRVMDTLQKILCSNVNLYTT

Drer081_2 DPSFTIEEIVSRIKLSIPLSAPGPHVFLLVLRPGRFTKEDKDTVDIFLKIFGEDAGKHFM

Drer082_1 CTNLPKEQLSKELARSVYLSASGVHAFLFVVPYGRFTEQEEDILKQMQKAFGKDVLKHVI

Drer083_1 NTQISDDQITQTVRECVRLSDPGPHVVLLLLQHQQCSAEDQERVEKLQDSFSERLLQHTL

Drer083_2 NTGLSEEEVMRQTLRCVSLCHPGVHAFLLIIP-DPLNNEDRAEMEEIQKIFSSRINKHIM

Drer083_3 DTELSNEEIQREIRHCISMILPGPHVFLLLIPLGRFTKEEEASVKIIQETFGEHSLMFTM

Drer083_4 M------DRVQEA------------------------EEEKERMKMMMEEEEQ-------

Drer084_1 STNLPKEQLAKELARSVYLSAPGVHAFLFVVPYGKFTEQEEDILKRMRKVFGEDVLEHVI

Drer085_1 DTAVDEETIKSEIIRSVIESSPGPDVFTIVLKVGRYTGHEMEIVDKIVEYCGEDTFNHSV

Drer086_1 HKSVP-ESSMEELK----PSYTGPRVFLLILRDKHLSSQDMEMFTELKKKFGEKMVENTI

Drer086_2 NEDLTDQ--EDQIKRCKDLTQPGPDVYLLVMQLGRFTEGEREVLPNLKKVFGEEVTSKIV

Drer087_1 CTKLSKEQLAFEFARSVYLSASGVHAFLFVVPFDRFTEQEEEILNKVEQVFGKKVLKHVI

Drer088_1 DTAIDEETIKSEIIRSVIESSPGPDVFTIVLKVGRHTEQEMEIVDKIVECSGEDTFNHSV

Drer089_1 DTAIDEETIKSEIIRSVIESSPGPDVFTIVLKVGRHTEQEMEIVDKIVECSGEDTFNHSV

Drer090_1 DIEMSEEEIRQMINKILQICSSGLCVFLLVIKADRFTEEDRKTVEKIEKILGENNQNNTW

Drer090_2 DTKMKPEELMMEIARSVYISSPGPHAFLIVFHVNRFTEQEEQIPQMIELMFGEEVLKYSI

Drer090_3 K------QRLEEIK----------------------RVREKTEERIRAEIEA--------

Drer091_1 CTKLSKEQLAKEFARSVKLSAPGVHAFLFVVPFDRFTEQEEDILNKVEKVYGKDVLKHLI

Drer092_1 YTHTSPDELL----RSVCLSSPGPHAFLIVFPVNRFTEQDERIPQMIELMFGEGVLNYCI

Drer093_1 DTRLDENVIKSEIIKSTIECAPAVDALVIVLKVERYTRQETEILDKIVECCGEETFKHSV

Drer094_1 NTNMSEEEIQQMINKVLQKCSSGLCVFLLVIKADRFTEEERKTVEMIEKILGENNQKDIW

Drer094_2 DTQMKPEELMMEIARSVYISSPGPHAFLIVFPLNRFTEQEQLIPQMIEIIFGQEVLKYSI

Drer095_1 HTHMNNNELMMEIRRSVYISSPGPHAFLIVLRANRFTELEQQTLQKIELMFGKDVLNYCI

Drer096_1 HTHISDDQITQTVRECVSLSDPGPHVVLLLLQHQQCSAEDQERVEKLQDSFSERLLQHTL

Drer096_2 Q------EKVRTMKFEMETQ----------------QSQEKERMKMMKEGTRR-------

Drer096_3 DTHMNNNELMMEIGRSVYISSPGPHAFLIVLRADRFTELEQQTLQKIELIFGKDVLNYCI

Drer097_1 IIGSTEKEVTREILKSISLYSPGPHVFLLVMPVGNLTNDDKSMHKLIESMFGERIWQYTI

Drer098_1 HTHISDDQITQTVRECVSLSDPGPHVVLLLLQHQQCSAEDQERVEKLQDSFSERLLQHTL

Drer098_2 Q------EKVRTMKFEMETQ----------------QSQEKERMKMMKEGTRR-------

Drer098_3 DTHMNNNELMMEIGRSVYISSPGPHAFLIVLRADRFTELEQQTLQKIELIFGKDVLNYCI

Drer099_1 NVRWYQNKLKQDIEKYLEKCAPGPNVFLLVMRPNRHTDEDANTVKWIQENFGEEAVRYTM

Drer100_1 HRHKRPEQLMMEISRCVCLSYPGPHAFLIVFTIYGVTKYELQFLQKIEQMFGEEVLKYSI

Hmag001_1 DHQL---QIATDLKKFLETSKGGVHCIIIVLKYGRITREERINLEIIEKLFDKSWINNCI

Hmag002_1 DTEWDDKQILNMIKSRISANCKHVDAVFIVFRCDRLFKEHVENIKKMLDWLGYSNVKRFR

Hsap001_1 DTEVPNAETSKEIIRCILLTSPGPHALLLVVPLGRYTEEEHKATEKILKMFGERARSFMI

Hsap002_1 DTKESLDTTCKEISRCIISSCPGPHAIVLVLLLGRYTEEEQKTVALIKAVFGKSAMKHMV

Hsap002_2 T-----DQLNEEIKLVEEDKHK--------------SEEEKEKEIKLLKLKYDEKI----

Hsap003_1 ESQADTQELYKNIGDCYLLSAPGPHVLLLVIQLGRFTAQDTVAIRKVKEVFGTGAMRHVV

Hsap004_1 SSQVSKTPGCEERGHCYLLSAPGPHALLLVTQLGRFTAQDQQAVRQVRDMFGEDVLKWMV

Hsap005_1 SPQVSP-EVADAICQAIVLSAPGPHAVLLVTQLGRFTDEDQQVVRRLQEVFGVGVLGHTI

Hsap006_1 SWKDHCEALYKEVQRCYLLSAPGPHVLLLVTQLGRYTSQDQQAAQRVKEIFGEDAMGHTI

Hsap007_1 SSIACAEDKQRNIQHCLELSAPSLHALLLVIAIGHFTREDEETAKGIQQVFGAEARRHII

Hsap007_2 SLKNIDSEVRKHI-------CTGPHAFLLVTPLGFYTKNDEAVLSTIQNNFGEKFFEYMI

Hsap007_3 DVEKDPSRLEEEVKRCLSCCEKGDTFFVLVFQLGRFTEEDKTAVAKLEAIFGADFTKYAI

Hsap008_1 HSRLSKKYVKQEVKEALAHHQGGLHLALLVQRADPFGQEVTDPVQMIQELLGHAWMNYTA

Lgig001_1 DSKIPNEETVKEISKCLALTAPGPHAFLFVLRIGRFTKEEVDTLEILRNLFGENVYKYVI

Lgig002_1 DTAKPNGETIEELIKCIGLLAPGPHVFIFVLSVERFTREDIESMEILKQMLGDGTMHHII

Lgig003_1 DSKIPNKETVKEISKCLALTAPGPHAFLFVLRIGRFTKEEVDTLELLRNLFGENVHKYVI

Lgig004_1 DSKIPNEETVKEISKCLALTAPGPHAFLFVLRIGRFTKEEVDTLEILRNLFGENVHKYVI

Lgig005_1 DTSVPNGQTINEIIKCISLSTPGPHAILFVLNLRRFTEEEIKTVDILKRLFGTEAMKYIV

Lgig006_1 DTDKPKGETIEELIKSIAVVAPGPHVFIFVLTVARFTEEDIKSVDIMKKILGEGTMHHII

Lgig007_1 DTNVANDETITEIVKCVALAAPGPNLFLFVLSLKRLTKEDIDSIELLKSTFGKDVLKYAA

Lgig008_1 DTNIANDETITEIVKCVALAAPGPNLFLFVLSLKRLTKEDIDSIELLKSTFGKDVLKYAA

Lgig009_1 DNRMSEKETCGELARCISLSLPGPHFFVIVIQIGRFTDEELKVVEMLKKEFGVELMNFAL

Lgig010_1 NSHLTEEETQKEIDRGMELLAPGPHIIIYVQSVRQFSGDEKESLEFIKKLFGDNVINHMI

Lgig011_1 NSHLTEEETQKEIDRGMELLAPGPHIIIYVQSVRCLTSSDVEILQTMTEKM-EFYSNHFF

Lgig011_2 STLRSDVDVAKEIMNTIKVNRLLIHIYILVLSADRFSGDEKESLEFIKKLFGENVIKNLI

Lgig012_1 DTSTNNMGTAKILVRTAKENRPGPHIFIFVLSIGRFTIQELETVELLKDLFGNHL-----

Lgig013_1 DGNDIDAEIKKSIKRCVELAHPGPQVFLFVFSCSRFCENDTCVIDWFKEFFGEEIYKHAV

Lgig014_1 QRRTTFESSCYFCMEPIKLTKPGPHIFMFVLSIGRFTQEECETIEFLKNIFGSHVTDFLI

Lgig015_1 NIYTPNEITIKKIVKSKTICAPGPHVIVFVLDLKRFIIEEIETMEILKKLFGTDAMKHII

Lgig016_1 NIRLTEEETQKEIDRGMELLAPGPHCVIFVFSLSRVTGDDIKRIQNFQKVFDMYLNKHVL

Lgig017_1 NIRLTEEETQKEIDRGMELLAPGPHCVIFVFSLSRVTGDDIKRIENFQSVFDMYLNKHVL

Lgig018_1 NIRLTEEETQKEIDRGMELLAPGPHCVIFVFSLSRVTGDDIKRIENFQRVFDMYLNKHAL

Lgig019_1 STLRSDVDVAKEIMNTIKVNRLLTNVYILVLSADRFSGDEKESLEFIKKLFGENVINHMI

Lgig020_1 SKYRSDVDVGKEIMETIRVNNLSINVYILVLSADKELDSRSESVEFIKKLFGGNVVNHMI

Lgig021_1 SKYRSDVDVGKEIMETIRVNNLSINVYILVLGADKVTQSSIDSVEFIKKLFGEKVINNMI

Lgig022_1 SSLRSDVDTANDIISTIKRLTS-IHVCILVLSVDRFSGSEDKAWEFVRCVFGGEISDQLM

Lgig023_1 NIRLTEEETQKEIDRGMELLAPGPHCVIFVFSLSRVTGDDIKRIENFQKVFDMYLNKHVL

Mbre001_1 D-----P-----------------------------------------------------

Nvec001_1 D---GDEVYLNRIKPVLRE----IDVMLYCIKMDRFIENEVNAIRAISSLDR-DIWRRTA

Oluc001_1 ASATDNSTIMNDAKKFTKQH--KPDIVLYFDRLDSRDAADLPLLKQITNTFGQAIWFNAI

Oluc002_1 EGDS-SARGLMALRAALNGR--KVDAFVFTDRLDRVDNADKAIFTSLAENFGAELWERTV

Ppat001_1 DPNLPPHYIGKEIMKCLDLAKDGVHALLMVLSVRRFTDEEIAAVESLQTIFGEKVVNYMV

Ppat001_2 NYE----EQVKQLREMVESK----------IRLNRLERLSREQSREAAEERARSRA----

Ppat002_1 EAGR--DAALNSIRRYLLGK--TINVVLYVDRLDREDKVDVKISRAISQAFGPQIWPHVI

Ppat003_1 PSVADNERIMGQVKKHIKKA--SPDIVLYFDRLDSRDFGDLPLLKTITDLFGAAVWFNAI

Ppat004_1 ESKGT-EEAIQKLKTFVSKIGGIHSYYIYVVQ-YRLDYFDSILWNFFTRVLPNAI-HNLC

Ppat005_1 PSVADNERIMGQVKKYIKKA--SPDIVLYFDRLDSRDFGDLPLLRTITDLFGAAVWFNAI

Ppat006_1 PSTADNKNIMRQVKKYIKKV--SPDIVLYFDRMDTRDSGDVPLLRTITDVFGAAVWFNAT

Ppat007_1 EGGC-NDQALDIIKRFLLNK--TIDVVLYVDRLDRVDNLDKQVIRALARSFGPNFWRIAI

Ppat008_1 ESTGS-EDVENMILEFLKNVKNNYSHIIYVKSGARFTIMDEKIWAAFTSVFEGAE-ESFI

Ppat009_1 EGGC-NDQALDIIKRFLLSK--TIDVVLYVDRLDRVDNLDRQVIRGLARSFGPNFWRLAV

Ppat010_1 ESSGS-ENAESLVLDFLKKVKSSYSHIIYVKSGVRFDIVDEKIWQTFKYVIAGAE-DSFI

Skow001_1 DTRITPTMLATETATCMSIAGNGLDAIILTLNADRLTEEHLNSVKFLRALFGEDMMKHVV

Skow001_2 DTDQAEKTIITEITKCVGVAGEGLDAFILVLNADRFTKEHADSVKIFRKTFGDDMMKYLI

Skow001_3 DTRQAEKTIITEISKCVGVAGEGLDAFILVLNADRFTKEHADSIKIFHKTFGDEMMKYLI

Skow002_1 DTGQ--EKTITEITKCVGVAGEGVDAFILVINADRFTKEHVDSVKIFRETFGDDMMKYLI

Skow003_1 DTSLTNEDMAKEIAKCVGIAGNGLDAIILTLNADRLTEEHIKSIKLLRALFGDDMMKYVT

Skow004_1 DTSLTNEDMAKEIAKCVGIAGSGLDAIILTLNADRLTEEHINSIKLLRALFGEDMMKYVT

Skow005_1 DTRLTNENLAKDIAKCVGIAGNGLDAIILTLNADRLTEEHINSIKLLRALFGDDMMKYVT

Skow006_1 LTDDTNGLTSKEAA------ME--------------------------------------

Amil001_1 VLFTFADGQKA---------------------------------SAMQAAGILED-----

Amil002_1 LVLTYCDGESANEFRQSFED--EANDIAFMRKGYMVGFPDR--PDIKEYAKMLQKGKEI-

Amil003_1 LVITGCESLDGRSLKDELRTEIITRRVVLMHKGFPVGFPNK--PVTDSDTNHLKETRQH-

Amil004_1 LVFTFCDGETANKFLDTFKTA--ARDIVFMKQGYMVAFPHK--QDITKQAKMLQKGNEM-

Amil005_1 TVLTYDDEVAE---------KRLIKAAMEVTGSRRNVFLI-------------------A

Amil006_1 LVLTHGDEAEYSTVEEYLKDDDWLKNFIDDLKDRVVLLNCRLKPYKKQLCKLIEMIDKIK

Atha011_1 VVFTGGDVLEK-TLEDYLGRPTFIKEVMRMSSNRKVVIDNKTHKKAEQVHKLLSLVDDIR

Atha012_1 VVFTCGDMLEN-TLEDYLSNPEFLKNVLRLCGGRRVVFDNRTKVKAKQVQQLLVHVAAIE

Atha013_1 VVFTGGDVLEG-TLEDYLGDPDFLKRVLILCGQRMILFDNKTKKKTKQVHELLKLIDLVR

Atha014_1 VVFTGGDELEE-TLEEYLADPEFLKEILGICDNRLVLFNNKTTKKAEQVQKLLSLVESVV

Atha015_1 VVFTNEDSLEG-TFEEYL-EPDF-KEILEPCNDRKVLFRNRSNQKAKQVQELLNYVEEIA

Atha016_1 VVFTGGDELEE-TLEEYLADPEFLKEILGICDNRLVLFNNKTTKKAEQVQKLLSLVESVV

Atha017_1 VLFTGGDELEN-TLDDYLSKPEFLKTVLRLCGGRRILFDNRTTKKVKQVQELLAHVAAIE

Atha018_1 VVFTNEDALEG-TLDDYL-EPEF-QEILEECDDRKVLFDNSYNKKDRQVHDLLNLVEQIS

Atha019_1 VVFTGGDDLED-TLDDYFRAPEFLTKVLRLCGGRKVLFDNKSKKKVEQVKQLLARVENVG

Atha020_1 VLFTGGDELEN-TLDDYFHQPYFLKTVLGLCDDRKVMFNNMTKKKVEQVQQFLALVAKVE

Atha021_1 VLFTGGDVLEN-TLDDYLSRPEFLKTVLRLCGGRRVLFNNKTTKKIEQVKQLLAHVEAIE

Atha022_1 FLLIDGEKFE--EFEDYFPEPEFLMRVLRFCNGRKVLFNNMTNVKAEQVNQVMAHVAAIS

Atha023_1 VVFTGGDELEN-TLDDYLREPEFLTRVLKLCGGRKVLFNNKTKKRNKQLNQLLAHVTDIR

Atha024_1 VVFTNEDSLIN-TINEYL-EPDF-KEILAACNNRMVLFENRLRKKAKQVQKLLDLVEEVE

Atha024_2 ------------------------------------------------------------

Atha025_1 VVFTGGDKLEE-TLDDYFSEPKFLTGVLRLCGGRKVVFNNMTKKNAKQVKQLLAHVEAIE

Atha026_1 LVLTHAQFSPGLNYNHFVSKSNALLKVIQTGAQKKQDLQG-NSKPCGTSWIPNL------

Atha027_1 LVLTHAQFSPELSYETFSSKSDSLLKTIRAGSKRKQEFED-NSKPNGEAWIPNLV-----

Atha028_1 ------------------------------------------------------------

Atha029_1 LVMTHSAAT-GVNYESYVGQMDVVQHYIHQAVSTKL------------------------

Atha030_1 LVMTHSAAT-GVNYESYVGQMDVVQHYIHQAVSTKL------------------------

Atha031_1 VGLTHAASAPGSSYDMFVTQSHVIQQAIRQAAGMRLMNPVNGQ-----------------

Atha032_1 VGLTHAASVPGSSYDMFVTQSHVIQQAIRQAAGMRLMNPVNGQ-----------------

Atha033_1 S-----------------------------------------------------------

Atha034_2 ------------------------------------------------------------

Atha035_1 VTLTHAASAPGLSYDVFVAQSHIVQQSIGQAVG---------------------------

Bflo001_1 IVITCKDVIVQNDITKYIQTPETFKTLLKECKGRYVAIDNQTKVNRMQLKELFTLVDRMV

Bflo002_1 IVLTNKDKIVKKDVTKFIQTPQTLQNLLKECNGRYVAFDNKAKVKRVQLTELVQLLDEVV

Bflo003_1 ILFTYGDDFESGTLEDCVFAPKWFKELLKHVKDRYVIFDNYTDKKKSQRSKLLQKILEVM

Bflo004_1 IVLSHKDEIDDKDVKKYIETPEKFRELLKDCGQRYVAFNNVTELKRMQVAELVKLVEDTI

Bflo005_1 VIFTHGD-----------------------------------------------------

Bflo006_1 ILYTHGDDFEA--LKDLINDPKWFKGLLRQVKNRYLIFDNRTNTKDRQRHRLLDMIRSVM

Bflo007_1 ILYTNGDEFEAGTLESCVHSPQWFKELLKLVKNRYLIFDNYTKKKESQRCKLLQTILEVM

Bflo008_1 LLITAKDEL--TSEEEYLKTPDDLKNVLKKCGNRCVFFNNVSKILRMQLVNMIRLVDTIT

Bflo009_1 VLITGKDS---KSKEEYLASPQTLSDILKKCQERCIFFDNVTMVRRKQLAKLITMAQEAV

Bflo010_1 VVITHGDQVQLTSIEDYVSEWGGLPKLMKKVDNRYVVFNNR--EKKNQMKRLMDLVEQVS

Bflo011_1 ILVTGKDNLR-GSESEFLSAPESLRTILKQCGERCVFFDNTTRLKRQQLVKLIQMIDEIV

Bflo012_1 LLITGNDEL--MSEVDYLRPSQALQDLLKKCGNRCVFFNNISKILRMQLVKLIRLVDDIV

Bflo013_1 MVVTGMDVIDEVNKQDYLTAPREFLDVLKECGTRCVFFDNKTKLRRTQLWKLVTMVEKTV

Bflo014_1 IVITGKDDIDIRDVKTYLRNPPGLQEVLKLCKHRVVFFNNKTRIQRMQLAKLIRMIDGLV

Ddis001_1 VVLTYANSVLS-CYDGFDEEDVGPWKKHYEARAQFR------------------------

Drer001_1 ILFTRADDLEDRTLEDFIEEAPELREVIEACSGRFHMLNNREKRDRAQVDELLRKIVVMI

Drer002_1 VLTTDEETL---------RFNKAVQNVIKDCEGRHLRFDT----NPQSHTKLFIKIEKIL

Drer002_2 ILIQQDSEL--HA-------SEETQTVIQSLSGRHHFIN-----PNTPVSTLMEKIDQMV

Drer002_3 VLFTRGDDLRNKTIGDFLNTDSALKNLTETCGNRVHVFNNNQTKDPTQVSDLLMKIEKMV

Drer002_4 VLFTEADILNNKTIEQYEKANDELKNMISDCGNRYLDFNNTETQDQTQVTRLFNMIEEIR

Drer003_1 VLTTDEETR--------TKFNSAVYSLIKVCNGRHVKFDS----TLDYYSRLLKMIEKIL

Drer003_2 VLFITEHTV--NSPSEF---SEESQRIVSLYGCWYRVMGL---RNTEQISELLQYTDNM-

Drer003_3 ILIQQDSEL--HA-------SEETQAVIQSFGEQHYFIS-----PNTPVSTLMEKIEQMV

Drer003_4 VLFTRGDDLDSKDIQHYLSPGSTLMKLIEACGNRYHVFNN-RSGDQKQVSELLEKINNMV

Drer003_5 VLFTKADTLKNQTITQYVEKSKTLKSLISACGDRFLAFNNAETQDQTQVTELFNMIEEMM

Drer004_1 VLFTRGDDLKDQSIEDYVKRSAELQKLIRDCGNRFLVFNNREKQDKTQVMKLLKMIEEVK

Drer005_1 VLTEEEDIN-----------NDCIHQLIQECGGGHLQFDQ----KSECQSEILKRIKKIL

Drer005_2 VLIIQKSEH--RK-------NEAMKAVIECFGGRHHYLE-----LNTQVSMLVTKLEQMV

Drer005_3 VLFTRGDGLKNKTIEEYLEPGSSLMNLIEQCGNRYHVFNNNETEDRTQVTKLLQKINDMV

Drer006_1 VLFTRREKMS-KEWMLFML-SAEFQELVGHFRGQYHAISSTSEINQSHIAELLKKIDDMI

Drer006_2 ILFTRGDQLKNTTIEEFLTKNKCIKEIVDQCKGGYHVFDNTDQTNRAQVTELIEKIDSVV

Drer007_1 ALTTDKETP---SMFSAFLTVQTIHQLIKECGGGHLQLDE------RNKADIIQLIDKIL

Drer007_2 LLFMTKQTV--EPVTDF---HEDSKRLCSQYGGRYKVMGL---KNSKQILELFSYIENL-

Drer007_3 VLFTRADELKDQSIEDYVKRSAELQKLIRDCGNRFLAFNNREKQDKTQVMKLLKMIEQVN

Drer008_1 VLTTDEESY--------GMINNAVNLLIKECGGGHLQLDE----NPEWRSDLPKRFEKIL

Drer008_2 LLFTTELTD--EFATEF---YSDCQKLISLCGGQYRVIGF---EDSKQIPELLEYIVNM-

Drer008_3 VLFTRGDDLEDQSIEDYVRESAELQKLIRDCGNRFLAFNNRENQDKTQVMKLLKMIEEVK

Drer009_1 VLTTDEESY--------GMINNAVNLLIKECGGGHLQLDE----NPEWRSDLPKRFEKIL

Drer009_2 LLFTTELTD--GFAMEF---YSDCQKLISLCGGQYRVIGF---EDSKQIPELLEYIVNM-

Drer009_3 VLFTRGDDLEDQSIEDYVRESAELQKLIRDCGNRFLAFNNRENQDKTQVMKLLKMIEEVK

Drer010_1 VLFTHGDLLKGQTIDQYVREHIELRRVIQSCGGRYAVFNNTMK-DRTQVKTLIDKIDQMV

Drer011_1 IIMTDKETH-----------NTAISQLINVCRGRHLLLEE----KPDWPAEIFNKIDMML

Drer011_2 VIFISDGTI--KPVRDF---NPEFQRLISHCGGLYCVMGL---ARSRQVPELLEYIEKM-

Drer011_3 VLFTRGDDLKDQSIEDYVKRSADLKKLIRDCGNRFLVFNNNEQQDKTQVIRLLKIIEEVK

Drer012_1 LLFTTELPV--EFVTDF---YTDSQQLISLCGGQYSVIGL---EDSTQIPELLEYIEKM-

Drer012_2 VLFTRGDELKDQSIEDYLKRFAELQKLIRDCGNRFLVFNNREKQDRTQVMKLLKMIEEVK

Drer013_1 VLFITEHTV--------NSPSEESQRITMRARHLGAKLSE----KDGEIRELQQKINTL-

Drer013_2 ILIQQDSEL--HA-------SEETQAVIQSFGEQHYFIS-----PNTPVSTLMEKIEQMV

Drer013_3 VLFTRGDDLDSKDIQHYLSPGSTLMKLIEACGNRYHVFNN-RSGDQKQVSELLEKINNMV

Drer014_1 VLFTRGDDLGDESIEDYVKRSADLKKLIRDCGNRFLAFNNREKQDKTQVRKLLKMIKEVR

Drer015_1 VLFTKGDNLDD-SIEAYIKDDSRVKQLIHDCGGRFHVFNNKQK-DPGQVVGLLKKIDKMM

Drer015_2 ------------------------------------------------------------

Drer016_1 ALTTDKETP---SMFSAFLTVQTIHQLMKECGGGHLQLDE------RNKADIIQLIDKIL

Drer016_2 LLFMTKQTV--EPVTDF---HEGPKRLCSQYGGRYKVMGL---ENAKRILELLNYIENL-

Drer016_3 VLFTRGDDL-NESINDYVSKCAELQKLIRDCENRFLAFNNREKQDKTQVMKLLKMIEEVK

Drer017_1 ALTTDKETP---SMFSAFLTVQTIHQLMKECGGGHLQLDE------RNKANIIQLIDKML

Drer017_2 LMFMTKQTV--EPVTDF---HEDPKRLCSQYGGRYKVMGL---ENAKRILELLNYIENL-

Drer017_3 VLFTRGDDL-NESINDYVSKCAELQKLIRDCENRFLAFNNREKQDKTQVMKLLKMIEEVK

Drer018_1 VLTTDEEST----------INNAVNLLIKECGGGHLQLDE----KPEWHSDLTKRFEKIL

Drer018_2 VIFISDGTI--KPVTNF---SPECQKLISHCGGLYCVMGL---SSSRQIPELLEYIEKT-

Drer018_3 VLFTRGDELKGQPIEDYVTKNPDLKKLISDCGNRFLAFNNNEKQDKTQVIKLLKLIEEVK

Drer019_1 VLFTRGDDLEGSRIEDYIEGDRSLQNLIHQCGNRYHVFNNKETEDQTQVSELLEKIDRMV

Drer020_1 ILTTDDEKD--------STQNKRIQQLSAECGGRLQLLNT-------LRSDIFRKVDEVH

Drer020_2 VLFTRGVDLKGTSIEDFIEGNRSLENLLHQCGNRYHVFNNDETNDKTQVSELLEKIDRMV

Drer020_3 --------------------------------------------MKKETERKRQE-----

Drer021_1 VVSMHEDQG-------YMMSNREMESLQQTWKRYHHLHRN----DQSQIQKLLESVKKMV

Drer021_2 VLFTKGDNLTDLSIEDYIEDDSHVKQLIHDCGGRFHVFNNKQK-DPAQVVSLLKKIDKMM

Drer022_1 VVFMLEEQM------EYVITSRSEKPLLQISKCHHNLQRN----DQSQVQNLLEIIEEMV

Drer022_2 VLFTKGDNLDD-SIEDYIEDDSHVKQLIHDCGGRFHVFNNKQK-DLAQVVGLLKKIDKMM

Drer023_1 VVFMLEAQM------EYVITSRSEKPLLQISKCHHNLQRN----DQSQVQNLLEIIEEMV

Drer023_2 VLFTKGDNLDD-SIEDYIEDDSHVKQLIHDCGGRFHVFNNKQK-DLAQVVGLLKKIDKMM

Drer024_1 VLFTRGDDLKNKSLDQCLKPGSVIRKLLETCRNRFHVFNNNQPEDRTQVSELLEKIDNMV

Drer025_1 ALFTHASQVKDQDFGAYVSSDERLQAFVRRCGGDCFWIDN-DKKDPAHVMQLLDKIEEMV

Drer026_1 ILFTHEDELEDPSNEQYLQNHADLQRLVTECEGRFHCFDNNCK-SGDQVNDLLQKIERLV

Drer026_2 IIFTHKDQLEEKTIEQFLQDDPGLKELVESCGKRFLCLDNK-SASFPQFKDLISKVEEMV

Drer027_1 VLFTRGDDLKKKTIEEYLAPGSALMSLIEQCGNRYHVFNNNETGDHMQVTELLEKIDGMV

Drer028_1 ILFTRADELEN-TEQEFLNINGGLKKLVQKYDQRYHMFNNKKKGPKGEKTFL--------

Drer028_2 IVNLKMNNQ--IT-------SSEIS--IQTFGARQFVLE-----NSSQVPDLLQDVENMV

Drer028_3 VLFTRGDDLGGTSIKDFIEGDENLQNLIHQCGNRYHVFRNKETEDQVQVSELLEKIDRMV

Drer028_4 --------------------------------------------IKKETERKRQE-----

Drer029_1 VLFTRGDNLKNKTLDQCLKPGSVVRKLLETCGNRFHVFNNNQPEDRTQVSELLEKIDNMV

Drer029_2 --------------------------------------------QNQEEESKRR------

Drer030_1 VLFTRGDFLKDKTLEECLKPGSVVRKLLETCGNRFHVFNNNEPEDRTQVSDLLEKIDNMV

Drer031_1 VLFTRGDFLKDKTLEECLKPGSVVRKLLETCGNRFHVFNNNEPEDRTQVSELLEKIDNMV

Drer032_1 ALFTHGDQLKGKNIHRFIRDSPKLLRFIKTCGGRFHVFNNEDQ-NPEQVLKLFDDVDKIV

Drer033_1 ALFTHGDRLEGKNIHTFVRDSPKLLSFIRTCDGRYHVFNNKEE-NPEQVIQLLEQIDKMV

Drer034_1 VVFMLEEQM------EYVITSRSVKSLLQISKCQHHLQRN----DQSHVQILLKIIEKMV

Drer034_2 VLFTKGDNLDD-TIEDYIKDDSHVKQLIHDCGGRFHVFNNKQK-DPAQVVGLLKKIDKMM

Drer035_1 VLFTRGDELKGTSIEDFIEGDRSLQNLIHQCKSRYHVFSNNEVKDLTQVSELLEKIDRMV

Drer036_1 VLFTRGDNLKYQSIEDYMKRSAELQKLIRDCGNRFLAFNNREKLDKTQVMKLLDMIQEVR

Drer037_1 VLFTRGDNLKNKTLDQCLKPGSVVRKLLETCGNRFHVFNNNQPEDRTQVSELLEKIDNMV

Drer038_1 ILFTHADALEDQLLYGYISQSGDLWDLLYECGARYHSFNNKDMNDRSQVAELMEKIEKML

Drer039_1 VLFTKGDSLKNTTIDQCLRPGSVVRKLIEACGNRFHVFNNNEPEDQRQVSDLLEKIDNMV

Drer040_1 ALFTHGDRLEGKNIHTFVRDSPKLLSFIRTCKGRYHVFNNKEK-NPEQVIQLLEQIDKMV

Drer041_1 ILFTRGDQLNGESVEEFIKESEALRSVVQQCGDRYHVFNNRDVNNREQVEDLLQKIDSMI

Drer042_1 IIFTRGDELED-TLEEFIEEAEDLKEVVERFDYRYFIFNN----SPEQVHNLIEKIKLIK

Drer042_2 PVFTHGDQLEGESVEDLITQNETLSKIVQRCGGVYHIMNNKDPRNRKQVNDLLQKIDRII

Drer043_1 ILFTRGDELKDQSIEDYVTKNPELQKLIRDCGNRFLAFNNNEKQDKTQVMKLLKMIEEVK

Drer043_2 ILTTDDEPE-------------------LIAGQ---------------------------

Drer044_1 LIFTHADRLNGGSIQEFILNKQKIQELVEKFGSRFVAFNNKNPENREQVTRLLQKVDELM

Drer044_2 ------------------------------------------------------------

Drer045_1 ALTTDKETP---SMFSAFLTVQTVHQLIKECGGGHLQLEE------GNKADIIQLIDKIL

Drer045_2 LMFMTKQTV--EPVTDF---HEGPKRLCSQYGGRYKVMGL---ENAKRILELLNYIENL-

Drer045_3 VLFTRGDDL-NESINDYVSKCAELQKLIRDCENRFLAFNNREKQDKTQVMKLLKMIEEVK

Drer046_1 VLFTRGDDLKNKSIEEFLKPGSPLMNLIEACGHRYHVFNNNQPEERTQVSDLLEKIDNMV

Drer046_2 ---------------------------------------------KQEKERKRRE-----

Drer046_3 ILFTGGDELEN-TIQEFIEETEELKTLVQKYEHRYHLFNNKRKMTSVQVKALFTKI--LK

Drer046_4 ILFTHGDLLDGEPVEKLIEENSGLRSVVQQCGGRYHVFNNRDEENREQVEDLLQKTELMI

Drer047_1 ILFTHADQLKGKPLEDYISQSSDLQKVIDICDGRYHSFNNQEKNNQSQVTELLKKIDAML

Drer048_1 ILIMQNSEH--QA-------SEETQAVIQSFGGRHHYFS-----PETQVSTLMENIEQML

Drer048_2 VLFTRGDDLKNKSIEEFLKPGSPLMNLIEACGHRYHVFNNNQPEERTQVSDLLEKIDNMV

Drer048_3 ILFTRGDELEN-TIQEFIEEIEELKTLVQKYEHRYHLFNNKKMRTSEQVKMLFTKI--LK

Drer048_4 ILFTHGDLLDGEPLEKRIEENCRLRSLVQQCGGRYHVFNNRDEENREQVEDLLQKIDSMI

Drer049_1 VLFTRGDDLKKKTIEGFLKPGSVVRKLIESCRNRYHVFNNNQPEDRTQVSDLLEKIDNMV

Drer050_1 ILFTYGDQLDGESVEEQIEENCRLRSVAQQCGGRYHVFNNEDVNNREQVEDLLQKIDSMV

Drer051_1 ILIKQNSDH--QA-------NEETQTVIQSFGGRHQYFN-----PETQVSTLMENIEKML

Drer051_2 VLFTRGDDLKKKTIEGFLKPGSVVRKLIESCRNRYHVFNNNQPEDRTQVSDLLEKIDNMV

Drer052_1 VLSTQESTE-----------NEILQKIIQTCSNRHFSLQ-----RSSSADDLLEAFEDIE

Drer052_2 TLIMQNSEH--QA-------NDETQAVTESFGGRHHIFG-----PTTQVSMLMENIEQML

Drer052_3 VLFTRGDYLRNKTIEQCLKPGSPLMKLIEACGHRFHVFNNNETEDRTQVADLLEKIDNML

Drer052_4 -------------------------------------------------DEIVSK-----

Drer053_1 ILFTHADALERQTLHQYICESADLWALLSQCGRRYHSFNNKDEENRSQVTELMEMIEKMV

Drer054_1 ILFTRGDQLDGKSVEKLIEDSSGLRSLVQQCGGRYKVFNNRDVNNREQVEDLLQKIDSMI

Drer055_1 VLFTRGDDLKNKSIEEFLKPGSPLMNLIEACGHRYHVFNNNQPEERTQVSDLLEKIDNMV

Drer055_2 ---------------------------------------------KQEKERKRRE-----

Drer055_3 ILFTGGDELEN-TIQEFIEETEELKTLVQKYEHRYHLFNNKRKMTSVQVKALFTKI--LK

Drer055_4 ILFTHGDLLDGEPVEKLIEENSGLRSVVQQCGGRYHVFNNRDEENREQVEDLLQKTELMI

Drer055_5 VLFTRGDFLTNKSIEEFLKPGSPLMNLIEACGHRYHVFNNTQPEERTQVSDLLEKIDNMV

Drer056_1 VLFTRGDYMRDQTIEQCLKPGSPLMKLIETCGHRFHVFNNNQTEDQTQVADLLEKIDNMV

Drer056_2 ---------------------------------------------NQEKERKIRE-----

Drer057_1 ILIMQNSEH--QA-------NEETQAVIQRFGGRHHYFN-----PETQVSTLMENIEKML

Drer057_2 VLFTRGDELRNQTIEMFLKPESVVRKLIETCGNRFHVFNNNQPENRTQVSDLLEKIDNMV

Drer058_1 ILIMQNSEH--QA-------NEETQAVIQSFGGRHQYIN-----PETQEATLMENIEKML

Drer058_2 VLFTRGDELRNQTIEMFLKPKSVVRKLIKTCGNRSHVFNNNQPEDRTQVSELLEKIDNMV

Drer059_1 ------------------------------------------------------------

Drer059_2 IIFTHKDQLEEKTIEQFLQDDPNLKELVESCGKRFLCLDNK-SASFPQFKDLISKVEEMV

Drer060_1 ILFTHADALK-RPLDEHIKSSSHLKVLVDEYGSRYHSFNNEDMNDRSQVRKLMDKIDILL

Drer061_1 ILFXXXXXXXXS-----------LQ-----------------------------------

Drer061_2 ILFTRGDQIK-TPIEEFLANNEEMRALAEQCKGGYHVFNNTDEQNRSQVSELLEKIDSML

Drer062_1 ILIMQNSEH--QA-------NEETQAVIQSFGGRHHYFN-----PRTQVSTLMENIEKML

Drer062_2 VLFTRGDFLKNQTIKEFLKPGSVVRQLLETCGNRYHVINNNQPEERTQVSELLEKIDNMV

Drer063_1 ILFTHADQLKGEPLDEYISENNDLKALVSQCGDRYHSFNNEDMINRSQVTELMEKIEKMV

Drer064_1 ILFTHGDELT-STIEEYIRKNEDLKEIIRRCGGRYHVFNNKSMEDRGQVLELLEKVDALV

Drer065_1 VLSTQEPTE-----------NQILQKIIQKCSNRHVSLQ-----TSSSADHLLQAFEDIE

Drer065_2 ILIMQNSEH--QA-------NEETQAVIQSFGGRHHHFS-----PETQVSTLMENIEKML

Drer065_3 ILFTHGDLLDGVSVEKLIEKYSRLRSVVQQCGGRYHVFNNRDENNREQVEDLLQKIDSMI

Drer066_1 VLFTRGDVLGNKTIHQCLKAGPSLMNLIEACGHRFHVFNNNQPEERTQVSDLLEKIDNMV

Drer066_2 ------------------------------------------------------------

Drer067_1 ILIMQNSEH--QA-------SEETQAVIQSFGGRHHHFS-----PETQVSTLMENIEQML

Drer067_2 VLFTRGDFLGNKSIEEFLKPGSPLMNLIEACGHRYHVFNNNQPEERTQVSDLLEKIDNMV

Drer068_1 VLFTRGDELGNKTLEECLKPGSVVRTLLETCGNRFHVFNNNQPEDRTQVSDLLEKIDIMV

Drer069_1 ILFTHGDELT-EDIEITLKERRDLKELVESCGGRYHVFDNTKVHNRKQVLEFLDKVDEML

Drer070_1 VLFTHADQLKRKTLAAYIRESDELQALVDECGGRVHAFHNEDTSDRTQVNKLMEKIEKLV

Drer071_1 VLFTHADQLKRKTLAAYIRESDELQALVDECGGRVHSFHNEDTSDRTQVNKLMEKIEKLV

Drer072_1 ILFTRGDELEN-TLEKFISETEELKTLVQKYDQRYHLFNNKKKRCTGQVKDLLIKILKTK

Drer072_2 ILFTHGDQLDGESVEKLIEENCRLRSVVQQCGGRYHVFNNRDVNNREQVEDLLQKIDSMI

Drer073_1 IFFTHGDQLD-CTIEEYVDVSENLKEIIRRCGGRYHVFNNKDIEDRTQVVDFLEKVDEMV

Drer074_1 ILFTHGDQLD-CTIEEYVDVSENLKEIIRRCGGRYHVFNNKDIEDRTQVVDFLEKVDEMV

Drer074_2 YEAKLREAR-----------------------------------EEAELSRISEE-----

Drer075_1 LLVTGADQLE-RPLEDYLRENLDIQKLVDEYEGRYYVFNNLKE-DREQVSILIEKIPVLV

Drer075_2 ------------------------------------------------------------

Drer076_1 LIFTHADRLNGGSIQEFISRNGKIQELVERFGSRFVAFNNKNSENREQVTRLLQKVDELM

Drer076_2 ------------------------------------------------------------

Drer077_1 ILFTHGDELTDSTIDEYISEGEDLKEIIRRCGGRYHVFNNKDMEDRNQVVDFLEKVEDLI

Drer078_1 VLFTHGENLKQKSIHKFVDESPDLLDFIKTTSGRYLAFDN-NANDPEQVNVLFEQIAQLM

Drer079_1 ILFTYGDECEKKEIQKEI-DNKEVTRVVKRCHD-YHVFNNRGLDDKQQVNDLLLKIDLLV

Drer080_1 LLVTGADQLK-RPLEDYLPENKDLQKLVDEYEGRYYVFNNLQK-YGAQVTELLEKINAIV

Drer081_1 VLFTYGDKLKNKSLDQFIAEDKNLQNLIQKCGSQYHVFNNTDRENKRQVSELFEKLDHQI

Drer081_2 ILFTHGDELKGKTIEEFITGNPDLKMLFEKCQEQYHVFNN-EAKDALQVDQLFEKMQKVI

Drer082_1 LLFTYGDEFDRKNFQSVI-DNEVVRRVIQRCRD-YHVFNNRDLNDRQQVMDLLLKIDSMV

Drer083_1 VLSTQEPTE-----------NQILQKIIQKCSNRHFSLQ-----RSSSADHLLQAFEDIE

Drer083_2 ILIMQNSEH--QA-------NEETQAVIQSFGGRHQYFN-----SETQVSTLMENIEKML

Drer083_3 VLFTKGDSLKNTTIDQCLRPGSVVRKLIEACGNRYHVFNNNQPEDQRQVSELLEKIDNMV

Drer083_4 ---------------------------------------------KQEKERKRRE-----

Drer084_1 ILFTHGDECDRENIQSEI-DNKVVRGVLLKCRD-YHVLDNRSLNDRQQVSELLQKIDSMV

Drer085_1 VLFTHGEQLEGQTIEEFV-KSPKLQELVNKCGGRCHVIDSKYRSNRVQVKKLLETIEEKL

Drer086_1 VMVEKKLA---SSMDKHISFKSQYHAILEECGRRKCVYN-----REIKNVELIRK----L

Drer086_2 ILFTGKEKLRDKSLPDYISGDQELQELVKSCHSRCHAFNNND-KNHHQVKKLLDLIGSM-

Drer087_1 ILFTHGDECDRENIQKEI-DDEVAKRVVQKCRG-YHVFNNRSLNDRQQVSELLKKIDSM-

Drer088_1 VLFTHGENLEGQTIEEFV-KSPKLQELVNKCGGRCHVIDSKYRSNRVQVKKLLETIEEKL

Drer089_1 VLFTHGENLEGQTIEEFV-KSPKLQELVNKCGGRCHVIDSKYRSNRVQVKKLLETIEEKL

Drer090_1 ILFTRGDKLEN-TIEKFIEETEELKTLVQKYEDRYHLFNNNKMKPSEQVKILFNKI--LK

Drer090_2 ILFTHGDLLDGESVEKLIEENFALRSLVQQCGGRYHVFNNK-VNNREQVEDLQQKIDSMI

Drer090_3 ---------------------------------------------KKQRDGVRKN-----

Drer091_1 ILFTHGDEFDIKDIQSEI-ANEVAKRVIQKCRD-YHVLNNKDLNNRQQVSDLLLKIDSMV

Drer092_1 ILFTHGDLLKGKNIEKLIEENCRLRSVVQQCGGRYHVFNNRDVNNREQVEDLLQKIESMI

Drer093_1 VLFTHGEQLEDQTIEEFV-HSPKLKQLVNKCRGRCHVIDNKEKSNRVQVKNLLDTIDEMV

Drer094_1 ILFTRGDELEN-TIQEFIEETEELKTLVQKYEHRYHLFNNKKKMPSEQVKMLITKI--LK

Drer094_2 ILFTHGDQLDGESVEKLIEENSRLRSVVQQCGGRYHVLNNRDENNREQVEDLLQKIDSMI

Drer095_1 ILFTHGDLLDGESIEKLIEENSRLRSVVQQCGGRYHVFNNRDEENREQVEDLLQKIDSMI

Drer096_1 VLSTQEPTE-----------NQILQKIIQKCSNRHFSLQ-----TSSSADHLLQAFEDIE

Drer096_2 --------------------------------------------EEKQLSELYRRDAAV-

Drer096_3 ILFTHGDLL-GKSVEKLIEENSRLRSLVQQCGGRYHVFNNRDEENREQVEDLLQKIDSMI

Drer097_1 IVFTHGDRLEGKAANDVIACDIELREFIHKCSGGFHFFNNKDDTNDESVIDLLKKVETLV

Drer098_1 VLSTQEPTE-----------NQILQKIIQKCSNRHFSLQ-----TSSSADHLLQAFEDIE

Drer098_2 --------------------------------------------EEKQLSELYRR-----

Drer098_3 ILFTHGDLL-GKSVEKLIEENSRLRSLVQQCGGRYHVFNNRDEENREQVEDLLQKIDSMI

Drer099_1 VLFTHVDLLTDESMDDYIRQSLDLKLLIDSCGGKFHTVNNQDRNNPNQVTELLEKIEQLE

Drer100_1 ILFTHGDLLEGGSVEELIEENCTARSVVQQCGGRYHVFNNEDVNNREQVEDLLQKIDSMI

Hmag001_1 VVATFFDGE-----------VNSDTQMIDDAQEAIIGWTHTKVNNRKLRQQCLDK-----

Hmag002_1 FVGTHAPSL-----------------------------------TDEKKEELIKQFEEII

Hsap001_1 LIFTRKDDLGDTNLHDYLREPEDIQDLMDIFGDRYCALNNKATEQEAQRAQLLGLIQRVV

Hsap002_1 ILFTRKEELEGQSFHDFIADDVGLKSIVKECGNRCCAFSNSKKEKESQVQELVELIEKMV

Hsap002_2 --------------------------------------------KNIREEAERNIMLSE-

Hsap003_1 ILFTHKEDLGGQALDDYVANNCSLKDLVRECERRYCAFNNSVEEQRQQQAELLAVIERLG

Hsap004_1 IVFTRKEDLAGGSLHDYVSNNRALRELVAECGGRVCAFDNRATEQEAQVEQLLGMVEGLV

Hsap005_1 LVFTRKEDLAGGSLEDYVRENQALAWLDVTLARRHCGFNNRAQEQEAQLRELMEKVEAIM

Hsap006_1 VLFTHKEDLNGGSLMDYMHDNKALSKLVAACGGRICAFNNRAENQDDQVKELMDCIEDLL

Hsap007_1 IVFTRKDDLGDDLLQDFI-ENKPLKQLVQDYEGRYCIFNNKTNEQITQVLELLRKVESLV

Hsap007_2 ILLTRKEDLGDQDLDTFLRNNKALYGLIQKCKNRYSAFNYRATEEQRQADELLEKIESMV

Hsap007_3 MLFTRKEDLGAGNLEDFMKNNKALRRIFKKCGRRVCAFNNKETAQETQVKALLTKVNDLR

Hsap008_1 ILFTHAEKIEG-TEDKYLHESDTLKTLLNSIQHKYVFQYK---SLNEQRMKILERIMEFI

Lgig001_1 VVFTNKDDLEDVTLVEFVDNPDYLKEFLEQCYQRYVGICNKPGSHEQDVESVIELIIKTI

Lgig002_1 VVFTRKDHLEDTRIEEFIADPGFLKDLLKDVNNRYEFICNRQKSHQQDVENVLSLIKKTV

Lgig003_1 VVFTNKDDLDDVTVEEFVDKPDYLKEFLEQCYQRYVGICNKSCSHEKDVESVIELIIKTI

Lgig004_1 VVFTNKDDLDDVTLEEFVDNPDYLKEFLEQCYQRYVGICNKPGSHEQDVESVIELIIKTI

Lgig005_1 VVFTGKDELDEKTIQDFVKNPDYLRKFVRECNMRYVGIANKPSTHCSDVKDVMTLIYKTM

Lgig006_1 VVFTRKDQLVDVTEEEFMAKPGFLKQLLKDVNNRYEFICNRQKSHQQDVENVLSLIKKTV

Lgig007_1 VVFTCKDELEY--IQEYIEDQQFLKDLIFDCGGRHAAICTKKSNYKKDVKGMLDLITHII

Lgig008_1 VVFTCKDELEY--IQEYIEDQQFLKDLIFDCGGRYAAICTKKSNYKKDVKGVLDLITHTI

Lgig009_1 VVFTHGFKLSGMSIEKYLETPAGLQKVVKESDNRYAVLGDQ--RDRTFQKSVKNIIYLIV

Lgig010_1 IVFTGKDSLEE-------------------------------------------------

Lgig011_1 ILFSGIDELDTQTLPEYVQKNQFAKLLKQQTPVHVCSKGF---NDNTQFNLLIHAITMHY

Lgig011_2 IVFTGKDSLEE-QFPRCL---PQLPYFILDCKDKQEVVEEI---CSEKYNNIPAVIEKE-

Lgig012_1 --------------------------------------------VTESVDSLLQMIKDVF

Lgig013_1 IVFTSKNSKLG-SISKFVRGASELRNLIELCGNRIVAIDNASP-NDVDIRNLISIVVDLV

Lgig014_1 IVFTGKDTLDK-DLEMYINTGDELKSLVSQCQGRIAINNRE---VTKSVDSLLQMITGVH

Lgig015_1 VVITGKDK----------------------------------------------------

Lgig016_1 VVFTGMDEFEG-TLDEYIQK----------------------------------------

Lgig017_1 VVFTGMDNLKG-TLDEYIQ-----------------------------------------

Lgig018_1 VVFTGMDGFEG-TLDEYIQK----------------------------------------

Lgig019_1 IVFTGKDSLEE-QFPRCL---PQLPYFILDCKDKQEVVEEI---CSEKYNNIPAV-----

Lgig020_1 IVFTRKDYLK--------------------------------------------------

Lgig021_1 IVFTGKHDK--KRFSEYL---PRIPYFLLDCK----------------------------

Lgig022_1 YIFTGKDSLED-------------------------------------------------

Lgig023_1 VVFTGMDEFKG-TLDEYIQ-----------------------------------------

Mbre001_1 ------------------------------------------------------------

Nvec001_1 VILTFANKV---NQEDEIIDKEQWKSKV----------------TNVLMECAVSY-----

Oluc001_1 VVLTHAAAAPGISYEMYVAQSHIVQQTIRQAAGMRLMNPVN-------------------

Oluc002_1 LGFSHAQTTPGRPYEEFVNAVEQYRKAIRSTLNPNL------------------------

Ppat001_1 VVFTGGDDLED-SLDDFLEQPAYLRKFLEKCGDRKVLFENKTKRKAKQTDDLLRIIDDML

Ppat001_2 ------------------------------------------------------------

Ppat002_1 VVFTHAEIHL-VTYSEFVSRSAALRNIILKESRKTVNTKV--------------------

Ppat003_1 VVLTHASSAPGLSYEMFVAQSHVVQQTIRQAAGMRLMNPVNGQ-----------------

Ppat004_1 VVITYRNKD-----------------------------------LTDDEKVMFQKVDFE-

Ppat005_1 VVLTHASSAPGLSYEMFVAQSHVVQQTIRQAAGMRLMNPVNGQ-----------------

Ppat006_1 VVLTHASKAPGMSYDYFVAQSHFVQQTIRQAAGARLQNPVN-------------------

Ppat007_1 IALTHAQLSPGVDYTEFVNNSAALRAAIRQEAGKKSEGEVQNSTSF--------------

Ppat008_1 VLITHKNQR-----------------------------------WLNTQ------IRDL-

Ppat009_1 IVLTHAQFSPGVNYTEFVEKSAALQAAIRQEAGKK-------------------------

Ppat010_1 VLITHQNRA-----------------------------------WLDSK------TVDI-

Skow001_1 VLFTRKDQLED-TLTELLDDPAYMKSILRECNNRAIAFDNKSKVIQQQRDELIMMIDEMK

Skow001_2 VLFTRKDALTN-TLDNFLEEPKDLSDLLAKCNKRVIAFDNRTEEKNEQIRELVQKAEKMK

Skow001_3 VLFTRKDALTN-TLDKFLEEPKDLSDLVTTCNNRVIAFDNRTKEKNEQIRELVQKVEKMK

Skow002_1 VLFTRKDALTN-TLDEFLKEPEDLSDLLAKCNKRVIAFDNKTKVKKKQIQELVQKVEQIK

Skow003_1 ILFTRKDQLDK-SLADFLEEPSYMKHLLIDCNNRVLAFDNRTNVKEQQTAELVRLVDKTR

Skow004_1 ILFTRKDQLDK-SLADFLKEPSYLKHLLIDCNNRVLAFDNRTNVKEQQTAELVRLVDKTR

Skow005_1 ILFTRKDQLDK-SLADFLEEFSYLKHLLIDCNNRVLAFDNRTNVKEQQTAELVRLIDKTR

Skow006_1 -------------------------------------------------------IDKY-

Amil001_1 ---------GDN------------

Amil002_1 ------------FE----------

Amil003_1 ------------LQEMQPRA----

Amil004_1 ------------FRKLTQCF----

Amil005_1 NSFSKPQY----------------

Amil006_1 EERKVSFTTDDRVTDAVDYV----

Atha011_1 RSKCGEAYTDDTYHMIKEESEKLR

Atha012_1 KETGGNPFTDTMHRRIQEEAARVK

Atha013_1 KQNNNIPYTDEMYHMIKEENERHK

Atha014_1 KQNNGKPYSDELFHELQEEAIKLR

Atha015_1 RL-NGKSYMADLSHEIRENETAFQ

Atha016_1 KQNNGKPYSDELFHELQEEAIKLR

Atha017_1 KSTSGIPFTDEMHRKIQKEAETLR

Atha018_1 KKNNGKSYMADLSHELRENEATIK

Atha019_1 EQTGGIPYTYQLHRKIKEENERLR

Atha020_1 ERNEGKPFRGKMYLEIKEETEWLK

Atha021_1 NLNGGKAFTEENDLNEKRQGEMLM

Atha022_1 KKNDEKPYTEDMYRNIKVNTFLIA

Atha023_1 QQNGGIPYTENMHRKIKFKNLKYS

Atha024_1 RKNNNKPFLFDLSHESMESEAVVD

Atha024_2 ------------------------

Atha025_1 KNNGGKPYTNQMHRMIKEKGDKLR

Atha026_1 ------------------------

Atha027_1 ------------------------

Atha028_1 ------------------------

Atha029_1 ------------------------

Atha030_1 ------------------------

Atha031_1 ------------------------

Atha032_1 ------------------------

Atha033_1 ------------------------

Atha034_2 ------------------------

Atha035_1 ------------------------

Bflo001_1 RSNGGVPFRNSIFQEGQKEKDKIK

Bflo002_1 RSNGGVPFRDITFHEGQHEKDKII

Bflo003_1 AGTKNQPYNNKYTKIASEKFELLA

Bflo004_1 GEQAKIPFKDVIFAEGQHEKKRQE

Bflo005_1 ------------------------

Bflo006_1 TDTDNKPYNNKYTKMVSSM-----

Bflo007_1 AGTKNQPYNNRYTKYASEMFEEAQ

Bflo008_1 KEEGV--YNDD-------------

Bflo009_1 KRRKG-PY----------------

Bflo010_1 DQTKGPYKS---------------

Bflo011_1 IENGGPY-TDDLFQEGK-------

Bflo012_1 KENGI--YTDNLFEEGRKEMRIQE

Bflo013_1 EINNGPY-SDGLLRKEEDVQ----

Bflo014_1 EKNEG-PYIDDHFRE---------

Ddis001_1 ------------------------

Drer001_1 KQNQNSYYNYHMFEMANELNNVRK

Drer002_1 KEEYKEF-----------------

Drer002_2 EENKGEVS---TFLEPQMEKLKYD

Drer002_3 KTNGDSYYSCKMFREMEREIQEKQ

Drer002_4 KSNEGKHFTNEMFQEAEVSVDRRI

Drer003_1 IERHEEFIMGHG------------

Drer003_2 ---KTEPS---TLMRARHELA---

Drer003_3 EENKGQVS---TFLELQMEKLKHD

Drer003_4 KANGGSYYSCKRFRDIERDRQNKE

Drer003_5 QSNQAEHFTNEMFEKIKISINKRE

Drer004_1 SNNQGVYFTNSMFEEAEMSIKKKM

Drer005_1 KDEEVEFIMEET------------

Drer005_2 EENDRRFS---AFLEAQMEKEETR

Drer005_3 MKNGGSYYSCKIFRQMEREKQEAQ

Drer006_1 KQNDGQHY-SEIYSTPRTKSKEKK

Drer006_2 KDNGGEHYTNEMYKETQRKKMLTK

Drer007_1 QENQEAYTTSEV------------

Drer007_2 ---NTKPS---MYMTAQQNRRGEL

Drer007_3 TNNQGGYFTNSMFEEAEMYIKKKM

Drer008_1 KGNKQHYTRKDV------------

Drer008_2 ---KTKPS---MFVKALENRRRDL

Drer008_3 SNNQSGYFTNSMFEEAEMSIKKKM

Drer009_1 KGNKQHFTRKDV------------

Drer009_2 ---KTKPS---MFVKALENRRRDL

Drer009_3 SNNQSGYFTNSMFEEAEMSIKKKM

Drer010_1 AVNGGECYTQEMFREAEEKIRQQK

Drer011_1 KYTKDYYTLEDA------------

Drer011_2 ---KTKTSM---YVKAQENRRREA

Drer011_3 SNNQGGYFTNDMFEEAEMSIKKKM

Drer012_1 ---KTEPS---MYVKAQESRRCET

Drer012_2 SNNQGGYFTNEMFEEAEMSIKKKM

Drer013_1 ------------------------

Drer013_2 EENKGQVS---TFLELQMEKLKHD

Drer013_3 KANGGKRYETEIKEKVEQERSHLQ

Drer014_1 NNNQGGYFTNDMFEEAEMSIKKKM

Drer015_1 WDNKSSFYNDQMFQEVEKAFRLKQ

Drer015_2 ------------------------

Drer016_1 QENQEAYTTSEV------------

Drer016_2 ---KTKPS---MYMTAQQNRRGEL

Drer016_3 SNNQGGYFTNSMFEEAEMSIKKRM

Drer017_1 QENQDAYTTSEV------------

Drer017_2 ---KTKPS---MYMTAQQNRRGEL

Drer017_3 SNNQGGYFTNSMFEEAEMSIKKRM

Drer018_1 KRNKQHYTRKDV------------

Drer018_2 ---KTKTSK---FVKAQKNRRREA

Drer018_3 SNNQGRYFTNDMFEEAEMSIKKKM

Drer019_1 AVNEGGYYTNEMFQQVEKNIREEQ

Drer020_1 ------------------------

Drer020_2 AENGGSFYTNEMFQLVEKNIREEQ

Drer020_3 ------------------------

Drer021_1 EENGGHPRVLRL------------

Drer021_2 WDNNGSFYNDQMLQVFNKER-EYK

Drer022_1 EENRGHDKPPGL------------

Drer022_2 WDNKSSFYNDKMFQEAERALRLVQ

Drer023_1 EENRGHDKPPGL------------

Drer023_2 WDNKSSFYNDKMFQEAERALRLVQ

Drer024_1 KANGGSFFSCKMIREMEREKQEQQ

Drer025_1 RFNGGAYYTNDMLQEAERAIEEEK

Drer026_1 EGNGGNFK----HMSKDKIIF---

Drer026_2 EENEGAHFSSEIFEEIQKRIEEIQ

Drer027_1 AKNGGSFNTFKMFRQMEREQ-EQQ

Drer028_1 -----------------------P

Drer028_2 EEN--SFT---MFLQAQIELRNKH

Drer028_3 AENGGGYYTNEMFQQVEKNIRE--

Drer028_4 ------------------------

Drer029_1 KANGGSFYSCKMFREMEREKQQQQ

Drer029_2 ------------------------

Drer030_1 KANGGSFYSCKMFREMEREKQEQQ

Drer031_1 KANGGSFYSCKMFREMEREKQEQQ

Drer032_1 TGNEGQHYISEILER-ERAIEAEK

Drer033_1 TGNGGQHYTSEMLERAERANEEER

Drer034_1 GKNKSHQKQPRP------------

Drer034_2 CDNNSSFYNNQMFPEAEKALRLVH

Drer035_1 AVNGGGFYTNEMFQQVEKNIREEQ

Drer036_1 NNNQGGYFTNDMFEEAEMSIKKKM

Drer037_1 KANGGSFYSCKMFREMEREKQEQQ

Drer038_1 VENGGQHYTNEMYEEAQEKIELDA

Drer039_1 KANGGSFYSCKMFREMEREKQEQQ

Drer040_1 TGNGGQHYTSEMLEKVERAIEKEK

Drer041_1 QQNGGGHYSNQMYEDAHRFEFKKF

Drer042_1 PERRNETTEQII------------

Drer042_2 DENGGSCYSNKMFSDEESRTC---

Drer043_1 SNNQGRYFTNIMFEEAEMSIKKKM

Drer043_2 ------------------------

Drer044_1 IQNENRHFSSEVTQIMQQAQRIIE

Drer044_2 ------------------------

Drer045_1 QENQEAYITSEV------------

Drer045_2 ---KTKPS---MYMTAQQNRRGEL

Drer045_3 SNNQGGYFTNSMFEEAEMSIKKRM

Drer046_1 KANGGSFYSCKMFREMEREKQEQQ

Drer046_2 ------------------------

Drer046_3 NYSDTAA------GLRKI------

Drer046_4 QQNGGGHYTNQMFEDAQRFRREEE

Drer047_1 EENEMRHYTIDMFKKAPISKTGKR

Drer048_1 EENRGGVS---TFLEAQMSKVKYE

Drer048_2 KANGGSFYSCKMFREMEREKQEQQ

Drer048_3 NYSDTAA------G----------

Drer048_4 QQNGGGHYSNQMYEDALRFRQEER

Drer049_1 KANGGSFYSCKMFREMEREKQEQQ

Drer050_1 QQNGGGHYSNEIYKDVQETSA---

Drer051_1 EENRGGFS---TFLEAQMTKMKYK

Drer051_2 KANGGSFYSCKMFREMEREKQEQQ

Drer052_1 QSNDGRS-----------------

Drer052_2 EENREEVS---TFLEAQMKKLHYE

Drer052_3 KANGGSFYSCKMFRQIERKKQEQQ

Drer052_4 ------------------------

Drer053_1 ERNGGKHYTNEMYRKVQKKNEWLA

Drer054_1 QQNGGAHYSNQMYEDAHRFRQRNE

Drer055_1 KANGGSFYSCKMFREMEREKQEQQ

Drer055_2 ------------------------

Drer055_3 NYSDTAA------GLRK-------

Drer055_4 QQNGGGHYTNQMFEDAQRFRREEE

Drer055_5 KANGGSFYSCKIEEELKREIREGE

Drer056_1 KANGGSFYSCKMFREMERKKQEQQ

Drer056_2 ------------------------

Drer057_1 EENRGGFS---TFLEAQMIKMKYE

Drer057_2 KANGGNFYLCKIFREMEREKQEHQ

Drer058_1 EENRGGFS---TFLEVQMKKMEYE

Drer058_2 KANGGSLYSCKMFREMEREKQEQQ

Drer059_1 ------------------------

Drer059_2 EENGGEHFSSEMFEEIQKRIEEIQ

Drer060_1 KKNKGEHYTNEMYHDAQKKLNRER

Drer061_1 ------------------------

Drer061_2 EENGGQFYTNEMYMEAQKQIEEEE

Drer062_1 EENRGDFS---TFLEVQMEKTEYE

Drer062_2 KANGGSFYSCKMFREMEREKQEQQ

Drer063_1 EENGGQHYTNEMYKKAQEKIESEA

Drer064_1 TANGGQFYSSDSYQGVELMLKTKE

Drer065_1 RSNDGRHI--DS------------

Drer065_2 EENRGGVS---TFLEAQMENMKYE

Drer065_3 QLNGGGHYTNQMYEDALRLRQEEE

Drer066_1 KANGGSFYSCKMFREMEREKQEQQ

Drer066_2 ------------------------

Drer067_1 EENRGGVS---TFLEAQMENMKYE

Drer067_2 KANGGSFYSCKMFREMEREKQEQQ

Drer068_1 KANGGSFYSFKMFREMERENQEQQ

Drer069_1 RMNEDKYYTSDMFQHVEKMLKDKE

Drer070_1 EENGGQYYTDEMFQEAQRKINRGQ

Drer071_1 EENGGQYYTDEMFQEAQRKINRGQ

Drer072_1 KVANGA------LQVKRPKPLPVS

Drer072_2 QQNGGGHYSNQMYEDAQRARQEEE

Drer073_1 TANEGKHFTNQYYEDVKLKLKSKE

Drer074_1 TANEGKHFTNQYYEDVKLKLKSKE

Drer074_2 ------------------------

Drer075_1 DTNKEVN-TNGTFERAPRMSN---

Drer075_2 ------------------------

Drer076_1 IQNENRHFSSEVTQIMQREERGDA

Drer076_2 ------------------------

Drer077_1 TANGGGFFTNDSYQNVELMLKTKE

Drer078_1 TVNGEEYYTNDDLRAAERAIEEEK

Drer079_1 Q-EKE-FYTNEMYEYAQRNTE---

Drer080_1 ENNGNKHYTINNTQKQQMLKDGMK

Drer081_1 GKRSKKYYVRTKREQSKSWKQ---

Drer081_2 SGNGGHFYTNEMLEKAENAIEEEK

Drer082_1 EFNQG-YYTNEMYERAHTNTE---

Drer083_1 KSNEGRHIQEAY------------

Drer083_2 EENRGGVS---TFLEVQMKKKKYE

Drer083_3 KTNGGSFYSCKMFREMEREKQEQQ

Drer083_4 ------------------------

Drer084_1 EINQG-CYTNEMYEHAQRYTD---

Drer085_1 KDNKNSCYTNELLQMVEEEIQEEV

Drer086_1 KKHTEGFNT---------------

Drer086_2 -QGNAENYPKKKQK------DKDC

Drer087_1 ----KGCYTNKMYEYAHLNIE---

Drer088_1 KDNKDSCYTNELLQTVEEEIQQEV

Drer089_1 KDNKDSCYTNELLQTVEEEIQQEV

Drer090_1 PYLDTMG--EKIWQLKRKIP---A

Drer090_2 QQNGGGHYTNQMYEDAQIFRQEEE

Drer090_3 ------------------------

Drer091_1 E-MKG-CYTNELYECAQIGIE---

Drer092_1 QQNGGGHYTNEMHEDTQKVREEEK

Drer093_1 --NKNGCYTNELMLEIEEDIQGEM

Drer094_1 NYLDTMR--ADM------------

Drer094_2 QQNGGGHYTNQMYEDAQRARQEEE

Drer095_1 QQNGGGHYTNQMYEDAQRFRQEQE

Drer096_1 RSNEGRHIQRDS------------

Drer096_2 ------------------------

Drer096_3 QQNGGGHYTNQMFKDAQRLLEEER

Drer097_1 AINGKSCYTSSFYPATERKIRKKM

Drer098_1 RSNEGRHIQRDS------------

Drer098_2 ------------------------

Drer098_3 QQNGGGHYTNQMFKDAQRLLEEER

Drer099_1 RE-----FV---------------

Drer100_1 QQNGGGHYTNEMFEDALRARQEEE

Hmag001_1 ------------------------

Hmag002_1 PDSQGNAI----------------

Hsap001_1 RENKEGCYTNRMYQRAEEEIQKQT

Hsap002_1 QCNEGAYFSDDIYKDTEERLQEEV

Hsap002_2 ------------------------

Hsap003_1 REREGSFHSNDLFLDAQLL-RTGA

Hsap004_1 LEHKGAHYSNEVYELAQVLRAPEE

Hsap005_1 WENEGDYYSNKAYQYTQQNFLELQ

Hsap006_1 MEKNGDHYTNGLYSLIQRSKGVGS

Hsap007_1 NTNGGPYHVNFKTEGSRFQDVEAA

Hsap007_2 HQNGNKH-C--VFR-------EKE

Hsap007_3 KESGWSGYTQENVSNVQEMSQAEK

Hsap008_1 KENCYQVT----FK----------

Lgig001_1 EENGREFYSEDLLNLVEERIEEEV

Lgig002_1 EQNSGNFYTQDMFEATEKVIQQEI

Lgig003_1 EENGREFYSGDLWNLVEERIEDEI

Lgig004_1 EENGREFYSGDLLNLVEERIEEEI

Lgig005_1 EVNGDQVYSQEAF-----------

Lgig006_1 EQNSGNFYTQDMFEATEKVIQQEI

Lgig007_1 ESHSYKFYTAEMFKVADTILKKEV

Lgig008_1 ESHSYKFYTAEMFKVADTILEKEV

Lgig009_1 NSLGSLHYTNEMFNQANE------

Lgig010_1 ------------------------

Lgig011_1 EMNRHRHE----------------

Lgig011_2 ------------------------

Lgig012_1 LQNKKQHYTDELQHQLKQSINQRE

Lgig013_1 NDRNLNHDTNNNSSNSERNS----

Lgig014_1 SKNKKKHYTDKM------------

Lgig015_1 ------------------------

Lgig016_1 ------------------------

Lgig017_1 ------------------------

Lgig018_1 ------------------------

Lgig019_1 ------------------------

Lgig020_1 ------------------------

Lgig021_1 ------------------------

Lgig022_1 ------------------------

Lgig023_1 ------------------------

Mbre001_1 ------------------------

Nvec001_1 ------------------------

Oluc001_1 ------------------------

Oluc002_1 ------------------------

Ppat001_1 LKNGDNPYTNELFKEAQTEQGKSK

Ppat001_2 ------------------------

Ppat002_1 ------------------------

Ppat003_1 ------------------------

Ppat004_1 ------------------------

Ppat005_1 ------------------------

Ppat006_1 ------------------------

Ppat007_1 ------------------------

Ppat008_1 ------------------------

Ppat009_1 ------------------------

Ppat010_1 ------------------------

Skow001_1 QRNGNKPFNNDLTQRIKQAVDSDK

Skow001_2 KDNGNAPFKNQYTDAIKRKIAEDQ

Skow001_3 KDNGNAPFKNQYTDAIKRKIAEDQ

Skow002_1 EDNGDTPFKNQYTEAIKSKIAEDQ

Skow003_1 ASNGNKPFTNDITRRVKEAVEDDR

Skow004_1 ASNGNKPFTNDITRRVKEAVEDDR

Skow005_1 ASNGNKPFTNDITRRVKEAVEEDR

Skow006_1 ------------------------
